# FA-MFMR: a multivariable functional Mendelian randomization method that accounts for correlated longitudinal exposures via factor augmentation

**DOI:** 10.1093/bioinformatics/btag725

**Published:** 2026-10-08

**Authors:** Dong Chen, Junrong Deng, Siyuan Shen, Yuyang Zhou, Hanyu Cui, Yu Qiu, Qiming Li, Yue-Qing Hu

**Affiliations:** State Key Laboratory of Genetics and Development of Complex Phenotypes, Institute of Biostatistics, School of Life Sciences, Fudan University, Shanghai, Yangpu District 200438, China; State Key Laboratory of Genetics and Development of Complex Phenotypes, Institute of Biostatistics, School of Life Sciences, Fudan University, Shanghai, Yangpu District 200438, China; State Key Laboratory of Genetics and Development of Complex Phenotypes, Institute of Biostatistics, School of Life Sciences, Fudan University, Shanghai, Yangpu District 200438, China; State Key Laboratory of Genetics and Development of Complex Phenotypes, Institute of Biostatistics, School of Life Sciences, Fudan University, Shanghai, Yangpu District 200438, China; State Key Laboratory of Genetics and Development of Complex Phenotypes, Institute of Biostatistics, School of Life Sciences, Fudan University, Shanghai, Yangpu District 200438, China; State Key Laboratory of Genetics and Development of Complex Phenotypes, Institute of Biostatistics, School of Life Sciences, Fudan University, Shanghai, Yangpu District 200438, China; State Key Laboratory of Genetics and Development of Complex Phenotypes, Institute of Biostatistics, School of Life Sciences, Fudan University, Shanghai, Yangpu District 200438, China; State Key Laboratory of Genetics and Development of Complex Phenotypes, Institute of Biostatistics, School of Life Sciences, Fudan University, Shanghai, Yangpu District 200438, China; Shanghai Center for Mathematical Sciences, Fudan University, Shanghai, Yangpu District 200438, China

## Abstract

**Motivation:**

Mendelian randomization (MR) is widely used for causal inference using genetic data, yet most existing MR methods treat exposures as static and have limited capacity to analyze longitudinal measurements. In many biomedical studies, multiple exposures evolve over time, are strongly correlated, and share unobserved temporal structure. Ignoring these features can lead to unstable estimation and reduced power.

**Results:**

We propose factor-augmented multivariable functional Mendelian randomization (FA-MFMR) for estimating time-varying causal effects of multiple longitudinal exposures. FA-MFMR integrates genetic instruments with functional representations of exposure trajectories and a low-dimensional factor structure to capture shared temporal patterns. By separating common temporal variation from exposure-specific components, it mitigates multicollinearity, improves estimation stability, and accommodates sparse causal structures in which some exposures have no effect throughout the observation period. Simulations demonstrate lower estimation error and more efficient uncertainty quantification than competing approaches, particularly for highly correlated and sparsely observed longitudinal exposures. Applied to longitudinal biomarkers in a Parkinson’s disease cohort, FA-MFMR characterizes age-dependent patterns in the estimated trajectory-level coefficient functions while highlighting uncertainty in localized effects.

**Availability and implementation:**

The developed R package and code to reproduce all the results are available at https://github.com/YQHuFD/FA-MFMR.

## 1 Introduction

Mendelian randomization (MR) uses genetic variants as instrumental variables for causal inference under appropriate identification assumptions (Smith and Ebrahim 2003, Schadt *et al*. 2005, Smith 2006, Lawlor *et al*. 2008, Burgess *et al*. 2013, Bowden *et al*. 2015, Morrison *et al*. 2020, Wang *et al*. 2021, Xue *et al*. 2021, Yao *et al*. 2026). The causal interpretation of an MR estimate depends on the target estimand and its identifying assumptions (Yao *et al*. 2026). Conventional MR typically represents an exposure by a scalar measurement or summary and therefore does not explicitly characterize temporal variation, although both exposure levels and genetic associations may vary across the life course (Labrecque and Swanson 2019, Richardson *et al*. 2020, Jiang *et al*. 2021, Shi *et al*. 2022).

Longitudinal and functional MR methods use repeated exposure measurements to investigate time-varying or trajectory-level effects (Cao *et al*. 2016, Sanderson *et al*. 2022, Richardson *et al*. 2023, Tian and Burgess 2023, Michael *et al*. 2024, Tian *et al*. 2024, Wu *et al*. 2025), whereas multivariable MR jointly considers multiple genetically proxied exposures and their mutually adjusted effects (Rees *et al*. 2017, Grant and Burgess 2021, Sanderson *et al*. 2021, Lin *et al*. 2023). A broader overview of MR estimands, identification, and inference is provided by Yao *et al*. (2026). However, jointly modeling multiple correlated longitudinal exposure trajectories remains relatively underexplored.

When extending MR to settings with multiple longitudinal exposures, several methodological challenges typically emerge (Power *et al*. 2024). First, multiple time-varying exposures are often correlated and may share latent temporal structures driven by unobserved biological processes or environmental factors (Ding *et al*. 2022, Palmer and Jensen 2022). Ignoring such dependence can lead to unstable estimation and inflated uncertainty in functional causal effect estimates. Second, in realistic settings, not all exposures exert causal effects on the outcome, and many time-varying effects are structurally zero over the entire follow-up period. Accurately identifying these zero-effect functions is essential for valid inference but remains difficult when exposures are high-dimensional, sparsely observed, and measured with error (Qu *et al*. 2018, Swanson *et al*. 2018). Third, conventional functional MR approaches typically estimate each exposure-specific effect separately or rely on marginal functional representations, limiting their ability to borrow information across exposures and to distinguish shared temporal patterns from exposure-specific causal signals.

We propose Factor-Augmented Multivariable Functional Mendelian Randomization (FA-MFMR), which combines multivariable functional MR with low-rank factor augmentation to separate shared temporal structure from exposure-specific variation. By modeling shared temporal dependence through a low-dimensional factor structure and using the remaining instrument-supported variation to distinguish exposure-specific structural coefficients, FA-MFMR addresses the instability induced by strong cross-exposure dependence. At the same time, the factor-augmented representation enables efficient borrowing of information across exposures, improving estimation accuracy in high-dimensional and sparsely observed settings. Importantly, FA-MFMR naturally accommodates sparse causal architectures by allowing some exposure-specific time-varying effect functions to be identically zero, facilitating the identification of truly causal longitudinal exposures. Together, these features lead to more stable, efficient, and interpretable estimation of multivariate time-varying causal effects within the MR framework. We demonstrate its performance through extensive simulation studies. We further apply FA-MFMR to longitudinal Parkinson’s disease data, where it yields more stable and interpretable estimates of multivariate time-varying causal effects and reveals dynamic exposure–outcome relationships that are obscured by existing MR approaches. Together, these results illustrate the practical utility of FA-MFMR for causal inference with complex longitudinal exposures.

## 2 Materials and methods

### 2.1 Model

We consider *K* time-varying exposures $X^{\left(k\right)}(t),k=1,\ldots ,K$, each observed longitudinally over t∈[0,T]. The data consist of *n* independent individuals, indexed by i=1,…,n. Following the convention in time-varying instrumental variable analysis, we treat each exposure trajectory $X_{i}^{\left(k\right)}(t)$ as a realization of an underlying stochastic process indexed by continuous time, even though measurements are collected at discrete time points. The outcome $Y_{i}$ is measured at the terminal time *T*, ensuring that all exposure trajectories on [0,T] temporally precede the outcome. Let $Z_{i}$ denote a vector of *J* independent genetic variants, which serve as instrumental variables for the time-varying exposures.

To make the longitudinal causal estimand explicit, let $X_{i}(\cdot )=\left\{X_{i}^{\left(1\right)}(\cdot ),\ldots ,X_{i}^{\left(K\right)}(\cdot )\right\}$ denote the collection of exposure trajectories for individual *i*, and let $x(\cdot )=\left\{x^{\left(1\right)}(\cdot ),\ldots ,x^{\left(K\right)}(\cdot )\right\}$ denote a fixed collection of admissible intervention trajectories in $\prod_{k=1}^{K}L^{2}[0,T]$. We use $Y_{i}\{x(\cdot )\}$ to denote the potential terminal outcome that would be observed if the longitudinal exposure histories are set to x(·). We assume consistency, no interference, and the following structural functional linear model:


$$Y_{i}\{x(\cdot )\}=\beta_{0}+\sum_{k=1}^{K}\int_{0}^{T}\beta^{\left(k\right)}(t)x^{\left(k\right)}(t)dt+g\left({\vec{U}}_{i},\varepsilon_{i}\right),$$

where ${\vec{U}}_{i}=\left\{U_{i}(t):0\leq t\leq T\right\}$ denotes an unmeasured confounder trajectory that may include both time-invariant background variables and time-varying stochastic processes affecting the longitudinal exposures and the outcome, and $\varepsilon_{i}$ denotes idiosyncratic outcome noise. Under consistency, $Y_{i}=Y_{i}\left\{X_{i}(\cdot )\right\}$, so that the observed-data structural model is


(1) $$Y_{i}=\beta_{0}+\sum_{k=1}^{K}\int_{0}^{T}\beta^{\left(k\right)}(t)X_{i}^{\left(k\right)}(t)dt+g({\vec{U}}_{i},\varepsilon_{i}),$$

To interpret $\beta^{\left(k\right)}(t)$, consider an admissible perturbation $h^{\left(k\right)}(\cdot )\in L^{2}[0,T]$ to the *k*-th exposure trajectory while holding the other exposure trajectories fixed. Under the structural model, the corresponding causal contrast i*s*


$$\begin{array}{c} \Delta_{k}\left\{h^{\left(k\right)};x\right\}=Y_{i}\left\{x^{\left(1\right)}(\cdot ),\ldots ,x^{\left(k\right)}(\cdot )+h^{\left(k\right)}(\cdot ),\ldots ,x^{\left(K\right)}(\cdot )\right\}\\ -Y_{i}\left\{x^{\left(1\right)}(\cdot ),\ldots ,x^{\left(k\right)}(\cdot ),\ldots ,x^{\left(K\right)}(\cdot )\right\}=\int_{0}^{T}\beta^{\left(k\right)}(t)h^{\left(k\right)}(t)dt. \end{array}$$

Thus, $\beta^{\left(k\right)}(t)$ defines the trajectory-level causal linear functional for exposure *k*. Because the time-invariant instruments generally perturb combinations of the exposure trajectory, $\beta^{\left(k\right)}(t)$ should not, without additional assumptions, be interpreted as the effect of changing the exposure only at time *t*.

Figure 1A presents the causal structure assumed in our formulation, depicting the roles of the genetic variants, the longitudinal exposure processes, potential unmeasured confounders, and the outcome.

**Figure 1:**
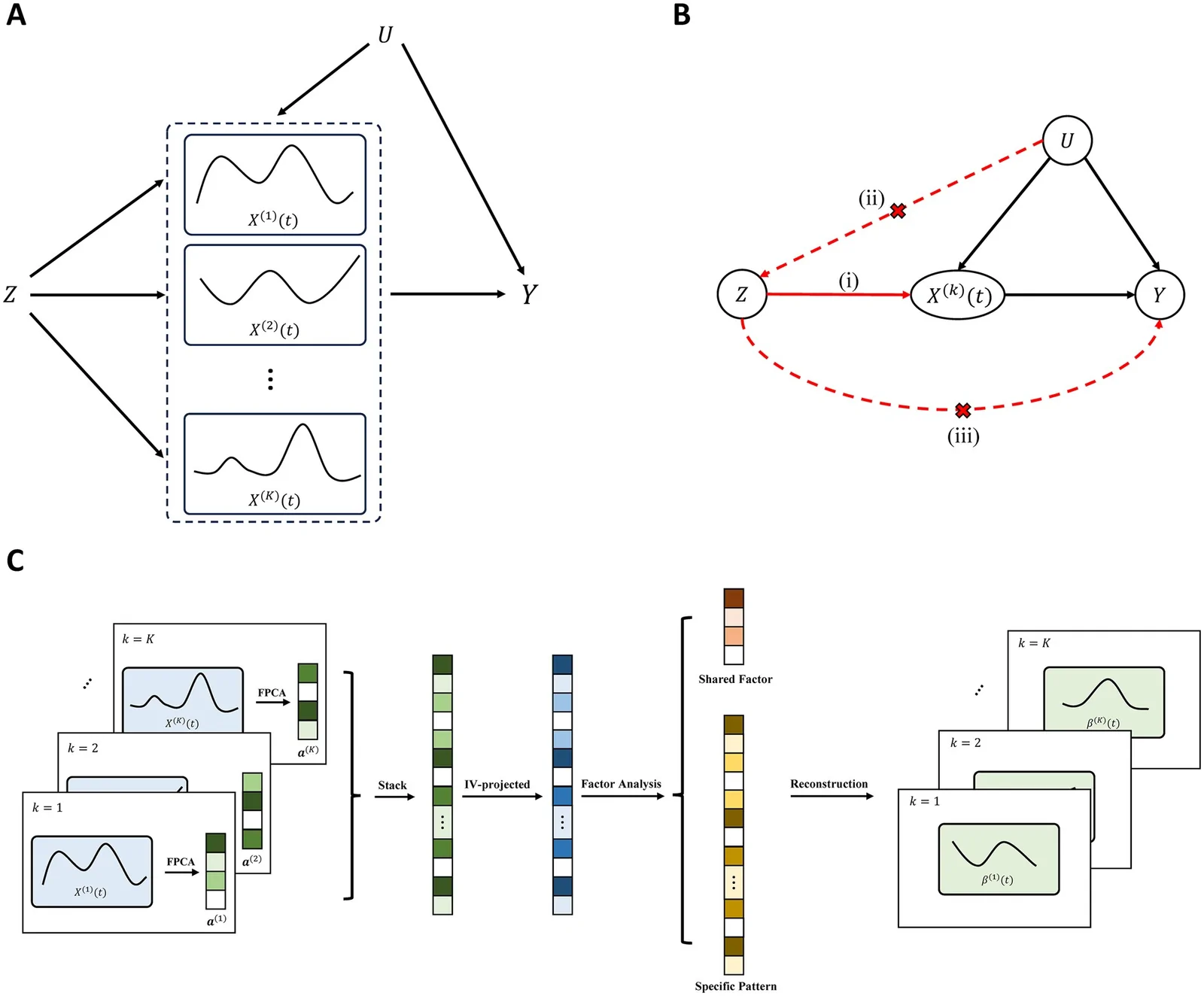
Causal model and factor-augmented estimation framework for multivariate functional Mendelian randomization. (A) Schematic representation of the assumed causal structure, where multiple time-varying exposure trajectories $X^{\left(k\right)}(t),k=1,\ldots ,K$, are influenced by genetic variants *Z* and potentially correlated through unmeasured confounders *U*. The outcome *Y* is measured at the terminal time *T* and depends on the entire exposure histories through their time-varying causal effect functions $\beta^{\left(k\right)}(t)$. (B) Directed acyclic graph illustrating the instrumental variable assumptions in the time-varying setting: genetic variants affect the outcome only through the exposure trajectories (relevance), are independent of unmeasured confounders (independence), and have no direct effect on the outcome beyond the exposure pathways (exclusion restriction); dashed paths indicate prohibited causal links. (C) Overview of the factor-augmented multivariate functional Mendelian randomization (FA-MFMR) estimation procedure. Each exposure trajectory is first represented using functional principal component analysis, with instrument-projected scores stacked across exposures. Factor analysis is then applied to extract shared latent temporal structure and exposure-specific components, which are subsequently used to reconstruct the time-varying causal effect functions $\beta^{\left(k\right)}(t)$.

Within this framework, the instrumental variable approach requires that the genetic variants serving as instruments satisfy the standard IV assumptions in this time-varying setting:

- *Relevance*: each IV should be associated with at least one exposure trajectory.
- *Independence*: each IV should be independent of the unmeasured confounder trajectory ${\vec{U}}_{i}$, including both time-invariant and time-varying factors that confound the longitudinal exposure-outcome relationships.
- *Exclusion restriction*: each IV should influence the outcome only through the exposure trajectories, with no direct effect on the outcome beyond the pathways encoded in Equation (1).

These assumptions correspond to the arrows in Fig. 1B. Violations of independence or exclusion restriction, including direct SNP–outcome pathways due to horizontal pleiotropy, invalidate the IV exogeneity condition and may bias the estimated coefficient functions.

### 2.2 Framework of FA-MFMR

Motivated by the structural causal formulation, FA-MFMR provides a practical estimation framework for recovering the time-varying causal effect functions $\beta^{\left(k\right)}(t)$ from longitudinal exposures that may be sparsely measured, potentially correlated, and partly driven by latent temporal structure, as illustrated in Fig. 1C. The full estimation strategy consists of functional representation of the exposures, instrument-based prediction of functional features, latent-factor augmentation, and reconstruction of the causal effect functions.

The sparsely and irregularly observed trajectories are treated as noisy evaluations of underlying square-integrable processes on [0,T]. After centering the exposure trajectories and outcome, we use the basis expansio*n*


(2) $$X_{i}^{\left(k\right)}(t)=\sum_{j=1}^{m_{k}}a_{ij}^{\left(k\right)}\gamma_{j}^{\left(k\right)}(t)+e_{i}^{\left(k\right)}(t),$$

where ${\left\{\gamma_{j}^{\left(k\right)}(t)\right\}}_{j=1}^{m_{k}}$ are exposure-specific orthonormal basis functions, $a_{ij}^{\left(k\right)}$ are the associated subject-level scores, and $e_{i}^{\left(k\right)}(t)$ is a mean-zero approximation error orthogonal to the span of the basis functions.

We obtain this representation using FPCA with the Karhunen-Loève expansion (Yao *et al*. 2005), where $\gamma_{j}^{\left(k\right)}(t)$ is the *j*-th eigenfunction and $a_{ij}^{\left(k\right)}=\int_{0}^{T}X_{i}^{\left(k\right)}(t)\gamma_{j}^{\left(k\right)}(t)dt$ is the corresponding score. In practice, PACE estimates the eigenfunctions and conditional-expectation scores from sparse or irregular measurements (Wang *et al*. 2016).

With $m_{k}$ retained FPCA components, define $M_{k}=\mathrm{span}\left\{\gamma_{1}^{\left(k\right)},\ldots ,\gamma_{m_{k}}^{\left(k\right)}\right\}$ as the retained functional space for exposure *k*. The component of the causal coefficient function represented in this space i*s*


$$\beta_{m_{k}}^{\left(k\right)}(t)=\Pi_{M_{k}}\beta^{\left(k\right)}(t)=\sum_{j=1}^{m_{k}}\eta_{j}^{\left(k\right)}\gamma_{j}^{\left(k\right)}(t),$$

where $\eta_{j}^{\left(k\right)}=\int_{0}^{T}\beta^{\left(k\right)}(t)\gamma_{j}^{\left(k\right)}(t)dt$. This representation has a direct connection to the trajectory-level causal estimand. Specifically, consider a perturbation of the *k*-th exposure trajectory that lies in the retained FPCA space, $h^{\left(k\right)}(t)=\sum_{j=1}^{m_{k}}c_{j}^{\left(k\right)}\gamma_{j}^{\left(k\right)}(t)$. Its causal contrast is


$$\Delta_{k}\left\{h^{\left(k\right)}\right\}=\int_{0}^{T}\beta^{\left(k\right)}(t)h^{\left(k\right)}(t)dt=\sum_{j=1}^{m_{k}}c_{j}^{\left(k\right)}\eta_{j}^{\left(k\right)}.$$

Hence, $\eta^{\left(k\right)}$ gives the coordinates of the trajectory-level causal functional along the retained FPCA directions. With finite FPCA truncation, functional directions orthogonal to $M_{k}$ are not represented in the subsequent finite-dimensional analysis. For notational simplicity, we continue to use $\beta^{\left(k\right)}(t)$ in the remainder of the main text to denote its approximation based on the retained FPCA representation.

Let $a_{i}^{\left(k\right)}={\left(a_{i1}^{\left(k\right)},\ldots ,a_{im_{k}}^{\left(k\right)}\right)}^{⊤}$ denote the score vector for *k*-th exposure, and let $p=\sum_{k=1}^{K}m_{k}$ be the total number of basis coefficients across all exposures. Collecting all coefficients into the stacked vector $a_{i}={\left(a_{i}^{(1)⊤},\ldots ,a_{i}^{(K)⊤}\right)}^{⊤},$ and similarly defining $H={\left(\eta^{(1)⊤},\ldots ,\eta^{(K)⊤}\right)}^{⊤},$ the structural model can be written in the retained FPCA representation a*s*


$$Y_{i}=\sum_{k=1}^{K}\sum_{j=1}^{m_{k}}a_{ij}^{\left(k\right)}\eta_{j}^{\left(k\right)}+{\tilde{\varepsilon }}_{i},$$

or equivalently,


(3) $$Y_{i}=H^{⊤}a_{i}+{\tilde{\varepsilon }}_{i}$$

where ${\tilde{\varepsilon }}_{i}=\sum_{k=1}^{K}\int_{0}^{T}\beta^{\left(k\right)}(t)e_{i}^{\left(k\right)}(t)dt+g\left({\vec{U}}_{i},\varepsilon_{i}\right)$.

Identification of the retained functional coefficients is based on the population IV moment condition. For centered instruments, define $\Gamma_{Za}=E\left[Z_{i}a_{i}^{⊤}\right]\in R^{J\times p}$. We require $E\left[Z_{i}{\tilde{\varepsilon }}_{i}\right]=0$ and $\mathrm{rank}\left(\Gamma_{Za}\right)=p,\sigma_{\text{min}}\left(\Gamma_{Za}\right)\geq c_{Z}>0$. The first condition is the functional-IV exogeneity condition, while the second requires sufficient instrument variation across the retained FPCA score directions. From Equation (3), $E\left[Z_{i}Y_{i}\right]=\Gamma_{Za}H$. Therefore, when $\Gamma_{Za}$ has full column rank, H is uniquely identified within the retained FPCA space. Thus, recoverable causal trajectory directions must be both represented by the retained FPCA scores and supported by instrument variation. If independence or the exclusion restriction is violated, then $E\left[Z_{i}{\tilde{\varepsilon }}_{i}\right]\neq 0$, and the moment equation no longer identifies H without additional restrictions. Moreover, genetic relevance may differ across FPCA-score directions; weakly supported directions reduce $\sigma_{\text{min}}\left(\Gamma_{Za}\right)$ and can lead to unstable or weak-instrument-biased estimation. This instability propagates to the reconstructed coefficient functions through ${\hat{\beta }}^{\left(k\right)}(t)=\sum_{j}{\hat{\eta }}_{j}^{\left(k\right)}\gamma_{j}^{\left(k\right)}(t)$.

As in traditional Mendelian randomization, the retained FPCA scores may be endogenous because the longitudinal exposure trajectories can depend on unmeasured factors that also affect the outcome. We therefore use the genetic instruments to extract the instrument-predictable variation in these retained scores. Let $A={\left(a_{1},\ldots ,a_{n}\right)}^{⊤}\in R^{n\times p}$ denote the matrix of retained FPCA scores and $Z={\left(Z_{1},\ldots ,Z_{n}\right)}^{⊤}\in R^{n\times J}$ the instrument matrix. At the sample level, the fitted score matrix is obtained through the linear projectio*n*


$$\hat{A}=P_{Z}A,\;P_{Z}=Z{\left(Z^{⊤}Z\right)}^{-1}Z^{⊤}.$$

We denote by ${\hat{a}}_{i}$ the *i*-th row of A. Thus, ${\hat{a}}_{i}$ represents the component of the retained FPCA score vector for individual *i* that is linearly predictable from the genetic instruments.

In multivariable Mendelian randomization settings involving multiple time-varying exposures, the underlying exposure processes $X^{\left(k\right)}(t),k=1,\ldots ,K,t\in \left[0,T\right]$, may exhibit complex and potentially unknown dependence structures. To accommodate the possibility of such unknown and potentially persistent latent structures among the adjusted exposure scores, and motivated by recent work on multivariate functional factor-augmented selection models (Ma *et al*. 2025), FA-MFMR introduces a latent factor representation for the matrix of estimated score vectors. The factor-augmented representation is given b*y*


(4) $${\hat{a}}_{i}=\Lambda f_{i}+u_{i},$$

where $f_{i}\in R^{r}$ collects *r* latent factors summarizing the dominant modes of shared variation across the multivariate functional exposures, $\Lambda \in R^{p\times r}$ is the loading matrix, and $u_{i}\in R^{p}$ represents idiosyncratic, exposure-specific variation not explained by the latent structure. A low-dimensional factor representation is reasonable when the dependence among the instrument-predictable FPCA scores is largely driven by a small number of shared modes, so that a few dominant directions capture the major cross-exposure covariance while sufficient idiosyncratic variation remains. This setting is plausible when multiple longitudinal exposures are jointly influenced by a limited number of shared biological or environmental processes; the latent factors are statistical representations of common dependence and need not correspond one-to-one to specific biological mechanisms. In practice, the latent factors, loadings, and idiosyncratic components are unobserved and are estimated from $\hat{A}$, yielding the corresponding sample-level estimates $\hat{\Lambda }$, ${\hat{f}}_{i}$ and ${\hat{u}}_{i}$.

We estimate the loading matrix $\hat{\Lambda }$ and factors $\hat{F}={\left({\hat{f}}_{1},\ldots ,{\hat{f}}_{n}\right)}^{⊤}\in R^{n\times r}$ by principal component analysis of $\hat{A}$ (Bai 2003), and obtain $\hat{U}=\hat{A}-\hat{F}{\hat{\Lambda }}^{⊤}$, where $\hat{U}={\left({\hat{u}}_{1},\ldots ,{\hat{u}}_{n}\right)}^{⊤}\in R^{n\times p}$. More specifically, the columns of $\hat{F}/\sqrt{n}$ are the eigenvectors of $\hat{A}{\hat{A}}^{⊤}$ corresponding to the top *r* eigenvalues, and $\hat{\Lambda }=n^{-1}{\hat{A}}^{⊤}\hat{F}$. We select the number of factors *r* using the eigenvalue-ratio criterion applied to the instrument-projected score matrix (Ahn and Horenstein 2013, Fan *et al*. 2020, Ma *et al*. 2025). Specifically, let $\lambda_{R}\left({\hat{A}}^{⊤}\hat{A}\right)$ denote the *R*-th largest eigenvalue of ${\hat{A}}^{⊤}\hat{A}$, $R_{\text{max}}$ be a prespecified upper bound for *R*, then *r* is determined by $\hat{r}=\underset{R\leq R_{\text{max}}}{\mathrm{argmin}}\frac{\lambda_{R+1}\left({\hat{A}}^{⊤}\hat{A}\right)}{\lambda_{R}\left({\hat{A}}^{⊤}\hat{A}\right)}$ for a given $R_{\text{max}}$. This criterion identifies the dimension preceding the largest relative drop in the eigenvalue sequence and therefore provides a data-driven estimate of the dominant shared factor dimension.

After obtaining the factor decomposition, substituting it into the basis-coefficient form of the structural model yield*s*


(5) $$Y=\hat{F}{\hat{\Lambda }}^{⊤}H+\hat{U}H+{\tilde{\varepsilon }}^{*},$$

where $Y={\left(Y_{1},\ldots ,Y_{n}\right)}^{⊤}$ and ${\tilde{\varepsilon }}^{*}={\left({\tilde{\varepsilon }}_{1}^{*},\ldots ,{\tilde{\varepsilon }}_{n}^{*}\right)}^{⊤}$. Equation (5) is a factor-augmented representation of the same coefficient vector H appearing in Equation (3). Both the shared-factor component $\hat{F}{\hat{\Lambda }}^{⊤}H$ and the idiosyncratic component $\hat{U}H$ therefore contribute to the structural effect represented by H.

To distinguish the exposure-specific coefficients in the presence of strong shared dependence, we partial out the estimated common-factor space, yieldin*g*


(6) $$(I_{n}-P_{\hat{F}})Y=(I_{n}-P_{\hat{F}})\hat{U}H+(I_{n}-P_{\hat{F}}){\tilde{\varepsilon }}^{*},$$

where $P_{\hat{F}}=\hat{F}{\left({\hat{F}}^{⊤}\hat{F}\right)}^{-1}{\hat{F}}^{⊤}$. Equation (6) shows that, although the shared-factor contribution remains part of the structural effect in Equation (5), estimation of the exposure-specific coordinates of H relies on the instrument-projected variation remaining after adjustment for the estimated common factors. Let S=supp(H). We require $\mathrm{rank}\left\{\left(I_{n}-P_{\hat{F}}\right){\hat{U}}_{S}\right\}=|S|$ and $\lambda_{\text{min}}\left[\frac{1}{n}{\hat{U}}_{S}^{⊤}\left(I_{n}-P_{\hat{F}}\right){\hat{U}}_{S}\right]\geq c_{U}>0$ with probability tending to one, together with ${\left\|\frac{1}{n}{\hat{U}}_{S}^{⊤}\left(I_{n}-P_{\hat{F}}\right){\tilde{\varepsilon }}^{*}\right\|}_{2}=o_{p}(1)$. These conditions ensure that the active coefficient directions remain identifiable after factor adjustment.

Under these conditions, factor residualization does not redefine H. The same coefficient vector appears in Equations (3) (5), and (6); Equation (6) changes the variation used to identify H, not its structural meaning. Thus, H retains its trajectory-level causal interpretation provided that the same structural coefficient vector applies to both the shared-factor and idiosyncratic components. If an active direction has insufficient residual variation after factor adjustment, however, that coordinate of H cannot be separately identified from Equation (6), although its contribution through $\hat{F}{\hat{\Lambda }}^{⊤}H$ remains part of the structural signal in Equation (5). In this case, the shared-factor component may identify only lower-dimensional combinations such as ${\hat{\Lambda }}^{⊤}H$.

Under the residual rank, relevance, and orthogonality conditions above, we estimate the same structural coefficient vector H from Equation (6) using SCAD regularization,


(7) $$\hat{H}=\underset{H\in R^{p}}{\text{argmin}}\left\{\frac{1}{2n}{\left\|\left(I_{n}-P_{\hat{F}}\right)(Y-\hat{U}H)\right\|}_{2}^{2}+P_{\lambda }(H)\right\}.$$

where $P_{\lambda }(H)$ denotes a SCAD-type sparsity penalty, and λ is the tuning parameter. Then we reconstruct the causal effect functions for each exposure by mapping the estimated basis-level coefficients back to the functional domain. For exposure *k*, the estimated causal effect function is reconstructed as


(8) $${\hat{\beta }}^{\left(k\right)}(t)=\sum_{j=1}^{m_{k}}{\hat{\eta }}_{j}^{\left(k\right)}\gamma_{j}^{\left(k\right)}(t),$$

which provides an estimator of the time-varying causal effect function.

## 3 Results

### 3.1 Simulation studies

We conduct simulation studies under a variety of controlled settings with varying sample sizes, exposure dimensions and latent complexity. Each simulation involves n∈{500,1000,2000} independent individuals and K∈{5,10} longitudinal exposures defined on the domain t∈[0,50], which represents a simulated age range. For every exposure *k*, we use six cubic B-spline basis functions with equally spaced internal knots, leading to a total of p=6K basis coefficients. The latent structure comprises r∈{1,2,3} latent factors.

The instrumental variables are generated to mimic genotype data. For each individual, we generate J=100 genetic instruments. Specifically, for each SNP *j*, an allele frequency $p_{j}$ is drawn from Unif(0.2,0.4) and genotypes are sampled as $Z_{ij}∼\text{Binomial}(2,p_{j})$ after which the matrix Z is column-standardized. Conditional on these instruments, the latent factors and the idiosyncratic component are generated using $F=Z\Pi_{F}+E_{F},\;U=Z\Pi_{U}+E_{U},$ where the loading matrices $\Pi_{F}\in R^{J\times r}$ and $\Pi_{U}\in R^{J\times p}$ have entries drawn from Unif(−2,2) and Unif(−1.5,1.5), respectively, and the noise matrices $E_{F}$ and $E_{U}$ contain i.i.d. $N(0,0.1^{2})$ and $N(0,0.2^{2})$ errors, respectively. The resulting F and U are standardized column-wise. The factor-augmented score matrix follows $A=F\Lambda^{⊤}+U,$ where $\Lambda \in R^{p\times r}$ has entries sampled from $N(0,0.5^{2})$. Let $A=\left(A^{\left(1\right)},\ldots ,A^{\left(K\right)}\right)\in R^{n\times p},p=6K$, where each $A^{\left(k\right)}\in R^{n\times 6}$ contains the six basis coefficients for exposure *k*.

To incorporate heterogeneous stochastic variation and realistic subject-level dependence, each exposure trajectory is generated a*s*


$$X_{i}^{\left(k\right)}(t)=\sum_{j=1}^{6}a_{ij}^{\left(k\right)}\gamma_{j}^{\left(k\right)}(t)+\alpha b_{ik}(t)+(1-\alpha )c_{i}(t)+\sigma_{X}m_{ik}(t).$$

Here, $b_{ik}(t)$ and $c_{i}(t)$ are standardized exposure-specific and shared Brownian paths, respectively, combined as $\alpha b_{ik}(t)+(1-\alpha )c_{i}(t)$ with α=0.4. Independent standardized measurement noise is added with $\sigma_{X}=0.1$. Each subject has $n_{i}∼\mathrm{Unif}\{5,\ldots ,15\}$ irregular visits, sampled more densely in the central 30% of the interval (probability 0.7) than near the boundaries (probability 0.3).

We consider the outcome model


$$\begin{array}{c} Y_{i}=\sum_{k=1}^{K}\int_{0}^{50}X_{i}^{\left(k\right)}(t)\beta^{\left(k\right)}(t)dt\\ +\sum_{k=1}^{K}\left(\alpha b_{ik}(50)+(1-\alpha )c_{i}(50)\right)+\sigma_{Y}\varepsilon_{Y}(50), \end{array}$$

where $\varepsilon_{Y}$ is an independent standardized Brownian-motion process, and scaled by $\sigma_{Y}=0.05$. This construction explicitly introduces time-varying unmeasured confounding: the processes $b_{ik}(t)$ and $c_{i}(t)$ evolve over time and contribute to both the longitudinal exposure trajectories and the outcome error. Consequently, the exposure trajectories are endogenous through shared subject-level temporal processes. The true exposure effect functions $\beta^{\left(k\right)}(t)$ for k=1,…,K are specified to include diverse temporal patterns:

1. Linear effect: $\beta^{\left(1\right)}(t)=2(t-0.5)$;
2. Quadratic effect: $\beta^{\left(2\right)}(t)=4{\left(t-0.5\right)}^{2}$;
3. Sinusoidal effect: $\beta^{\left(3\right)}(t)=\text{sin}(2\pi t)$;
4. Gaussian effect: $\beta^{\left(4\right)}(t)=\text{exp}(-\frac{{\left(t-0.5\right)}^{2}}{0.2^{2}})$.

All four functions are centered over [0,1] and rescaled to [0,50] to ensure zero mean on the target domain. All remaining effect functions are set to zero. When K=10, only the first four exposures have nonzero time-varying effects, yielding a sparse causal structure.

We repeat the simulation 100 times and evaluate FA-MFMR. Following Tian *et al.* (Tian *et al*. 2024), estimation accuracy is assessed at four interior time points (t=10,20,30,40) using the averaged squared error of the estimated effect functions. Coverage probabilities and average 95% confidence interval widths are evaluated at the same time points. Boundary regions (e.g., t=0,50) are excluded due to higher variability (Yao *et al*. 2005, Tian *et al*. 2024). Confidence intervals are constructed via bootstrap resampling within each replicate.

To benchmark FA-MFMR, we compare it with five procedures: *PACE + 2SFRI*, which predicts the functional exposures using genetic instruments and includes first-stage residuals in the outcome model (Cao *et al*. 2016); *MPCMR*, a multi-exposure extension of the continuous-time MR/GMM approach (Tian *et al*. 2024); *MFMR*, an unpenalized two-stage least-squares estimator of the concatenated FPCA scores; *pMFMR*, which applies exposure-level group SCAD without factor adjustment; and *oracle-FA-MFMR*, which uses the true simulated factor number. Further implementation details are provided in the Supplementary Materials.

We evaluate the methods across three design dimensions: sample size *n*, exposure dimension *K*, and latent-factor complexity *r*. To assess the impact of *n*, we vary n∈{500,1000,2000} while fixing K=10 and r=3. Figure 2 summarizes the distribution of the total error over 100 replicates, defined a*s*


$$\text{Total}\;\text{Error}={\left\{\sum_{k=1}^{K}\frac{1}{\left|I\right|}\sum_{t_{i}\in I}{\left({\hat{\beta }}^{\left(k\right)}(t_{i})-\beta^{\left(k\right)}(t_{i})\right)}^{2}\right\}}^{1/2},$$

where I={10,20,30,40}. Across all sample sizes, FA-MFMR and oracle-FA-MFMR achieve the lowest Total Error, followed by pMFMR, while the remaining methods show larger errors. The close agreement between FA-MFMR and oracle-FA-MFMR suggests little additional loss from data-driven factor-number selection; exposure-specific results are provided in the Supplementary Materials.

**Figure 2:**
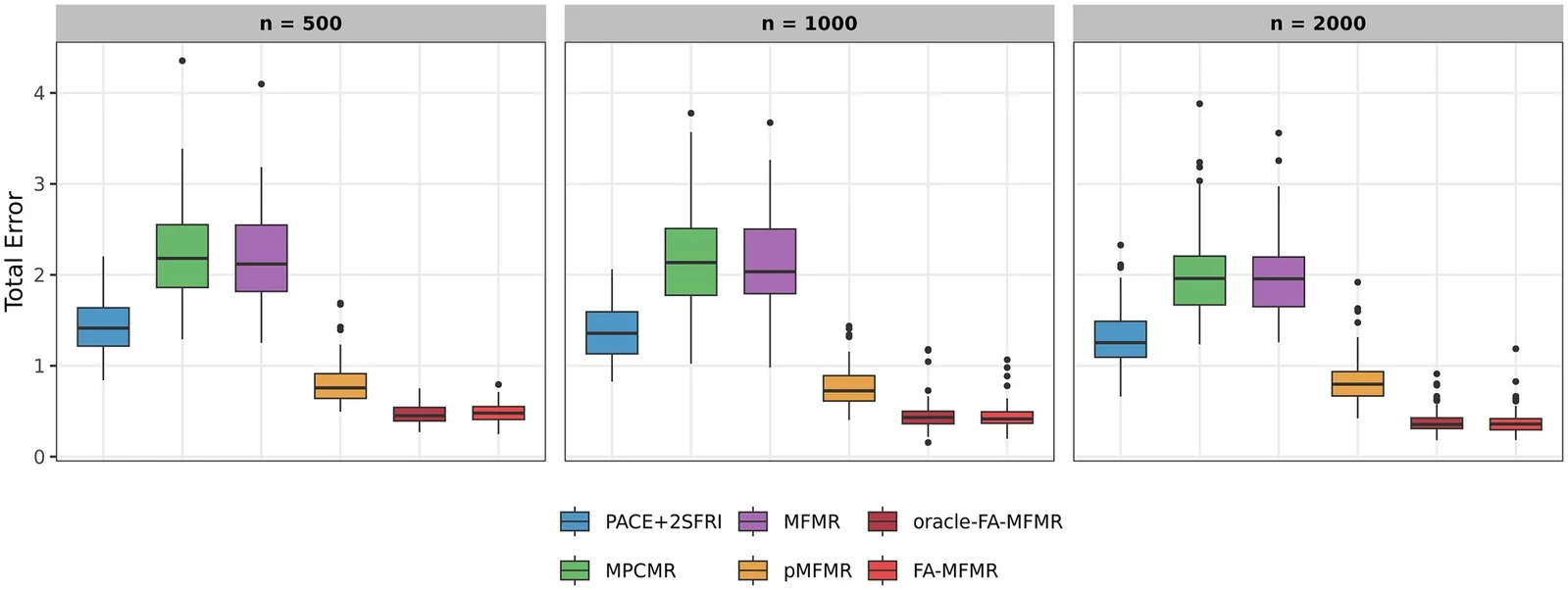
Total error of the estimated time-varying causal effect functions under the settings K=10 exposures and r=3 latent factors. Each panel corresponds to a different sample size n∈{500,1000,2000}, and each box summarizes the distribution across 100 simulation replicates.

To examine the impact of latent-factor complexity, we vary r∈{1,2,3} while fixing n=1000 and K=10 (Fig. 3). FA-MFMR and oracle-FA-MFMR consistently achieve the lowest Total Error across r=1,2,3, followed by pMFMR, while the remaining methods show larger errors; exposure-specific results are provided in the Supplementary Materials.

**Figure 3:**
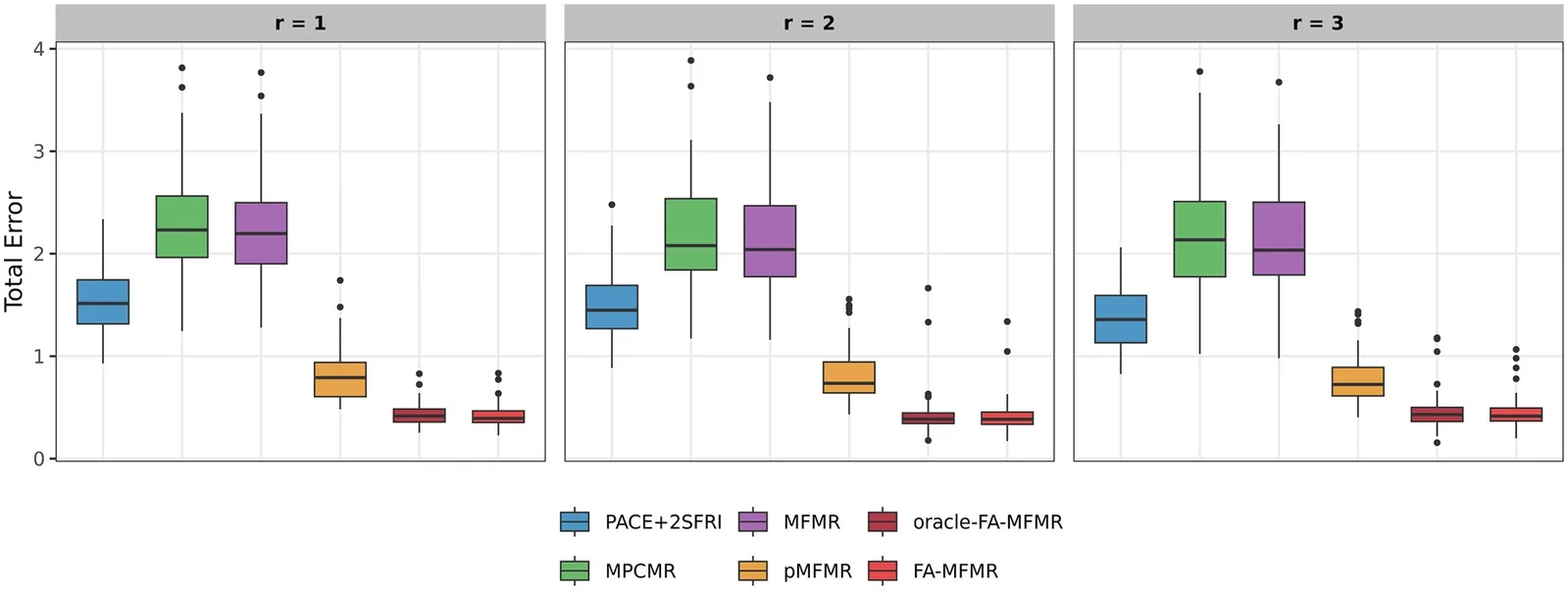
Total error of the estimated time-varying causal effect functions under the settings n=1000 and K=10 exposures. Each panel corresponds to a different latent-factor complexity r∈{1,2,3}, and each box summarizes the distribution across 100 simulation replicates.

We further evaluate the effect of exposure dimension by comparing K∈{5,10} with n=1000 and r=3 (Fig. 4). As *K* increases, FA-MFMR and oracle-FA-MFMR maintain the lowest Total Error, followed by pMFMR, whereas MFMR, MPCMR, and PACE + 2SFRI show larger errors. Exposure-specific results are reported in the Supplementary Materials.

**Figure 4:**
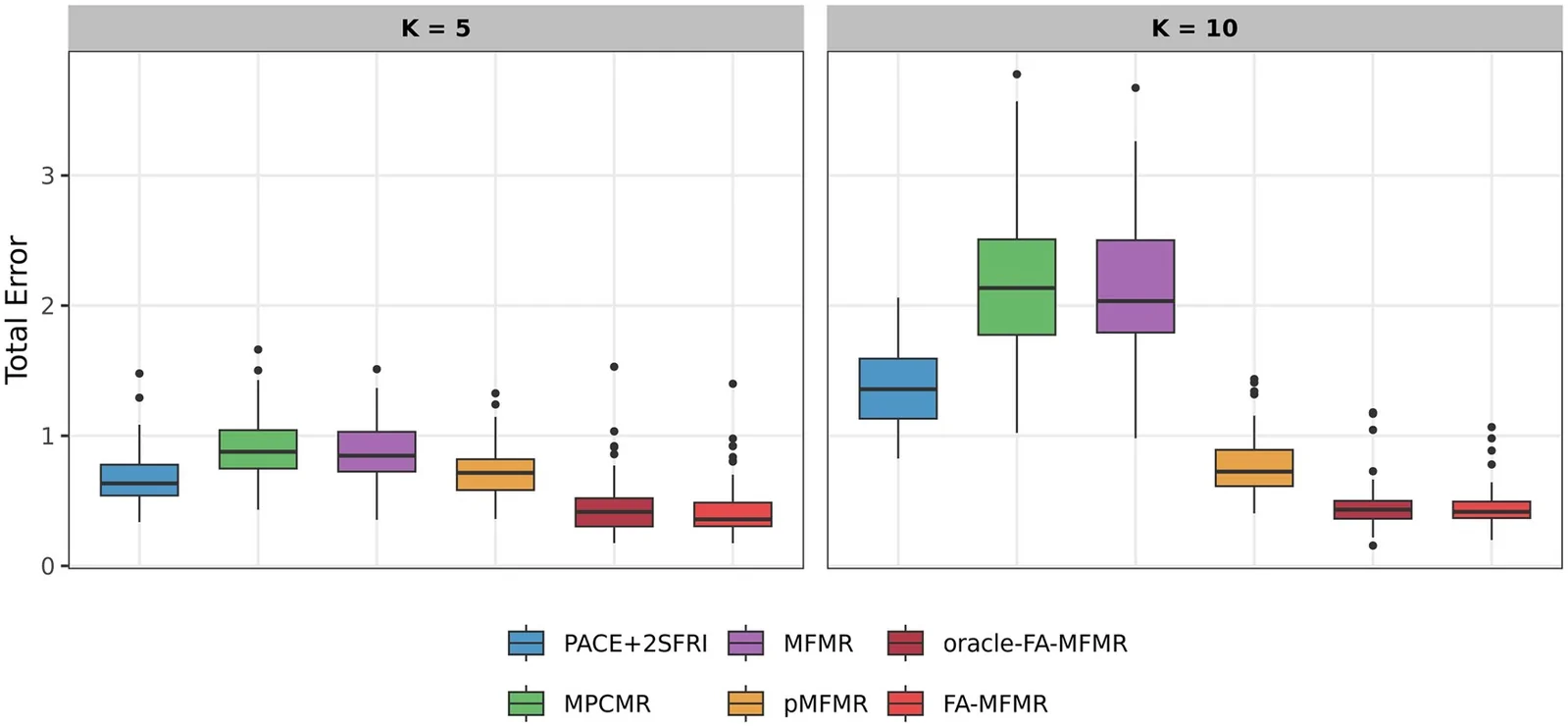
Total error of the estimated time-varying causal effect functions under different exposure dimensions, with n=1000 and r=3 latent factors. Each panel corresponds to a different number of exposures K∈{5,10}, and each box summarizes the distribution across 100 simulation replicates.

Tables 1–3 assess uncertainty quantification for FA-MFMR, MFMR, MPCMR, and PACE + 2SFRI by reporting empirical coverage and average 95% confidence interval lengths for nonzero effect functions across varying *n, r*, and *K*.

**Table 1:** Average coverage probabilities and 95% confidence interval lengths for the nonzero effect functions, averaged over t=10,20,30,40, across n∈{500,1000,2000}, with K=10, r=3, and 100 simulation replicates.

*n*		MFMR		PACE + 2SFRI		MPCMR		FA-MFMR	
		Coverage	CI length	Coverage	CI length	Coverage	CI length	Coverage	CI length
**500**	$\beta^{\left(1\right)}$	0.998	3.812	1.000	1.749	0.995	3.645	0.998	0.839
	$\beta^{\left(2\right)}$	1.000	3.626	1.000	1.731	0.995	3.468	1.000	0.852
	$\beta^{\left(3\right)}$	0.998	3.888	1.000	1.757	0.998	3.652	0.995	1.139
	$\beta^{\left(4\right)}$	0.988	3.668	0.998	1.702	0.973	3.475	0.958	0.878
**1000**	$\beta^{\left(1\right)}$	0.998	3.569	1.000	1.550	0.995	3.486	0.985	0.733
	$\beta^{\left(2\right)}$	1.000	3.581	1.000	1.641	1.000	3.489	0.993	0.795
	$\beta^{\left(3\right)}$	0.990	3.611	1.000	1.551	0.990	3.540	0.988	0.990
	$\beta^{\left(4\right)}$	0.990	3.628	0.998	1.598	0.995	3.507	0.955	0.779
**2000**	$\beta^{\left(1\right)}$	0.990	3.319	0.998	1.422	0.993	3.227	0.965	0.625
	$\beta^{\left(2\right)}$	0.995	3.237	0.993	1.577	0.993	3.175	0.985	0.753
	$\beta^{\left(3\right)}$	0.983	3.157	0.995	1.460	0.983	3.138	0.975	0.861
	$\beta^{\left(4\right)}$	1.000	3.184	0.988	1.566	0.993	3.134	0.960	0.675

**Table 2:** Average coverage probabilities and 95% confidence interval lengths for the nonzero effect functions, averaged over t=10,20,30,40, across r∈{1,2,3}, with n=2000,K=10, and 100 simulation replicates.

*r*		MFMR		PACE + 2SFRI		MPCMR		FA-MFMR	
		Coverage	CI length	Coverage	CI length	Coverage	CI length	Coverage	CI length
**1**	$\beta^{\left(1\right)}$	0.998	3.452	0.998	1.561	0.998	3.377	0.985	0.593
	$\beta^{\left(2\right)}$	0.990	3.439	1.000	1.674	0.990	3.359	0.985	0.704
	$\beta^{\left(3\right)}$	0.978	3.483	0.998	1.585	0.983	3.404	0.975	0.765
	$\beta^{\left(4\right)}$	0.975	3.423	0.995	1.644	0.985	3.345	0.960	0.686
**2**	$\beta^{\left(1\right)}$	0.990	3.528	0.995	1.483	0.995	3.400	0.978	0.598
	$\beta^{\left(2\right)}$	0.993	3.463	0.995	1.662	0.998	3.390	0.985	0.740
	$\beta^{\left(3\right)}$	0.983	3.514	0.995	1.512	0.988	3.426	0.975	0.831
	$\beta^{\left(4\right)}$	0.990	3.535	0.995	1.556	0.993	3.440	0.958	0.687
**3**	$\beta^{\left(1\right)}$	0.990	3.319	0.998	1.422	0.993	3.227	0.965	0.625
	$\beta^{\left(2\right)}$	0.995	3.237	0.993	1.577	0.993	3.175	0.985	0.753
	$\beta^{\left(3\right)}$	0.983	3.157	0.995	1.460	0.983	3.138	0.975	0.861
	$\beta^{\left(4\right)}$	1.000	3.184	0.988	1.566	0.993	3.134	0.960	0.675

**Table 3:** Average coverage probabilities and 95% confidence interval lengths for the nonzero effect functions, averaged over t=10,20,30,40, across K∈{5,10}, with n=2000,r=3, and 100 simulation replicates.

*K*		MFMR		PACE + 2SFRI		MPCMR		FA-MFMR	
		Coverage	CI length	Coverage	CI length	Coverage	CI length	Coverage	CI length
**5**	$\beta^{\left(1\right)}$	0.968	1.448	0.990	0.901	0.973	1.421	0.998	0.974
	$\beta^{\left(2\right)}$	0.973	1.453	0.983	1.050	0.965	1.426	0.990	0.979
	$\beta^{\left(3\right)}$	0.920	1.451	0.983	0.914	0.912	1.429	0.988	1.052
	$\beta^{\left(4\right)}$	0.963	1.452	0.973	0.997	0.955	1.429	0.990	0.904
**10**	$\beta^{\left(1\right)}$	0.990	3.319	0.998	1.422	0.993	3.227	0.965	0.625
	$\beta^{\left(2\right)}$	0.995	3.237	0.993	1.577	0.993	3.175	0.985	0.753
	$\beta^{\left(3\right)}$	0.983	3.157	0.995	1.460	0.983	3.138	0.975	0.861
	$\beta^{\left(4\right)}$	1.000	3.184	0.988	1.566	0.993	3.134	0.960	0.675

Across these settings, FA-MFMR attains near-nominal coverage with substantially shorter confidence intervals. MFMR and MPCMR show broadly comparable coverage but wider intervals, whereas PACE + 2SFRI tends to over-cover with inflated interval lengths. These patterns remain stable across *n, r*, and *K*, indicating more efficient uncertainty quantification with FA-MFMR.

We further evaluate exposure-level support recovery using sensitivity, specificity, and false discovery rate (FDR). Let $S_{0}=\left\{k:{\left\|\beta^{\left(k\right)}\right\|}_{L_{2}}>0\right\}$ denote the true set of active exposures and $\hat{S}$ the estimated active set. Sensitivity is defined as TP/(TP + FN), specificity as TN/(TN + FP), and FDR as FP/max(TP + FP, 1). Because PACE + 2SFRI, MPCMR, and unpenalized MFMR do not explicitly perform sparse exposure selection, support-recovery metrics are reported for pMFMR, oracle-FA-MFMR, and FA-MFMR.

Figure 5 shows that all three penalized methods achieve high specificity and low FDR across the considered settings. FA-MFMR and oracle-FA-MFMR generally attain sensitivity comparable to or slightly higher than pMFMR, with sensitivity approaching one as the sample size increases. The support-recovery performance is also stable across r=1,2,3, and FA-MFMR closely tracks the oracle-factor benchmark. Together with the lower estimation errors in Figs 2–4, these results indicate that factor augmentation improves estimation accuracy while retaining accurate exposure-level support recovery.

**Figure 5:**
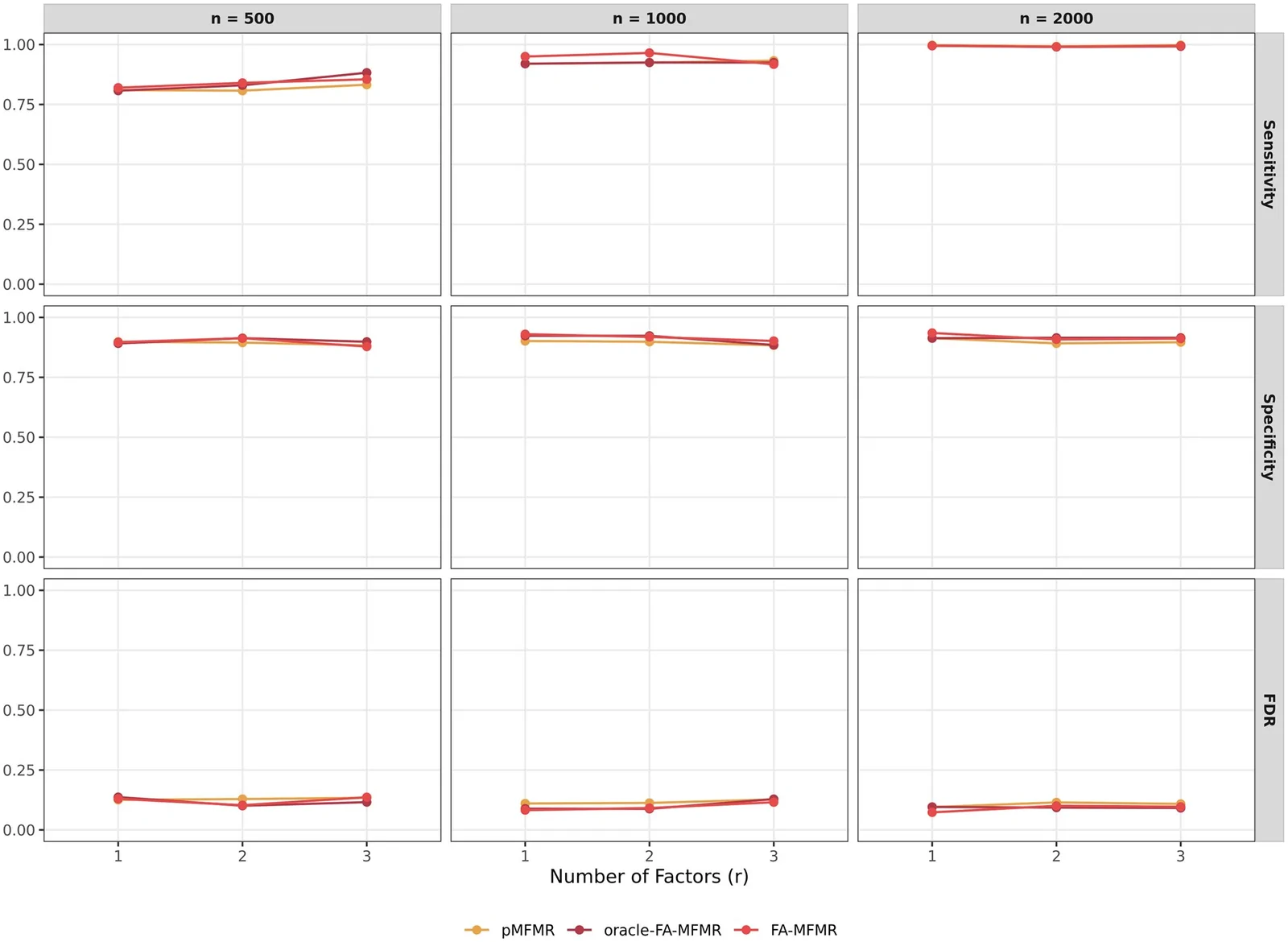
Exposure-level support recovery under varying latent-factor complexity and sample sizes.

Additional simulations examine a range of departures from the baseline design, including alternative factor structures, instrument and first-stage perturbations, informative visit patterns, factor-number misspecification, and a PPMI-calibrated setting. FA-MFMR retains favorable performance in most settings, with reduced advantages under horizontal pleiotropy and when the causal signal is concentrated in the common-factor space; detailed results are provided in Supplementary Materials.

### 3.2 Analysis of Parkinson’s disease progression in the PPMI cohort

We apply FA-MFMR to the Parkinson’s Progression Markers Initiative (PPMI), a multi-center longitudinal cohort of PD patients and controls (Marek, 2011, 2018). Although the cohort spans a broad age range, observations are concentrated between ages 50 and 80 (Fig. 6), which defines the functional domain in our analysis. Chronological age serves as the functional time index in FA-MFMR. This differs from follow-up time, which denotes elapsed time since study entry, and from disease duration, which, where applicable, denotes elapsed time since PD onset or diagnosis. Neither follow-up time nor disease duration serves as the functional time index in the primary analysis.

**Figure 6:**
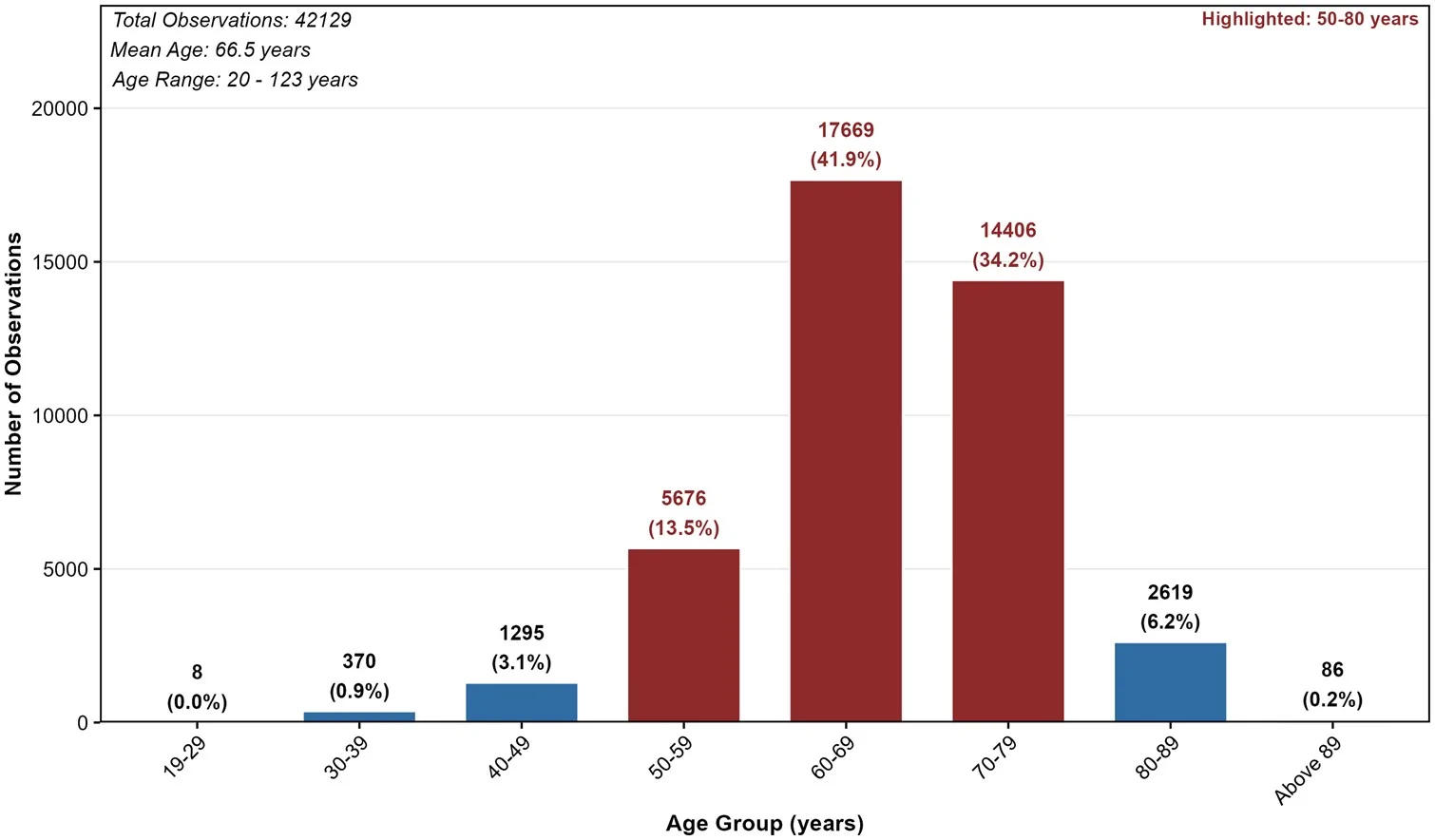
Empirical distribution of all recorded measurement ages in the full PPMI dataset.

We analyze 13 longitudinal biomarkers covering metabolic, inflammatory, cardiovascular, autonomic, and physiological domains: serum uric acid, glucose, albumin, white blood cell count, neutrophils, lymphocytes, monocytes, eosinophils, basophils, supine systolic and diastolic blood pressure, supine heart rate, and BMI (Havelund *et al*. 2017, Seifar *et al*. 2022, Hepp *et al*. 2023, Qu *et al*. 2023, Li *et al*. 2024). Each biomarker typically has 5–13 observations within the first 4–5 years of follow-up. Consistent with this follow-up structure, the median within-person chronological-age span of the repeated biomarker measurements in the final analytic cohort is 5.1 years (IQR, 4.1–6.1 years), with a median of six distinct measurement ages per participant. Thus, the 50–80-year functional domain does not represent 30 years of within-person follow-up. Instead, it is supported by staggered longitudinal segments contributed by participants observed at different chronological ages and reconstructed through the sparse functional representation. Detailed age-coverage diagnostics are provided in Supplementary Materials.

The outcome is the MDS-UPDRS Part III motor score (NP3TOT), a standard measure of motor impairment in PD (Goetz *et al*. 2008, 2009). For each participant, we use the available NP3TOT assessment closest to age 80 as the subject-specific terminal outcome, observed at chronological age $T_{i}$. Only biomarker measurements obtained before $T_{i}$ are used as observed longitudinal exposure data for participant *i*. Thus, unlike the common terminal time assumed in the theoretical formulation, the PPMI application uses subject-specific terminal outcome ages. Because sparse functional reconstruction represents the latent biomarker trajectories over the common age domain of 50–80 years, reconstructed trajectory segments for $t>T_{i}$ are model-based continuations informed by the participant’s available pre-outcome measurements and the estimated population-level functional structure. These reconstructed segments are not interpreted as observed pre-outcome exposure histories and are not assigned a subject-specific temporal causal interpretation. After restricting to participants aged 50–80 with an available NP3TOT assessment under this outcome definition, 1,450 individuals remain. In this final analytic cohort, the selected terminal NP3TOT ages have a median of 71.8 years (IQR, 66.0–76.9 years) and range from 51.0 to 81.4 years.

Instrumental variables are derived from quality-controlled PPMI whole-genome sequencing data. Candidate instruments are selected from external GWAS to avoid winner’s curse (Zöllner and Pritchard 2007), LD- clumped, mapped to PPMI, and tested against the corresponding biomarker at visit V08 (*P* < 0.05), yielding 231 SNPs across 13 biomarkers. After aligning subjects with complete biomarker trajectories, NP3TOT outcomes, and genotype data, the final analytic dataset includes 703 individuals. According to the PPMI enrollment categories, these participants comprise 345 participants in the PD group, 68 in the genetic PD group, 151 healthy controls, 52 prodromal participants, 48 genetically unaffected participants, and 39 participants with SWEDD. These enrollment groups are combined in the primary analysis. To evaluate the sensitivity of the results to cohort composition, we additionally repeat FA-MFMR after restricting the analysis to the 413 participants enrolled in the PD or genetic PD groups. We further conduct an age-70 landmark analysis in which only biomarker measurements obtained before age 70 contribute to the exposure trajectories and the selected terminal NP3TOT assessment is required to occur after age 70. This landmark design provides a stricter temporal-ordering sensitivity analysis by ensuring that the analyzed exposure domain precedes the outcome assessment for every included participant. Detailed procedures are provided in the Supplementary Materials, available as supplementary data at *Bioinformatics* online.

The analytic dataset contains correlated longitudinal exposures, irregular observation times, and high-dimensional genetic instruments. Figures 7 and 8 summarize the data-processing workflow and the sparse, heterogeneous structure of the final analytic dataset.

**Figure 7:**
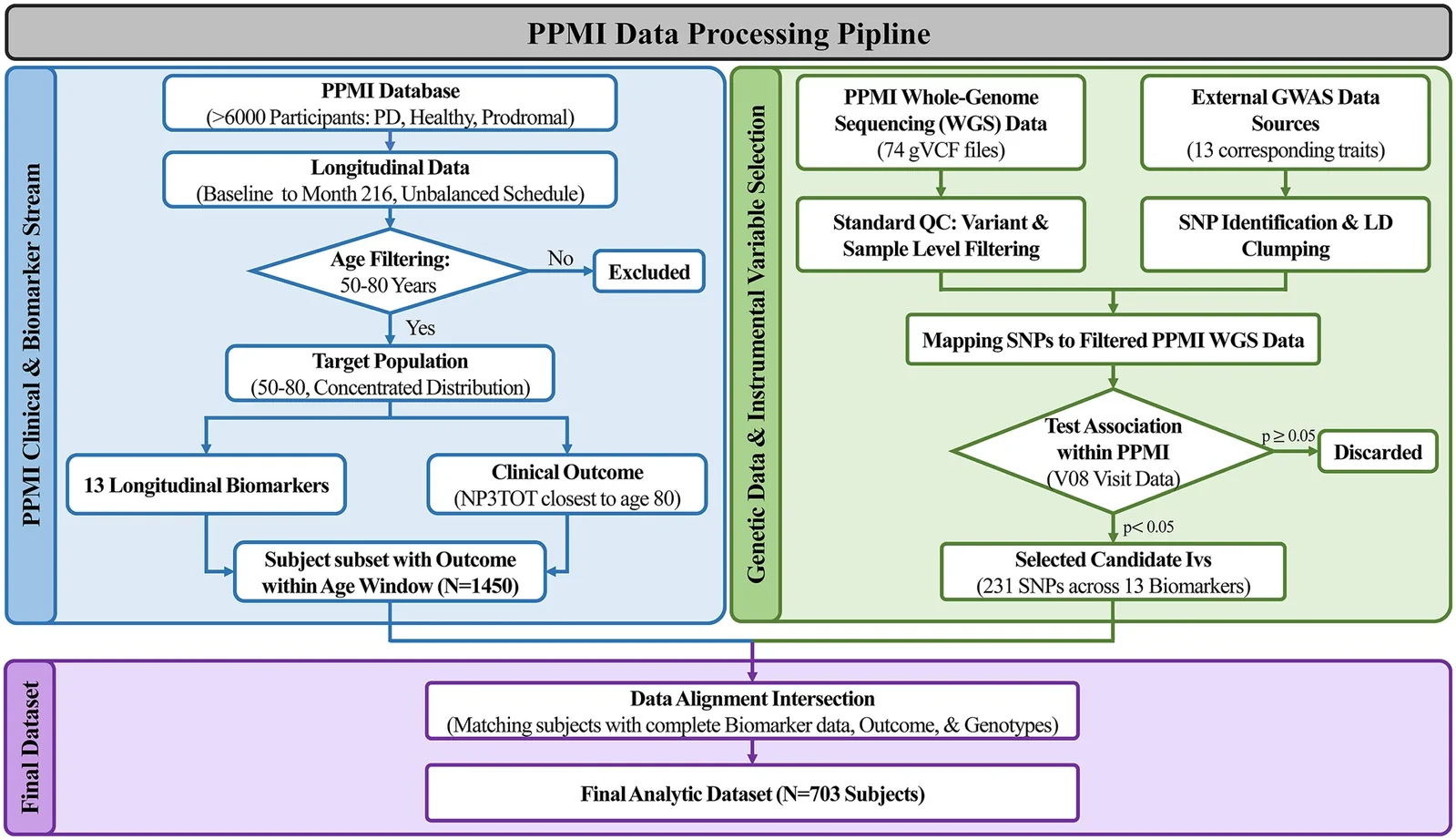
Overview of the PPMI data processing pipeline from raw measurements and genomic data to the final analytic cohort.

**Figure 8:**
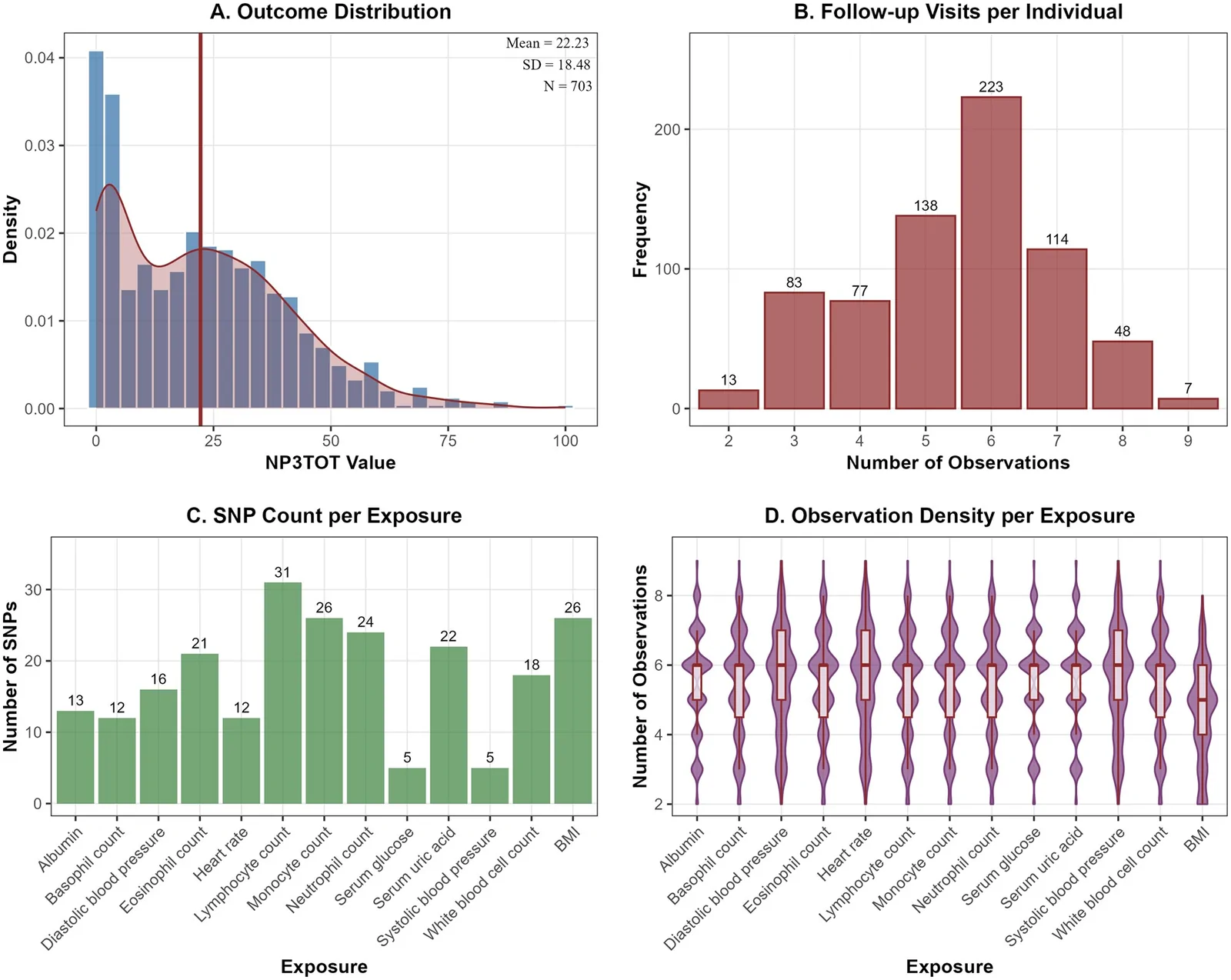
PPMI analytic-data summary: (A) NP3TOT distribution; (B) follow-up visits per subject; (C) retained SNPs per biomarker; and (D) longitudinal observation density by biomarker.

We estimate exposure-specific time-varying coefficient functions using FA-MFMR to characterize age-dependent patterns in the effects of the biomarker trajectories on NP3TOT. Unlike the cross-sectional MR, FA-MFMR allows causal effects to vary smoothly over ages 50–80, enabling detection of age-dependent patterns. To assess robustness to potentially influential or invalid instruments, we additionally examine covariate-adjusted SNP–NP3TOT associations and instrument-level heterogeneity, perform leave-one-SNP and leave-one-biomarker analyses, and conduct an exploratory scalar multivariable MR analysis at visit V08. Because conditional instrument strength is very weak in the scalar analysis, its results are considered inconclusive and are not used to validate the longitudinal FA-MFMR findings. Detailed procedures are provided in the Supplementary Materials, available as supplementary data at *Bioinformatics* online.

Figure 9 shows the estimated time-varying effects for all 13 biomarkers with pointwise 95% bootstrap confidence bands based on B=500 participant-level bootstrap resamples. The bootstrap refits rerun the major data-adaptive steps of the FA-MFMR pipeline, including PACE/FPCA estimation and truncation, IV projection, latent-factor estimation and factor number selection, and SCAD tuning and coefficient estimation. We further construct joint 95% simultaneous confidence bands over all 13 biomarkers and the full age domain and perform biomarker-level global curve tests with joint max- *T* multiplicity adjustment. Detailed procedures and results are provided in Supplementary Materials. The estimated curves vary substantially in shape and magnitude. Neutrophils show an upward trend, white blood cell count shows pronounced declines, and several other biomarkers remain near zero over much of the age range; some patterns are consistent with previous findings (Bartl *et al*. 2023, Hepp *et al*. 2023, Koros *et al*. 2023, Pike *et al*. 2024, He *et al*. 2025).

**Figure 9:**
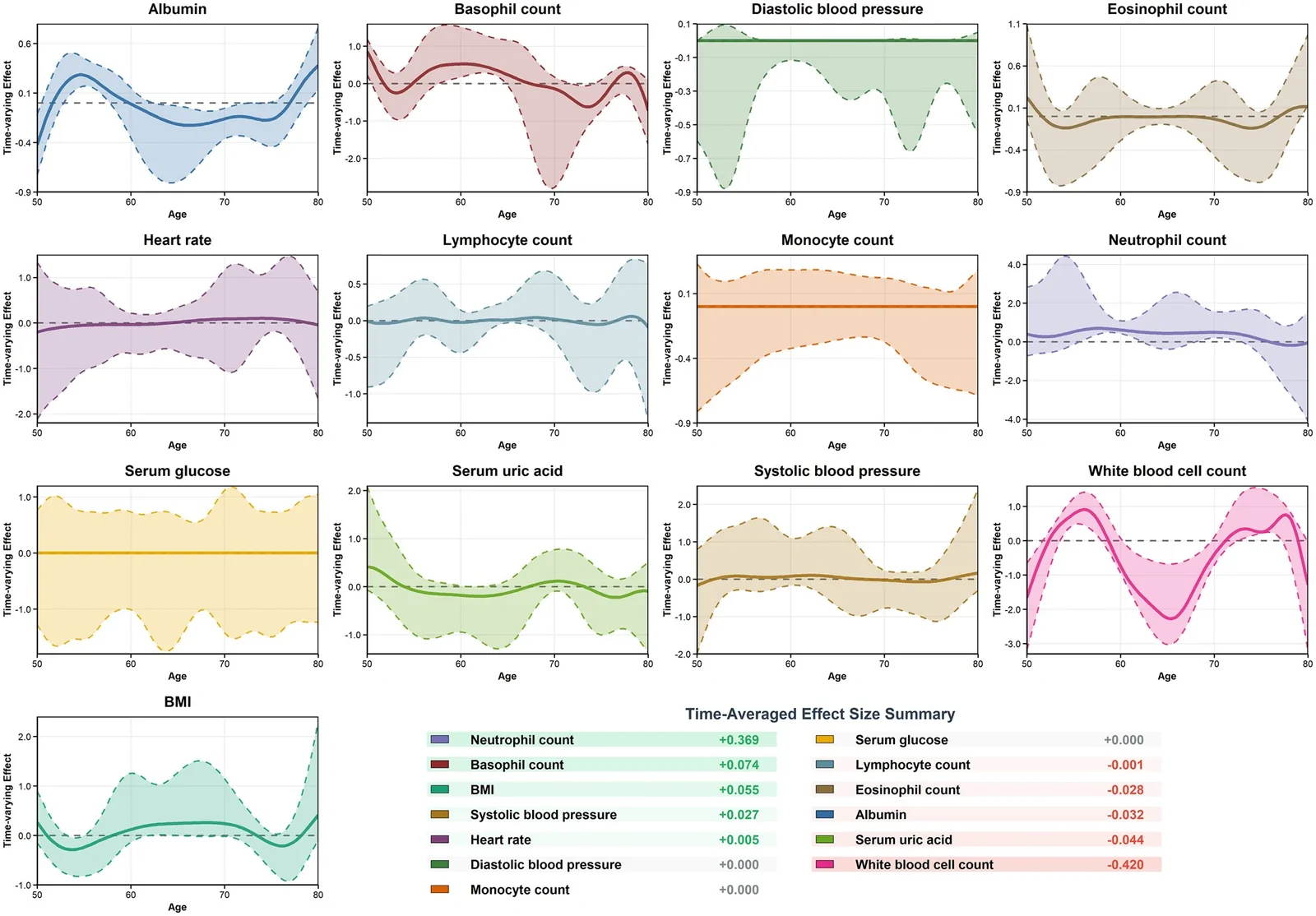
Estimated time-varying trajectory-level causal coefficient functions for all thirteen biomarkers over ages 50–80. Each panel displays the estimated time-varying causal effect curve for one biomarker, accompanied by pointwise 95% bootstrap confidence bands. The summary panel reports the time-averaged effect size for each biomarker, providing an overall ranking of their cumulative influence across the 50–80 age window.

At the pointwise level, the confidence bands show localized departures from zero for albumin, basophils, neutrophils, and white blood cell count. Specifically, the pointwise bands exclude zero for albumin around ages 53–56 and 62–72, basophils around ages 56–65, neutrophils around ages 55–62 and 68–72, and white blood cell count around ages 54–56 and 60–68. However, these pointwise patterns do not account for multiplicity across biomarkers and the continuous age domain. In the joint analysis, the 95% simultaneous confidence bands include zero throughout ages 50–80 for all 13 biomarkers, and none of the biomarker-level global curve tests reaches statistical significance after joint max-*T* adjustment (all adjusted P≥0.855). We therefore interpret the localized departures identified by the pointwise analysis as exploratory age-dependent patterns rather than as confirmatory significant age windows.

To complement the pointwise results, we summarize each biomarker’s overall contribution by integrating its estimated effect over ages 50–80. Figure 10A shows descriptive variation between positive and negative cumulative effect estimates. Neutrophils and white blood cell count show the largest positive and negative integrated estimates, respectively.

**Figure 10:**
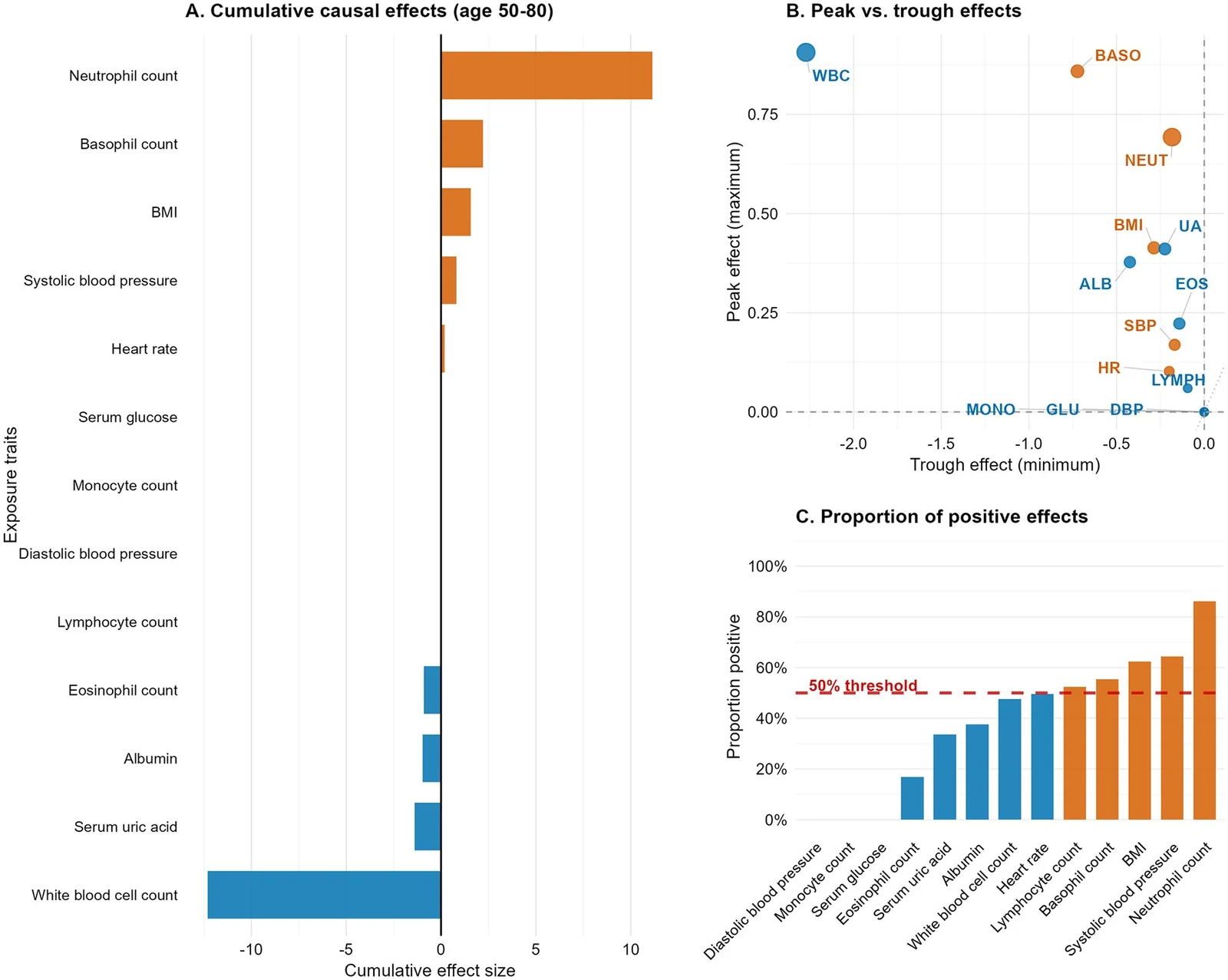
Cumulative and distributional summaries of time-varying causal effects over ages 50–80. (A) Cumulative causal effects obtained by integrating each biomarker’s estimated effect curve across age 50–80. (B) Scatter plot of peak versus trough effect values for all biomarkers, summarizing the range of age-specific variation. (C) Proportion of ages at which each biomarker’s estimated effect is positive, indicating directional persistence over the analyzed age range of 50–80 years.

Because cumulative effects may mask temporal variation, we also examine the maximum and minimum values of each estimated curve (Fig. 10B). Basophils, neutrophils, BMI, serum uric acid, and white blood cell count show the widest age-dependent ranges.

We further report the proportion of ages with positive estimated effects (Fig. 10C), which aligns with the cumulative patterns. Neutrophils show the highest proportion of positive estimates, whereas several predominantly negative biomarkers remain below 50%.

To quantify uncertainty in these curve-level summaries, we additionally calculate 95% bootstrap confidence intervals for the integrated effect, the maximum and minimum effects, the ages at which the extrema occur, and the proportion of ages with positive estimated effects. The confidence intervals for the integrated effects include zero for all 13 biomarkers, while the intervals for the extrema, their locations, and the positive-age proportions are generally broad. In particular, the bootstrap intervals for the locations of the extrema frequently span a substantial part of the 50–80-year age domain. These results indicate considerable uncertainty in the scalar curve summaries and support their interpretation primarily as descriptive features of the estimated coefficient functions. Full results are reported in Supplementary Materials.

Given the instrument-validity assumptions underlying MR and the sensitivity of selected coefficient functions to individual instruments and the composition of the multivariable biomarker set, these results are interpreted as age-localized patterns in the estimated trajectory-level coefficient functions rather than as independently identified causal effects at individual ages.

## 4 Discussion

In this study, we propose FA-MFMR for multiple correlated longitudinal exposures and a terminal outcome. FA-MFMR integrates ideas from longitudinal and multivariable MR to jointly model multiple correlated longitudinal exposures. It combines functional representation with factor augmentation to capture shared variation while identifying exposure-specific coefficient functions. Simulations show that factor augmentation improves estimation accuracy and yields more efficient uncertainty quantification, while maintaining accurate exposure-level support recovery across varying sample sizes and latent complexity.

In the PPMI analysis, FA-MFMR identifies age-localized pointwise patterns in the estimated trajectory-level causal coefficient functions for albumin, basophils, neutrophils, and white blood cell count, highlighting temporal heterogeneity in the fitted functional effects. However, the joint simultaneous confidence bands and multiplicity-adjusted global curve tests do not provide confirmatory curve-level evidence for any of the 13 biomarkers. We therefore interpret these localized patterns as exploratory and hypothesis-generating. The cumulative and extrema-based summaries provide complementary descriptions of the estimated curves, but their bootstrap intervals indicate substantial uncertainty. These patterns reflect the temporal weighting of instrument-supported trajectory variation and should not be interpreted as independently identified interventions at individual ages. The landmark and PD-only sensitivity analyses show that the degree of departure from the primary coefficient-function system varies across biomarkers, with the largest discrepancies concentrated in white blood cell count and neutrophil count. The divergent patterns of total white blood cell count and neutrophil count may reflect both their distinct biological meanings and their strong dependence within the multivariable system. Total white blood cell count aggregates several leukocyte populations, whereas neutrophils represent a major innate immune component whose phenotype and function undergo age-related remodeling; such immune changes may be particularly relevant in the context of aging and PD. Because these correlated blood-cell measures are modeled jointly, their coefficient functions represent conditional rather than marginal patterns, which may also contribute to the observed directional differences. Intercurrent infection and time-varying medication use are not explicitly modeled and may also contribute to variation in peripheral blood-cell measures during follow-up.

Several limitations warrant future work. First, causal validity depends on instrument strength and validity. Factor augmentation is designed to account for dependence among longitudinal exposure trajectories and does not itself address horizontal pleiotropy or protect against violations of the exclusion restriction. Thus, weak instruments and residual pleiotropy may persist despite external GWAS-based instrument selection. FPCA truncation may further exacerbate weak-instrument bias if retained components have weak genetic associations, motivating careful component selection and more robust estimators (Hansen *et al*. 1996, Seng *et al*. 2023, Ye *et al*. 2024, Zhang and Sun 2025). Second, although the IV formulation allows unmeasured confounding processes to vary over time under the stated instrument assumptions, more complex longitudinal mechanisms involving evolving disease states, treatment-related feedback, or attrition may require additional assumptions. Third, although we supplement the pointwise bands with joint simultaneous confidence bands and multiplicity-adjusted global curve tests, the resulting inference remains bootstrap-based and selection-aware rather than exact conditional post-selection inference. The wide simultaneous bands highlight the finite-sample uncertainty associated with the data-adaptive functional and penalized estimation steps. Developing more powerful formal selective-inference procedures for multivariable functional MR remains an important direction for future work. Fourth, the framework assumes linear SNP-exposure relationships, which may be restrictive; nonlinear first-stage models are a promising extension (Barber *et al*. 2017). Moreover, extending FA-MFMR to summary-data settings would broaden applicability. Finally, replication in independent cohorts is needed to confirm the observed age-indexed patterns in the estimated trajectory-level coefficient functions.

## Supplementary Material

btag725_Supplementary_Data

## Data Availability

The data that support the findings of this study are available from PPMI. Restrictions apply to the availability of these data, which were used underlicense for this study. Data are available from https://www.ppmi-info.org/ with the permission of PPMI. The R codes used for the simulations and real analysis in this article are available from https://github.com/YQHuFD/FA-MFMR.
