# Supplementary material for: FA-MFMR: a multivariable functional Mendelian randomization method that accounts for correlated longitudinal exposures via factor augmentation
Source: Bioinformatics. 2026 Oct 8;42(10):btag725. doi: 10.1093/bioinformatics/btag725 (PMC13658255; doi:10.1093/bioinformatics/btag725)
Supplement: Supplementary file 1 [file btag725_supplementary_data.zip › supplemental material.pdf]

Supplementary Materials for "FA-MFMR: a multivariable functional Mendelian randomization method that accounts for correlated longitudinal exposures via factor augmentation"

Dong Chen[1], Junrong Deng[1], Siyuan Shen[1], Yuyang Zhou[1], Hanyu Cui[1], Yu Qiu[1], Qiming Li[1], and Yue-Qing Hu[1,2,*]

[1]State Key Laboratory of Genetics and Development of Complex Phenotypes, Institute of Biostatistics, School of Life Sciences, Fudan University, Shanghai, China

[2]Shanghai Center for Mathematical Sciences, Fudan University, Shanghai, China

[*]*email*: yuehu@fudan.edu.cn

Contents

S1: Assumptions and Theoretical Properties of FA-MFMR . . . 3

S2: Additional Results . . . 10

S2.1: Exposure-specific estimation errors under varying sample sizes . . . 10

S2.2: Exposure-specific estimation errors under different latent-factor complexities . . . 13

S2.3: Exposure-specific estimation errors under varying exposure dimensions . . . 16

S2.4: Sensitivity to the ordering of time-varying effect functions under varying exposure dimensions . . . 19

S2.5: Sensitivity to causal-signal alignment with the common-factor space . . . 19

S2.6: Pleiotropy and influence sensitivity analyses in the PPMI cohort . . . 22

1. Direct SNP–outcome associations and instrument-level heterogeneity . . . 22
2. Leave-one-SNP analysis . . . 23
3. Leave-one-biomarker analysis . . . 23
4. Complementary pleiotropy-robust multivariable MR analysis . . . 25

S2.7: Longitudinal age coverage and cohort sensitivity analyses 28
1. Within-participant age coverage, local cross-subject support, and terminal outcome ages 28
2. Landmark and PD-only sensitivity analyses 29
S2.8: Additional simulation settings beyond the baseline design 33
1. Departures from the exposure and factor structure 33
2. Departures from the instrument and first-stage assumptions 35
3. Informative visit patterns 37
4. PPMI-calibrated simulation 39
5. Sensitivity to factor-number misspecification 41
S2.9: Multiplicity-aware bootstrap uncertainty for PPMI coefficient functions and curve summaries 42
1. Joint simultaneous confidence bands 42
2. Global curve tests 43
3. Uncertainty for curve summaries 45
S2.10: Sensitivity to instrument-selection criteria 47
1. Simulation sensitivity to instrument-selection stringency 47
2. PPMI sensitivity to the internal instrument-selection threshold 48

S3: Implementation details of competing methods **53**

S1: Assumptions and Theoretical Properties of FA-MFMR

In this subsection, we first clarify identification of the trajectory-level causal coefficient functions in the retained FPCA representation, and then state the regularity conditions required for the large-sample properties of the FA-MFMR estimator (Kong et al., 2016; Ma et al., 2025).

Assumption 1. (Exogeneity) $\mathbb{E}[Z_i\tilde{\varepsilon}_i] = 0$.

Assumption 2. (Relevance) The matrix $\Gamma_{Za} = \mathbb{E}[Z_i\boldsymbol{a}_i^\top]$ has full column rank p, and the smallest singular value is bounded away from zero.

Assumption 3. The number of instruments J satisfies $J \geq p$ and $\log J = o(n)$.

Proposition 1. (Identification in the retained FPCA representation) Under **Assumptions 1–3**, the coefficient vector $\boldsymbol{H}$ in equation (3) of the main text is uniquely identified through

$$\mathbb{E}\left[Z_iY_i\right] = \Gamma_{Za}\boldsymbol{H}.$$

If Γ_{Za} is rank deficient, only linear contrasts $\boldsymbol{c}^\top\boldsymbol{H}$ with $\boldsymbol{c} \in \operatorname{row}\left(\Gamma_{Za}\right)$ are identifiable, whereas directions in $\operatorname{null}\left(\Gamma_{Za}\right)$ cannot be separately recovered from the available instruments.

Proof. From equation (3) of the main text,

$$Y_i = \boldsymbol{H}^\top\boldsymbol{a}_i + \tilde{\varepsilon}_i.$$

Therefore,

$$\mathbb{E}\left[Z_iY_i\right] = \mathbb{E}\left[Z_i\boldsymbol{a}_i^\top\right]\boldsymbol{H} + \mathbb{E}\left[Z_i\tilde{\varepsilon}_i\right] = \Gamma_{Za}\boldsymbol{H},$$

where the last equality follows from **Assumption 1**. Under **Assumption 2**, Γ_{Za} has full column rank, so $\boldsymbol{H}$ is uniquely identified. If Γ_{Za} is rank deficient, any $\boldsymbol{d} \in \operatorname{null}\left(\Gamma_{Za}\right)$ satisfies $\Gamma_{Za}(\boldsymbol{H}+\boldsymbol{d}) = \Gamma_{Za}\boldsymbol{H}$, and hence only contrasts whose coefficient vectors lie in $\operatorname{row}\left(\Gamma_{Za}\right)$ are identifiable.

Remark 1. **Proposition 1** shows that recoverability is restricted at two levels. First, FPCA truncation restricts the analysis to trajectory directions contained in the retained spaces $\mathcal{M}_k = \operatorname{span}\left\{\gamma_1^{(k)}, \ldots, \gamma_{m_k}^{(k)}\right\}$. Second, within these retained spaces, the available genetic instruments identify only score directions supported by Γ_{Za}. Thus, functional directions omitted by FPCA truncation are not recovered, and retained directions unsupported by sufficient instrument-induced variation are not separately identifiable. At the sample level, $\widehat{\boldsymbol{A}} = P_{\boldsymbol{Z}}\boldsymbol{A}$ extracts the instrument-predictable component of the retained FPCA score matrix and is the sample analogue

of this instrument-supported variation.

Assumption 4. For $k = 1, \ldots, K$, $\tau_1^{(1)} < \infty$, $\tau_r^{(k)} - \tau_{r+1}^{(k)} \geq Cr^{-a-1}$ for $k \geq 1$, where $\tau_r^{(k)}$ is the r-th eigenvalue of covariance functions $\mathrm{Cov}\left(X_i^{(k)}(s), X_i^{(k)}(t)\right)$.

Assumption 5. Assume for $k = 1, \ldots, K$, we have $m_k \leq s_n$, and $(s_n^{2a+2} + s_n^{(a+4)})/n = o(1)$.

Assumption 6. For $k = 1, \ldots, K$, for any $C > 0$ there exist an $\epsilon > 0$ such that

$$\sup_{t \in [0,T]} \left[\mathbb{E}\left\{ \left| X^{(k)}(t) \right|^C \right\} \right] < \infty, \quad \sup_{s,t \in [0,T]} \left(\mathbb{E}\left[\left\{ |s-t|^{-\epsilon} \left| X^{(k)}(s) - X^{(k)}(t) \right| \right\}^C \right] \right) < \infty.$$

For each integer $q \geq 1$, $\left(\tau_r^{(k)}\right)^{-q} \mathbb{E}[a_r^{(k)}]^{2q}$ is bounded uniformly in r.

Assumption 7. For $k = 1, \ldots, K$, $X^{(k)}(t)$ is twice continuously differentiable on $[0, T]$ with probability 1, and $\int \mathbb{E}\left\{X^{(k)\prime\prime}(t)\right\}^4 dt < \infty$, where $X^{(k)\prime\prime}(\cdot)$ denotes the second derivative of $X^{(k)}(\cdot)$.

Assumption 8. For $k = 1, \ldots, K$, the observation times for exposure k of individual i, $\left\{t_{il}^{(k)}, l = 1, \ldots, n_i^{(k)}\right\}$ are considered deterministic and ordered increasingly for $i = 1, \ldots, n$. There exist densities $p_i^{(k)}$ uniformly smooth over $[0, T]$, satisfying $\int_{[0,T]} p_i^{(k)}(t)dt = 1$ and $0 < c_1 < \inf_i \left\{\inf_{t \in [0,T]} p_i^{(k)}(t)\right\} < \sup_i \left\{\sup_{t \in [0,T]} p_i^{(k)}(t)\right\} < c_2 < \infty$ that generate $t_{il}^{(k)}$ according to $t_{il}^{(k)} = P_i^{(k)-1}\left\{l/\left(n_i^{(k)} + 1\right)\right\}$, where $P_i^{(k)-1}$ is the inverse of $P_i^{(k)}(t) = \int_{-\infty}^t p_i^{(k)}(s)ds$. For each $k = 1, \ldots, K$, there exist a common sequence of bandwidths $h^{(k)}$ such that $0 < c_1 < \inf_i h_i^{(k)}/h^{(k)} < \sup_i h_i^{(k)}/h^{(k)} < c_2 < \infty$, where $h_i^{(k)}$ is the bandwidth for $X_i^{(k)}$. The kernel density function is smooth and compactly supported.

Assumption 9. Let $t_{i0}^{(k)} = 0, t_{i,n_i^{(k)}+1}^{(k)} = T$, let $\Delta_i^{(k)} = \sup\left\{t_{i,l+1}^{(k)} - t_{i,l}^{(k)}, l = 0, \ldots, n_i^{(k)}\right\}$ and $n^{(k)} = n^{(k)}(n) = \inf_{i=1,\ldots,n} n_i^{(k)}$. Denoting $0 < \lim a_n/b_n < \infty$ by $a_n \sim b_n$, assume for $k = 1, \ldots, K$, $\sup_i \Delta_i^{(k)} = O((n^{(k)})^{-1})$, $h^{(k)} \sim (n^{(k)})^{-1/5}$, $(n^{(k)})n^{-5/4} \to \infty$.

Assumption 10. Let $\boldsymbol{\gamma} = \widehat{\boldsymbol{\Lambda}}^\top \boldsymbol{H} \in \mathbb{R}^r$ denote the unpenalized nuisance coefficient associated with the estimated common-factor component, and let

$$L_n\left(\boldsymbol{Y}, \widehat{\boldsymbol{W}}\boldsymbol{\theta}\right) = \frac{1}{2n}\sum_{i=1}^n \left[Y_i - \left(\widehat{\boldsymbol{f}}_i^\top \boldsymbol{\gamma} + \widehat{\boldsymbol{u}}_i^\top \boldsymbol{H}\right)\right]^2 + \sum_{k=1}^K \sum_{j=1}^{m_k} p_\lambda^{\mathrm{SCAD}}(\eta_j^{(k)}),$$

where $\widehat{\boldsymbol{w}}_i = \left(\widehat{\boldsymbol{u}}_i^\top, \widehat{\boldsymbol{f}}_i^\top\right)^\top$, $\widehat{\boldsymbol{W}} = (\widehat{\boldsymbol{w}}_1, \ldots, \widehat{\boldsymbol{w}}_n)$ and $\boldsymbol{\theta} = \left(\boldsymbol{H}^\top, \boldsymbol{\gamma}^\top\right)^\top$. Let $S = \mathrm{supp}(\boldsymbol{H})$, $S^C = [p+r] \backslash \boldsymbol{H}$. and let $\widehat{\boldsymbol{U}}_S$ denote the columns of $\widehat{\boldsymbol{U}}$ indexed by S. The factor-residualized active score matrix $\left(\boldsymbol{I}_n - P_{\widehat{\boldsymbol{F}}}\right)\widehat{\boldsymbol{U}}_S$ has full column rank with probability tending to one. Moreover, for

some constant $c_{\boldsymbol{U}} > 0$,

$$\lambda_{\min}\left[\frac{1}{n}\widehat{\boldsymbol{U}}_S^\top\left(\boldsymbol{I}_n - P_{\widehat{\boldsymbol{F}}}\right)\widehat{\boldsymbol{U}}_S\right] \geq c_{\boldsymbol{U}}$$

with probability tending to one, and

$$\left\|\frac{1}{n}\widehat{\boldsymbol{U}}_S^\top\left(\boldsymbol{I}_n - P_{\widehat{\boldsymbol{F}}}\right)\tilde{\boldsymbol{\varepsilon}}^*\right\|_2 = o_p(1).$$

In addition, for $\boldsymbol{W} = (\boldsymbol{w}_1, \ldots, \boldsymbol{w}_n)$ where $\boldsymbol{w}_i = \left(\boldsymbol{u}_i^\top, \boldsymbol{f}_i^\top\right)^\top$, there exist $\kappa_2 > \kappa_\infty > 0$ and $\tau \in (0, 0.5)$ such that

(1) $\left\|\left[\nabla_{SS}^2 L_n(\boldsymbol{Y}, \boldsymbol{W}\boldsymbol{\theta})\right]^{-1}\right\|_\ell \leq \frac{1}{4\kappa_\ell}$, for $\ell = 2$ and ∞,

(2) $\left\|\nabla_{S^C S}^2 L_n(\boldsymbol{Y}, \boldsymbol{W}\boldsymbol{\theta})\left[\nabla_{SS}^2 L_n(\boldsymbol{Y}, \boldsymbol{W}\boldsymbol{\theta})\right]^{-1}\right\|_\infty \leq 1 - 2\tau$,

where $\nabla_{S_1 S_2}^2 f(x) = \left(\nabla^2 f(x)\right)_{S_1 S_2}$.

Assumption 11. Denote $\boldsymbol{\gamma}^{(k)}(t) = \left(\gamma_1^{(k)}(t), \ldots, \gamma_{m_k}^{(k)}(t)\right)^\top$, $X_{f,i}^{(k)}(t) = \boldsymbol{\gamma}^{(k)}(t)^\top\boldsymbol{\Lambda}^{(k)}\boldsymbol{f}_i$ and $X_{u,i}^{(k)}(t) = \boldsymbol{\gamma}^{(k)}(t)^\top\boldsymbol{u}_i^{(k)}$. Assume that $\left\|\gamma_j^{(k)}(\cdot)\right\|_\infty \leq M_\gamma$ for $j = 1, \ldots, m_k, k = 1, \ldots, K$, and $\left\|X_{f,i}^{(k)}(\cdot)\right\|_\infty \leq \frac{1}{2}M_\gamma M_0\sqrt{\lambda_1^{(\boldsymbol{\Lambda})}}$ and $\left\|X_{u,i}^{(k)}(\cdot)\right\|_\infty \leq \frac{1}{2}M_\gamma M_0$ for some constant $M_0 > 2$, where $\lambda_1^{(\boldsymbol{\Lambda})}$ is the largest eigenvalue of the matrix $\boldsymbol{\Lambda}\boldsymbol{\Lambda}^\top$. In addition, there exists a $r \times r$ nonsingular matrix $\boldsymbol{V}_0$, and $\boldsymbol{V} = \begin{pmatrix} \boldsymbol{I}_p & \mathbf{0}_{p\times r} \\ \mathbf{0}_{r\times p} & \boldsymbol{V}_0 \end{pmatrix}$ such that for $\overline{\boldsymbol{W}} = \widehat{\boldsymbol{W}}\boldsymbol{V}$, we have $\|\overline{\boldsymbol{W}} - \boldsymbol{W}\|_{\max} \leq \frac{M_0}{2}$ and $\max_{j\in[p+r]}\left(\frac{1}{n}\sum_{i=1}^n |\bar{w}_{ij} - w_{ij}|^2\right)^{1/2} \leq \frac{2\kappa_\infty\tau}{3M_0M_2|S|}$, with w_{ij} and $\bar{w}_{ij}$ being the (i,j)-th element of $\boldsymbol{W}$ and $\overline{\boldsymbol{W}}$.

Proposition 2. (Identification after factor augmentation) Suppose that $\boldsymbol{H}$ is identified in the instrument-projected FPCA representation as stated in **Proposition 1** and that the post-factor relevance conditions in **Assumption 10** hold. Then the active coefficient vector $\boldsymbol{H}_S$ is uniquely identified asymptotically from

$$(\boldsymbol{I}_n - P_{\widehat{\boldsymbol{F}}})\boldsymbol{Y} = (\boldsymbol{I}_n - P_{\widehat{\boldsymbol{F}}})\widehat{\boldsymbol{U}}\boldsymbol{H} + (\boldsymbol{I}_n - P_{\widehat{\boldsymbol{F}}})\tilde{\boldsymbol{\varepsilon}}^*.$$

Therefore, factor residualization does not redefine the structural coefficient vector $\boldsymbol{H}$, provided that the same $\boldsymbol{H}$ governs both the shared-factor and idiosyncratic components in equation (5) of the main text. If the residual relevance condition fails in a coefficient direction, however, that direction of $\boldsymbol{H}$ cannot be separately identified from the residualized equation.

Proof. Restricting equation (6) of the main text to the active set S and premultiplying by

$n^{-1}\widehat{\boldsymbol{U}}_S^\top$ yields

$$\frac{1}{n}\widehat{\boldsymbol{U}}_S^\top\left(\boldsymbol{I}_n - P_{\widehat{\boldsymbol{F}}}\right)\boldsymbol{Y} = \frac{1}{n}\widehat{\boldsymbol{U}}_S^\top\left(\boldsymbol{I}_n - P_{\widehat{\boldsymbol{F}}}\right)\widehat{\boldsymbol{U}}_S\boldsymbol{H}_S + o_p(1),$$

where the final term follows from **Assumption 10**. The minimum-eigenvalue condition in **Assumption 10** implies that $\frac{1}{n}\widehat{\boldsymbol{U}}_S^\top\left(\boldsymbol{I}_n - P_{\widehat{\boldsymbol{F}}}\right)\widehat{\boldsymbol{U}}_S$ is nonsingular with probability tending to one. Hence $\boldsymbol{H}_S$ is uniquely identified asymptotically. Conversely, if a nonzero coefficient direction lies in the null space of $\left(\boldsymbol{I}_n - P_{\widehat{\boldsymbol{F}}}\right)\widehat{\boldsymbol{U}}_S$ coefficients along that direction cannot be distinguished from the residualized equation.

Remark 2. The shared-factor contribution $\widehat{\boldsymbol{F}}\widehat{\boldsymbol{\Lambda}}^\top\boldsymbol{H}$ remains part of the structural signal in equation (5); it is removed only from the estimating equation for $\boldsymbol{H}$. Thus, factor augmentation does not define $\boldsymbol{H}$ as an idiosyncratic-only effect. However, if a causal direction is supported only by the shared factor space and lacks sufficient residual variation, the current residualized estimator cannot separately recover that coordinate of $\boldsymbol{H}$. In that case, the shared-factor component may inform only lower-dimensional combinations such as $\widehat{\boldsymbol{\Lambda}}^\top\boldsymbol{H}$.

According to the results on the properties of the functional factor augmentation structure established by Ma et al. (Ma et al., 2025), we obtain the following result.

Theorem 1. Under **Assumptions 4-9**, using the Theorem 1 in Ma et al. (Ma et al., 2025), we have

$$\left\|\widehat{\boldsymbol{f}}_i^\top - \boldsymbol{f}_i^\top\right\|_2^2 = O_P\left(\left(\omega^2 + 1/n\right)\|\boldsymbol{a}_i\|_2^2 + \left\|\boldsymbol{u}_i^\top\boldsymbol{\Lambda}\boldsymbol{\Sigma}_{\boldsymbol{\Lambda}}^{-1}\right\|_2^2\right),$$

$$\left\|\widehat{\boldsymbol{u}}_i^\top - \boldsymbol{u}_i^\top\right\|_2^2 = O_P\left(\left(\omega^2 + 1/n\right)\|\boldsymbol{a}_i\|_2^2 + \left\|\boldsymbol{u}_i^\top\boldsymbol{\Lambda}\boldsymbol{\Sigma}_{\boldsymbol{\Lambda}}^{-1}\boldsymbol{\Lambda}^\top\right\|_2^2\right).$$

where $\boldsymbol{\Sigma}_{\boldsymbol{\Lambda}} = \operatorname{diag}\left\{\lambda_1^{(\boldsymbol{\Lambda})},\ldots,\lambda_r^{(\boldsymbol{\Lambda})}\right\}$, $\lambda_1^{(\boldsymbol{\Lambda})},\ldots,\lambda_r^{(\boldsymbol{\Lambda})}$ are the first r eigenvalues of $\boldsymbol{\Lambda}\boldsymbol{\Lambda}^\top$.

Remark 3. **Theorem 1** follows from the corresponding result stated in terms of $\widehat{\boldsymbol{a}}_i = P_{\boldsymbol{Z}}\boldsymbol{a}_i$. Note that $\|\widehat{\boldsymbol{a}}_i\|_2 = \|P_{\boldsymbol{Z}}\boldsymbol{a}_i\|_2 \leq \|\boldsymbol{a}_i\|_2$ since $P_{\boldsymbol{Z}}$ is an orthogonal projection matrix, the error bound remains valid, up to a larger constant when $\widehat{\boldsymbol{a}}_i$ is replaced by the FPCA scores without instrumental variable projection.

Furthermore, in line with the results of Ma et al. (Ma et al., 2025), the following conclusion holds for $\boldsymbol{H}$.

Theorem 2. Suppose **Assumptions 10-11** holds. Define $M = M_0^3 M_3 |S|^{3/2}$, and

$$\varepsilon^* = \max_{j\in[p+r]}\left|\frac{1}{n}\sum_{i=1}^{n}\bar{w}_{ij}\left[-Y_i + \widehat{\boldsymbol{a}}_i^\top\boldsymbol{H}\right]\right|.$$

If $\frac{7\varepsilon^*}{\tau} < \lambda < \frac{\kappa_2\kappa_\infty\tau}{12M\sqrt{|S|}}$, then $\text{supp}(\widehat{\boldsymbol{H}}) \subseteq \text{supp}(\boldsymbol{H})$ and

$$\left\|\widehat{\boldsymbol{H}} - \boldsymbol{H}\right\|_\infty \le \frac{6\lambda}{5\kappa_\infty}, \quad \left\|\widehat{\boldsymbol{H}} - \boldsymbol{H}\right\|_2 \le \frac{4\lambda\sqrt{|S|}}{\kappa_2}, \quad \left\|\widehat{\boldsymbol{H}} - \boldsymbol{H}\right\|_1 \le \frac{6\lambda|S|}{5\kappa_\infty}.$$

Remark 4. Relative to Ma et al. (Ma et al., 2025), using $\widehat{\boldsymbol{a}}_i$, which is obtained through instrumental variable projection of $\boldsymbol{a}_i$, in **Theorem 2** leads to slightly more restrictive lower bounds on the tuning parameter λ, and hence to larger constants in the corresponding error bounds for $\widehat{\boldsymbol{H}}$. Nevertheless, the admissible range of λ remains nonempty, and the convergence rates established in **Theorem 2** are unchanged.

Based on the error bound established in **Theorem 2**, we can directly obtain an error bound for $\beta^{(k)}(t), k = 1, \dots, K$.

Corollary 1. Under the assumptions in **Theorem 2**, it holds that

$$\left\|\widehat{\beta}^{(k)}(t) - \beta^{(k)}(t)\right\|_\infty \le \frac{6M_\gamma\lambda|S|}{5\kappa_\infty},$$

$$\left\|\widehat{\beta}^{(k)}(t) - \beta^{(k)}(t)\right\|_2 \le \frac{4M_\gamma\sqrt{Ts_n}\lambda\sqrt{|S|}}{\kappa_2},$$

$$\left\|\widehat{\beta}^{(k)}(t) - \beta^{(k)}(t)\right\|_1 \le \frac{6M_\gamma T\lambda|S|}{5\kappa_\infty}.$$

Proof. Recall that

$$\beta^{(k)}(t) = \sum_{j=1}^{m_k} \eta_j^{(k)}\gamma_j^{(k)}(t), \quad \widehat{\beta}^{(k)}(t) = \sum_{j=1}^{m_k} \hat{\eta}_j^{(k)}\gamma_j^{(k)}(t).$$

Hence

$$\widehat{\beta}^{(k)}(t) - \beta^{(k)}(t) = \sum_{j=1}^{m_k}\left(\widehat{\eta}_j^{(k)} - \eta_j^{(k)}\right)\gamma_j^{(k)}(t).$$

(1) **Sup-norm bound**

By the triangle inequality and the uniform boundedness of basis functions,

$$\left|\widehat{\beta}^{(k)}(t) - \beta^{(k)}(t)\right| \le M_\gamma \sum_{j=1}^{m_k}\left|\widehat{\eta}_j^{(k)} - \eta_j^{(k)}\right|.$$

Taking supremum over t,

$$\left\|\widehat{\beta}^{(k)}(t) - \beta^{(k)}(t)\right\|_\infty \le M_\gamma\left\|\widehat{\boldsymbol{\eta}}^{(k)} - \boldsymbol{\eta}^{(k)}\right\|_1 \le M_\gamma\|\widehat{\boldsymbol{H}} - \boldsymbol{H}\|_1.$$

Applying **Theorem 2** yields

$$\left\|\widehat{\beta}^{(k)}(t) - \beta^{(k)}(t)\right\|_\infty \leq \frac{6M_\gamma \lambda |S|}{5\kappa_\infty}.$$

(2) L_2**-norm bound**

By definition,

$$\left\|\widehat{\beta}^{(k)}(t) - \beta^{(k)}(t)\right\|_2^2 = \int_0^T \left(\sum_{j=1}^{m_k} \left(\widehat{\eta}_j^{(k)} - \eta_j^{(k)}\right) \gamma_j^{(k)}(t) \right)^2 dt$$

For each fixed t, applying the Cauchy–Schwarz inequality yields

$$\int_0^T \left(\sum_{j=1}^{m_k} \left(\widehat{\eta}_j^{(k)} - \eta_j^{(k)}\right) \gamma_j^{(k)}(t) \right)^2 dt \leq \left\|\widehat{\boldsymbol{\eta}}^{(k)} - \boldsymbol{\eta}^{(k)}\right\|_2^2 \int_0^T \sum_{j=1}^{m_k} \gamma_j^{(k)}(t)^2 dt.$$

Substituting this bound into the integral gives

$$\left\|\widehat{\beta}^{(k)}(t) - \beta^{(k)}(t)\right\|_2^2 \leq \left\|\widehat{\boldsymbol{\eta}}^{(k)} - \boldsymbol{\eta}^{(k)}\right\|_2^2 \int_0^T \sum_{j=1}^{m_k} \gamma_j^{(k)}(t)^2 dt.$$

By apply $\left\|\gamma_j^{(k)}(\cdot)\right\|_\infty \leq M_\gamma$, we have

$$\gamma_j^{(k)}(t)^2 \leq M_\gamma^2 \quad \text{for all } t \in [0, T],$$

and hence

$$\int_0^T \sum_{j=1}^{m_k} \gamma_j^{(k)}(t)^2 dt \leq T m_k M_\gamma^2.$$

Combining the above inequalities yields

$$\left\|\widehat{\beta}^{(k)}(t) - \beta^{(k)}(t)\right\|_2 \leq \sqrt{T m_k} M_\gamma \left\|\widehat{\boldsymbol{\eta}}^{(k)} - \boldsymbol{\eta}^{(k)}\right\|_2 \leq .$$

Finally, since $m_k \leq s_n$ by **Assumption 5**,

$$\left\|\widehat{\beta}^{(k)}(t) - \beta^{(k)}(t)\right\|_2 \leq \frac{4M_\gamma \sqrt{T s_n} \lambda \sqrt{|S|}}{\kappa_2}.$$

(3) L_1**-norm bound**

By Fubini's theorem and triangle inequality,

$$\left\|\widehat{\beta}^{(k)}(t)-\beta^{(k)}(t)\right\|_1 \leq \sum_{j=1}^{m_k}\left|\widehat{\eta}_j^{(k)}-\eta_j^{(k)}\right|\left\|\gamma_j^{(k)}(t)\right\|_1.$$

Note that

$$\int_0^T\left|\gamma_j^{(k)}(t)\right|dt \leq T\left\|\gamma_j^{(k)}(t)\right\|_\infty \leq TM_\gamma,$$

we have

$$\left\|\widehat{\beta}^{(k)}(t)-\beta^{(k)}(t)\right\|_1 \leq TM_\gamma\left\|\widehat{\boldsymbol{\eta}}^{(k)}-\boldsymbol{\eta}^{(k)}\right\|_1 \leq TM_\gamma\|\widehat{\boldsymbol{H}}-\boldsymbol{H}\|_1.$$

Applying **Theorem 2** yields

$$\left\|\widehat{\beta}^{(k)}(t)-\beta^{(k)}(t)\right\|_1 \leq \frac{6M_\gamma T\lambda|S|}{5\kappa_\infty}.$$

S2: Additional Results

S2.1: Exposure-specific estimation errors under varying sample sizes

This section provides detailed estimation error results for each exposure-specific time-varying causal effect function under varying sample sizes, complementing the aggregate results reported in Figure 2 of the main text.

Figures S1–S4 present the total error distributions for the first four nonzero effect functions. Figure S5 summarizes the aggregated estimation error for the remaining zero effect functions.

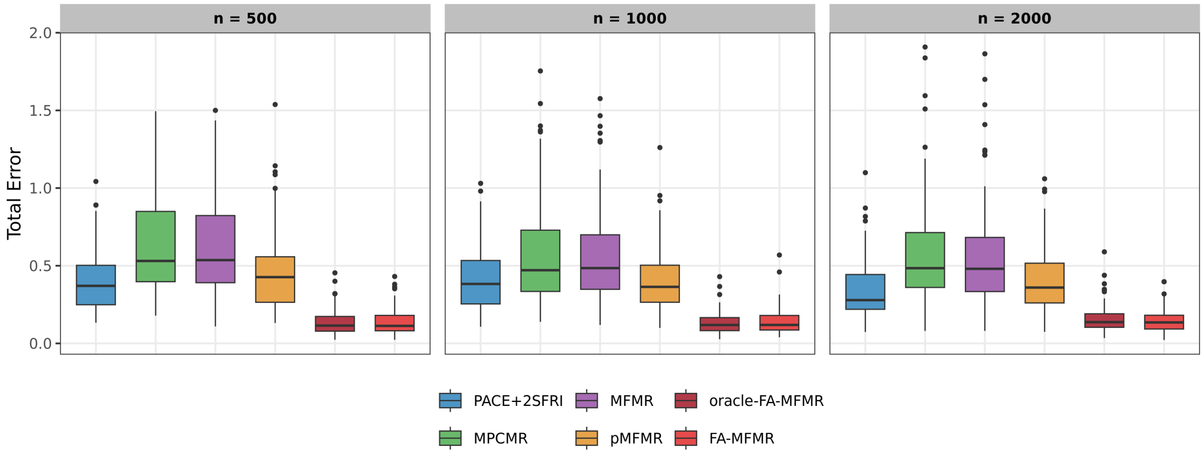

Figure S1: Total error of $\widehat{\beta}_1(t)$ under the settings $K = 10$ exposures and $r = 3$ latent factors. Each panel corresponds to a different sample size $n \in \{500, 1000, 2000\}$, and each box summarizes the distribution across 100 simulation replicates.

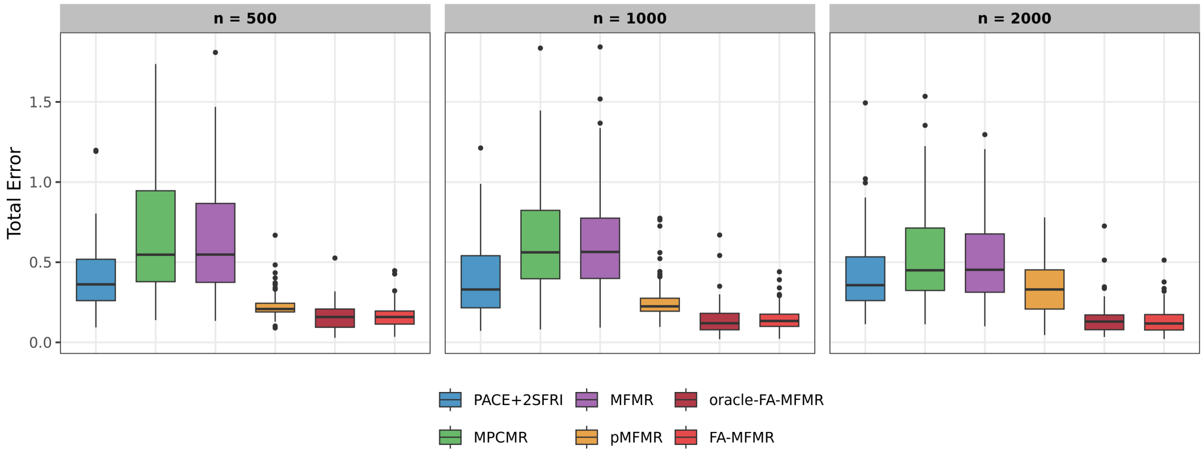

Figure S2: Total error of $\widehat{\beta}_2(t)$ under the settings $K = 10$ exposures and $r = 3$ latent factors. Each panel corresponds to a different sample size $n \in \{500, 1000, 2000\}$, and each box summarizes the distribution across 100 simulation replicates.

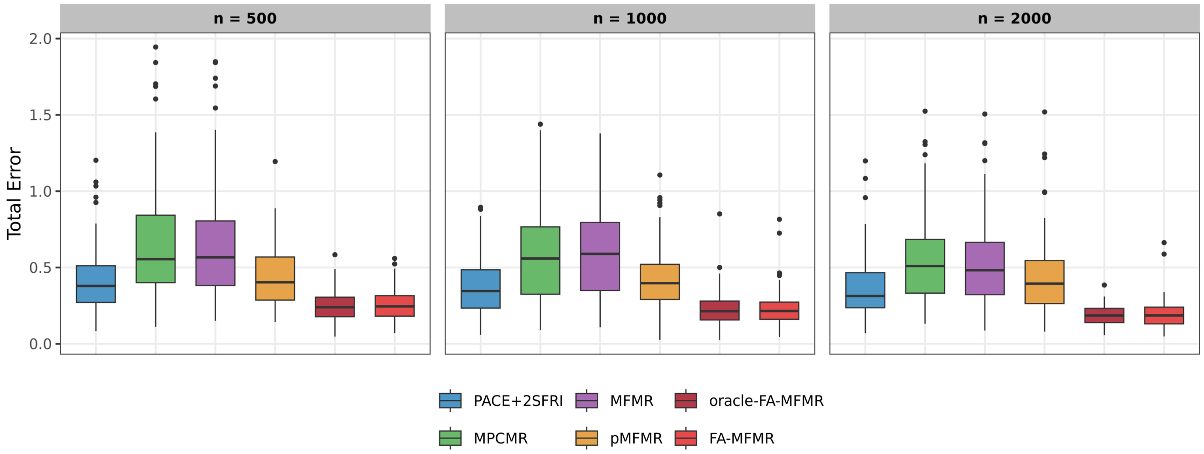

Figure S3: Total error of $\widehat{\beta}_3(t)$ under the settings $K = 10$ exposures and $r = 3$ latent factors. Each panel corresponds to a different sample size $n \in \{500, 1000, 2000\}$, and each box summarizes the distribution across 100 simulation replicates.

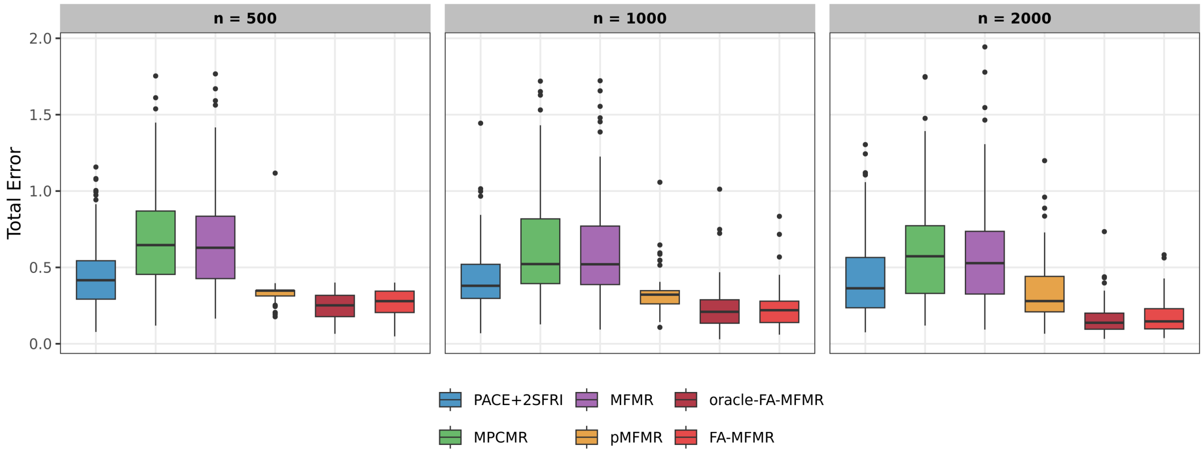

Figure S4: Total error of $\widehat{\beta}_4(t)$ under the settings $K = 10$ exposures and $r = 3$ latent factors. Each panel corresponds to a different sample size $n \in \{500, 1000, 2000\}$, and each box summarizes the distribution across 100 simulation replicates.

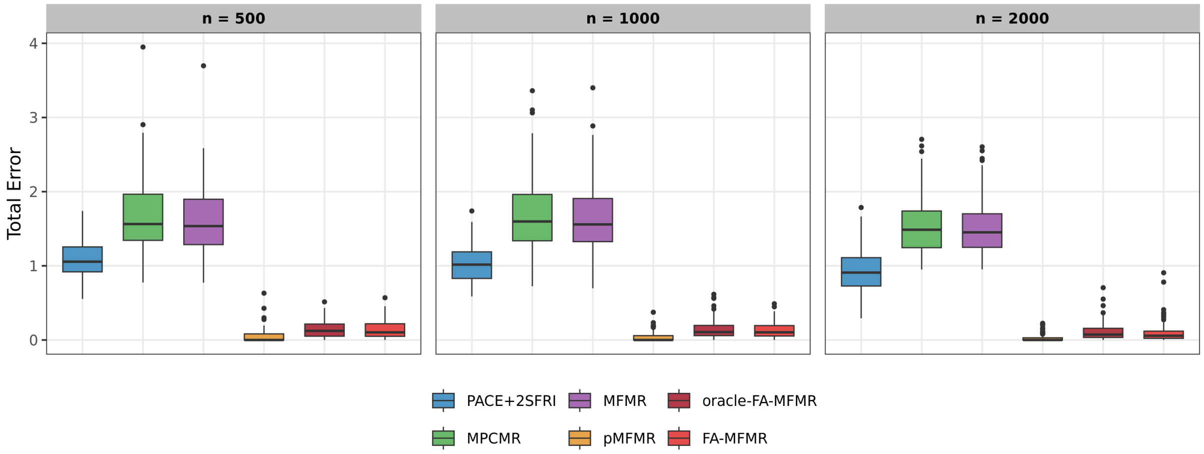

Figure S5: Total error of $\widehat{\beta}_5(t), \ldots, \widehat{\beta}_{10}(t)$ under the settings $K = 10$ exposures and $r = 3$ latent factors. Each panel corresponds to a different sample size $n \in \{500, 1000, 2000\}$, and each box summarizes the distribution across 100 simulation replicates.

S2.2: Exposure-specific estimation errors under different latent-factor complexities

This section provides detailed estimation error results for each exposure-specific time-varying causal effect function under different latent-factor complexities, complementing the aggregate results reported in Figure 3 of the main text.

Figures S6–S9 present the total error distributions for the first four nonzero effect functions. Figure S10 summarizes the aggregated estimation error for the remaining zero effect functions.

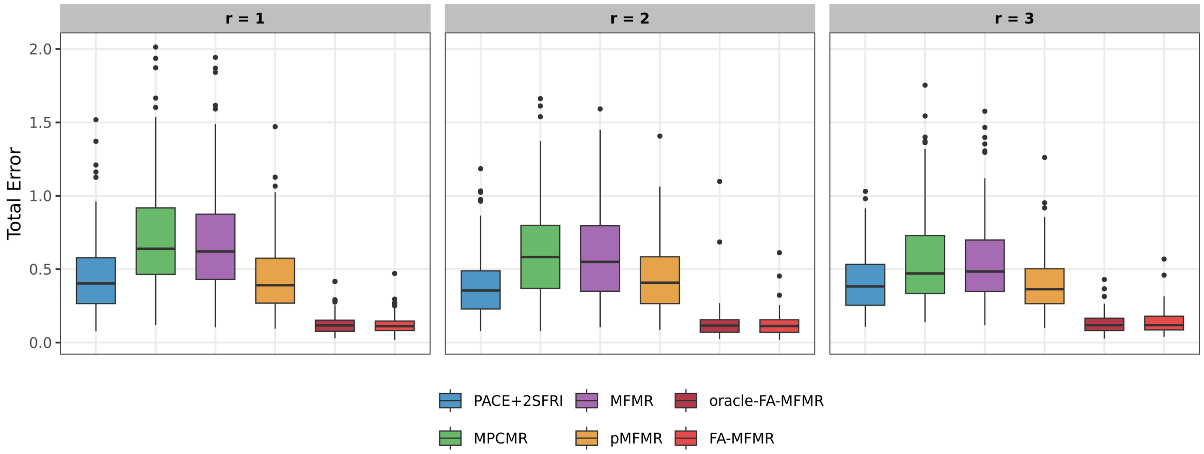

Figure S6: Total error of $\widehat{\beta}_1(t)$ under the settings $n = 1000$ and $K = 10$ exposures. Each panel corresponds to a different latent-factor complexity $r \in \{1, 2, 3\}$, and each box summarizes the distribution across 100 simulation replicates.

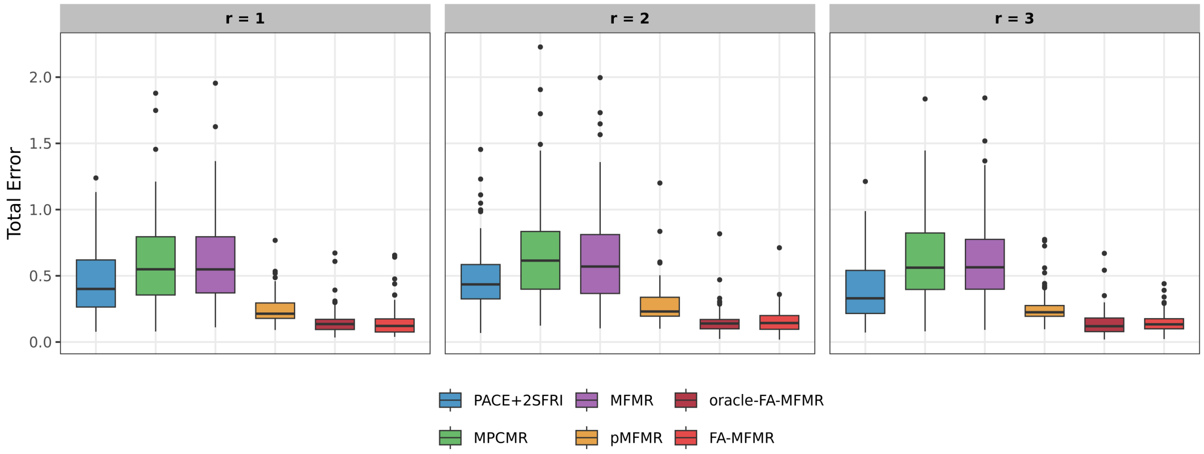

Figure S7: Total error of $\widehat{\beta}_2(t)$ under the settings $n = 1000$ and $K = 10$ exposures. Each panel corresponds to a different latent-factor complexity $r \in \{1, 2, 3\}$, and each box summarizes the distribution across 100 simulation replicates.

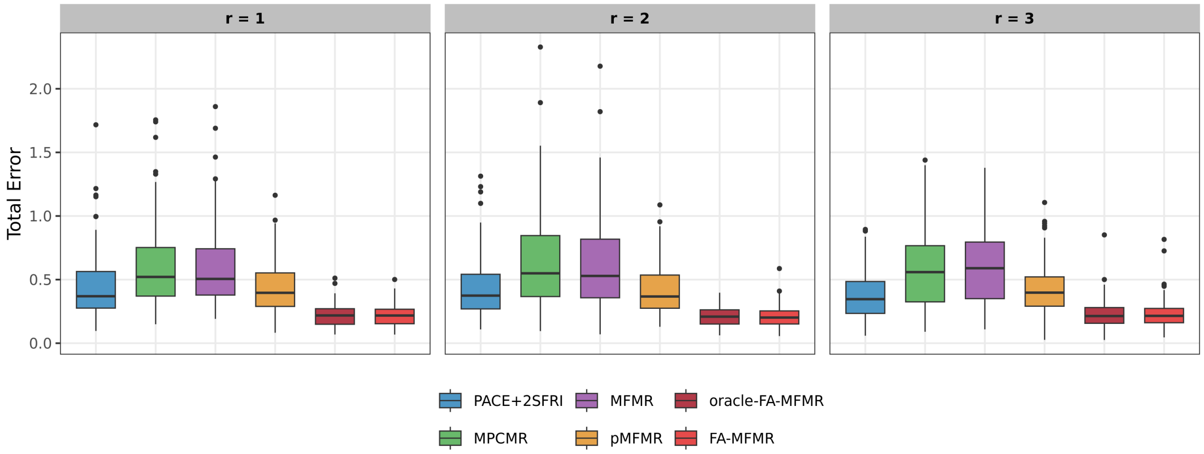

Figure S8: Total error of $\widehat{\beta}_3(t)$ under the settings $n = 1000$ and $K = 10$ exposures. Each panel corresponds to a different latent-factor complexity $r \in \{1, 2, 3\}$, and each box summarizes the distribution across 100 simulation replicates.

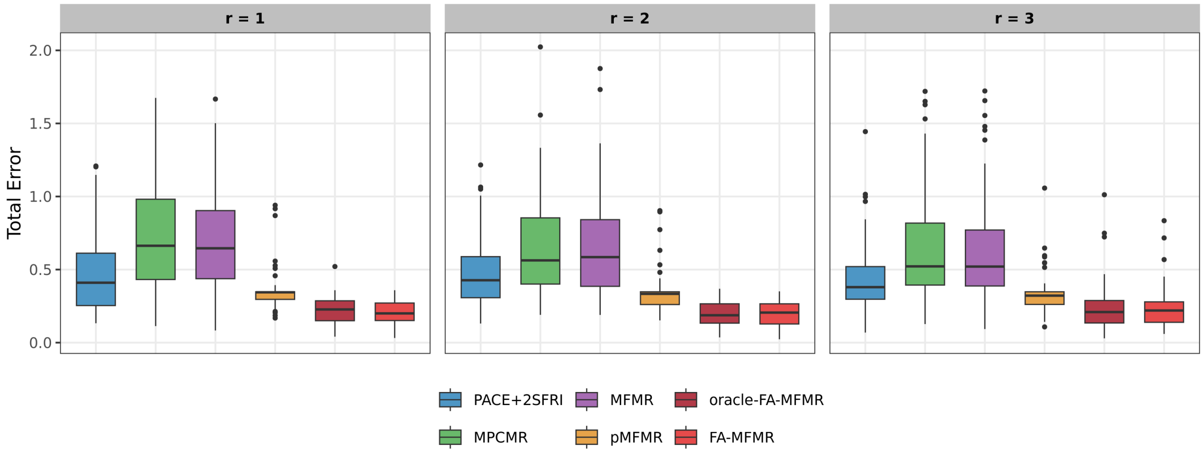

Figure S9: Total error of $\widehat{\beta}_4(t)$ under the settings $n = 1000$ and $K = 10$ exposures. Each panel corresponds to a different latent-factor complexity $r \in \{1, 2, 3\}$, and each box summarizes the distribution across 100 simulation replicates.

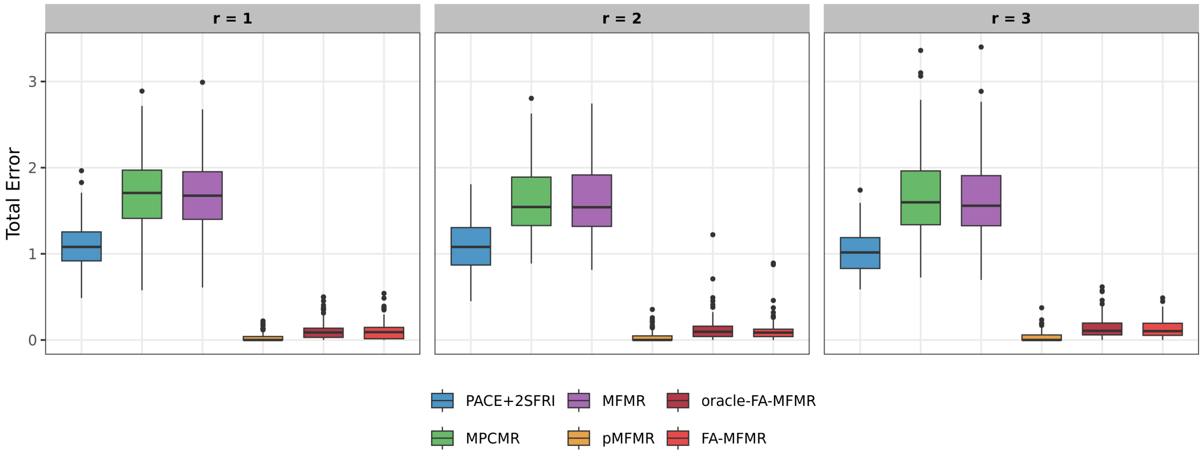

Figure S10: Total error of $\widehat{\beta}_5(t), \ldots, \widehat{\beta}_{10}(t)$ under the settings $n = 1000$ and $K = 10$ exposures. Each panel corresponds to a different latent-factor complexity $r \in \{1, 2, 3\}$, and each box summarizes the distribution across 100 simulation replicates.

S2.3: Exposure-specific estimation errors under varying exposure dimensions

This section provides detailed estimation error results for each exposure-specific time-varying causal effect function different under varying exposure dimensions, complementing the aggregate results reported in Figure 4 of the main text.

Figures S11–S14 present the total error distributions for the first four nonzero effect functions. Figure S15 summarizes the estimation error for $\widehat{\beta}_5(t)$.

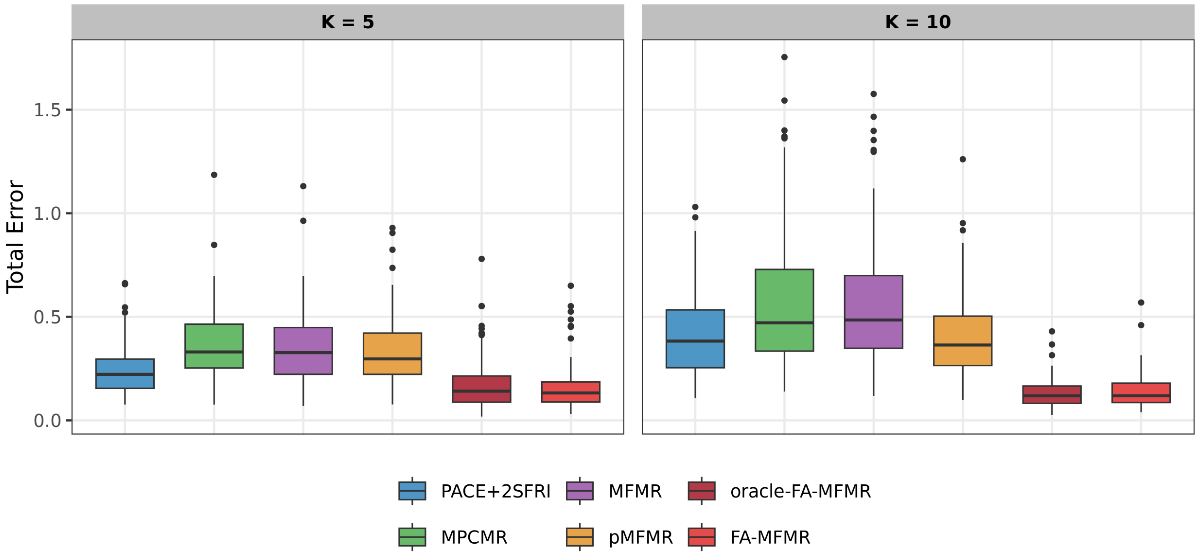

Figure S11: Total error of $\widehat{\beta}_1(t)$ under the settings $n = 1000$ and $r = 3$ latent factors. Each panel corresponds to a different number of exposures $K \in \{5, 10\}$, and each box summarizes the distribution across 100 simulation replicates.

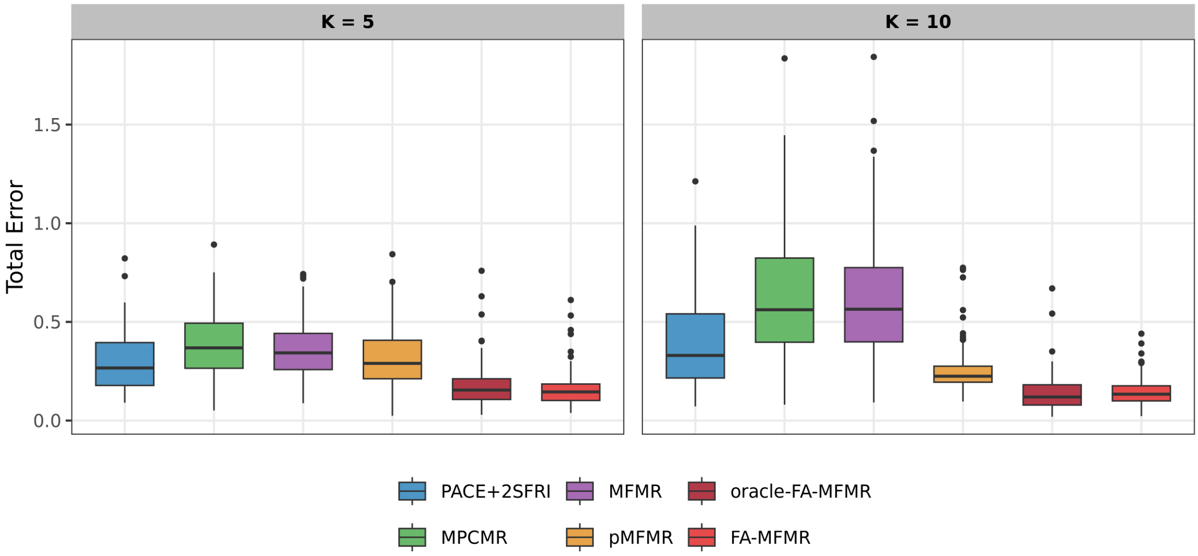

Figure S12: Total error of $\widehat{\beta}_2(t)$ under the settings $n = 1000$ and $r = 3$ latent factors. Each panel corresponds to a different number of exposures $K \in \{5, 10\}$, and each box summarizes the distribution across 100 simulation replicates.

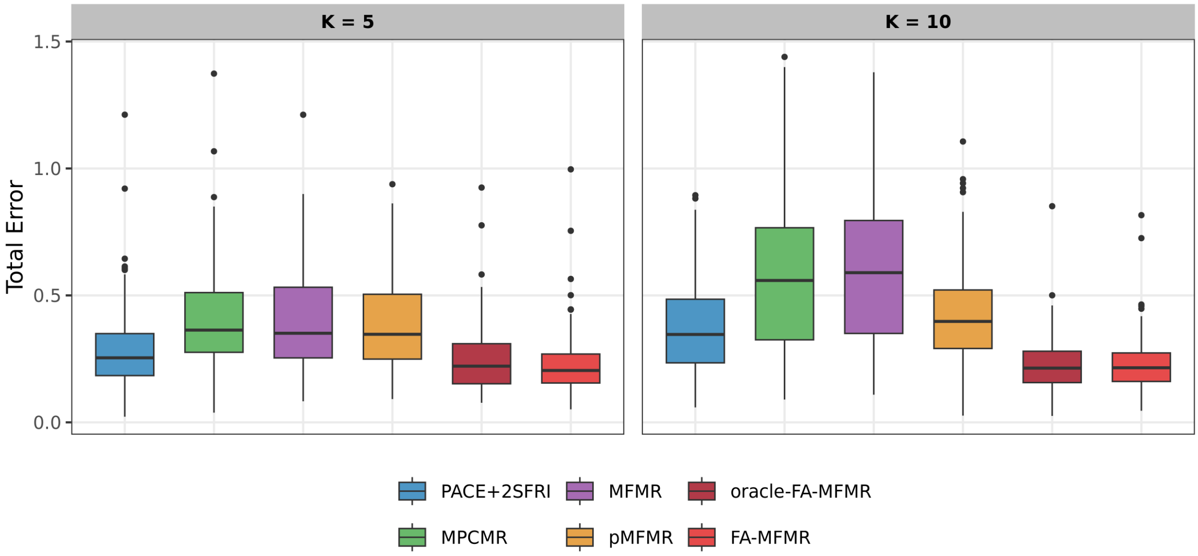

Figure S13: Total error of $\widehat{\beta}_3(t)$ under the settings $n = 1000$ and $r = 3$ latent factors. Each panel corresponds to a different number of exposures $K \in \{5, 10\}$, and each box summarizes the distribution across 100 simulation replicates.

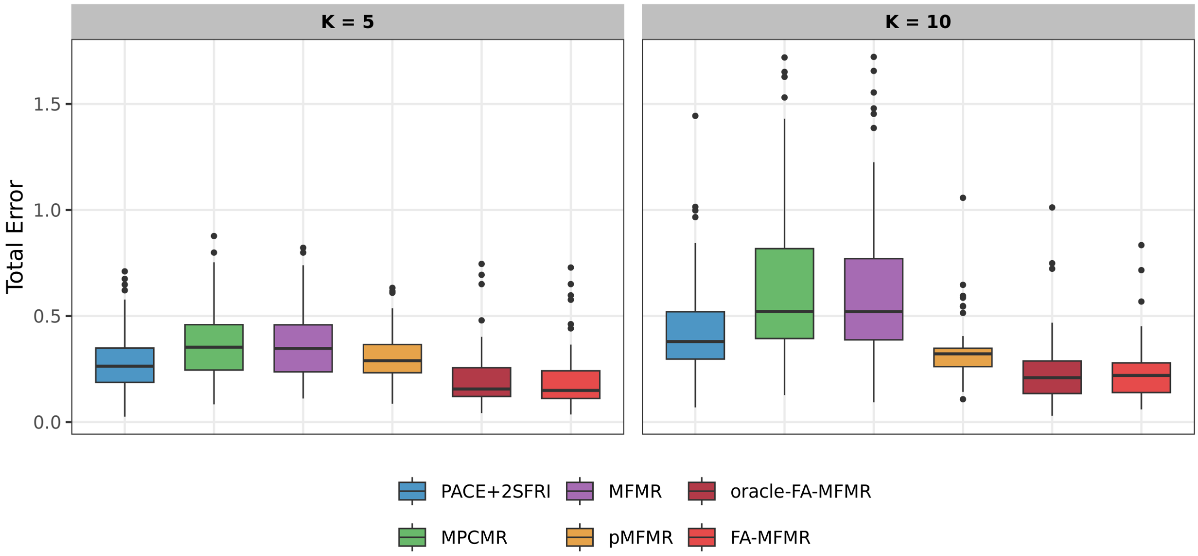

Figure S14: Total error of $\widehat{\beta}_4(t)$ under the settings $n = 1000$ and $r = 3$ latent factors. Each panel corresponds to a different number of exposures $K \in \{5, 10\}$, and each box summarizes the distribution across 100 simulation replicates.

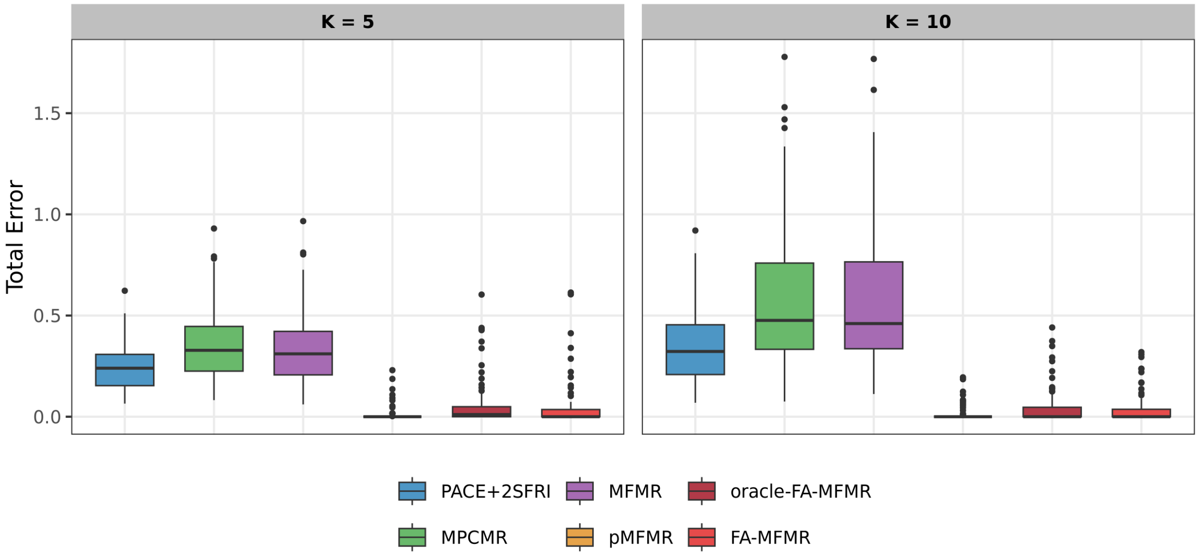

Figure S15: Total error of $\widehat{\beta}_5(t)$ under the settings $n = 1000$ and $r = 3$ latent factors. Each panel corresponds to a different number of exposures $K \in \{5, 10\}$, and each box summarizes the distribution across 100 simulation replicates.

S2.4: Sensitivity to the ordering of time-varying effect functions under varying exposure dimensions

Under varying exposure dimensions, this section compares the total estimation errors of different methods when the ordering of the time-varying effect functions is altered. As in the main analysis, we consider both $K = 5$ and $K = 10$. However, unlike the previous setting in which the additional exposures introduced at $K = 10$ are specified to have null time-varying effects, here we allow both early- and late-indexed coefficient functions to be either null or non-null. To reflect this structure, we modify the configuration of the ten time-varying effect functions such that $\beta^{(1)}(t) = 2(t - 0.5)$, $\beta^{(2)}(t) = 4(t - 0.5)^2$, $\beta^{(6)}(t) = \sin(2\pi t)$, $\beta^{(7)}(t) = \exp(-\frac{(t-0.5)^2}{0.2^2})$, while the remaining effect functions are set to zero. Figure S16 presents the corresponding results.

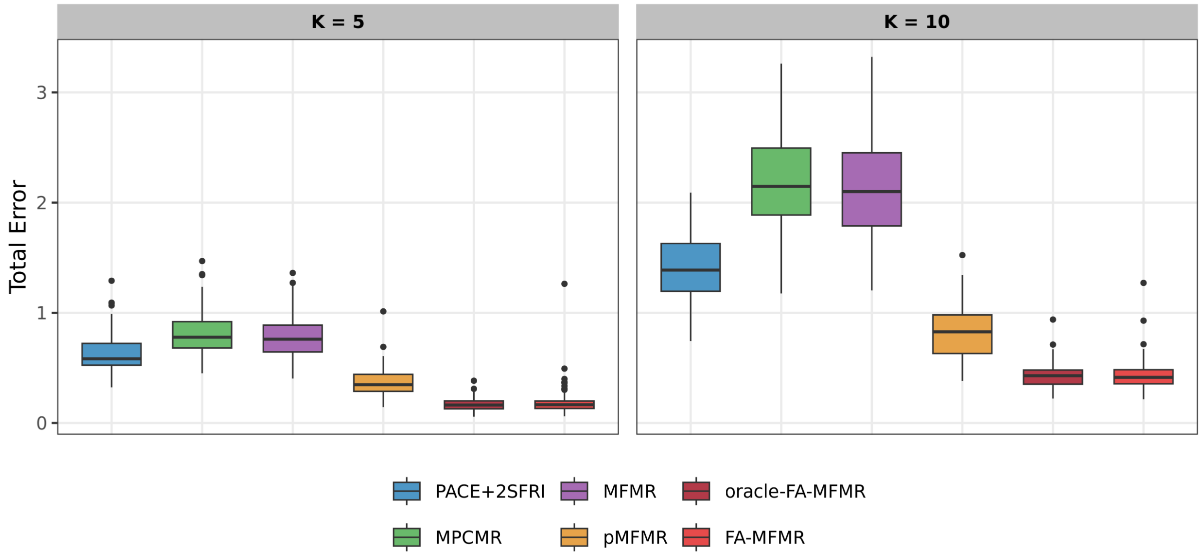

Figure S16: Total Error of the estimated time-varying causal effect functions under the alternative effect ordering, with $n = 1000$ and $r = 3$ latent factors. Each panel corresponds to a different number of exposures $K \in \{5, 10\}$, and each box summarizes the distribution across 100 simulation replicates.

S2.5: Sensitivity to causal-signal alignment with the common-factor space

To further examine identification after factor augmentation, we conduct a targeted sensitivity analysis in which the causal coefficient direction is progressively aligned with the low-dimensional factor structure shared across the longitudinal exposures. This experiment is motivated by the fact that factor residualization changes the variation used to distinguish the exposure-specific coefficients, while it does not redefine the structural coefficient vector itself.

We consider one representative setting of the simulation design with $n = 1000$ subjects,

$K = 10$ longitudinal exposures, and $r = 3$ latent factors. The first four exposure coefficient functions are nonzero, whereas the remaining six are null. The sparse observation mechanism, measurement error, Brownian-motion confounding, instrument generation, idiosyncratic score component, and outcome-generating mechanism are otherwise kept the same as in the main simulation study.

The true coefficient functions $\beta^{(k)}(t)$ are kept fixed across all alignment settings. Because the longitudinal trajectories are generated using B-spline basis functions $\gamma_j(t), j = 1, \ldots, 6$, we first obtain the score-level coefficient vector implied by these same coefficient functions. For exposure k, define

$$\eta_{0j}^{(k)} = \int_0^T \gamma_j(t)\beta^{(k)}(t)dt, \quad j = 1, \ldots, 6$$

and let $\boldsymbol{H}_0 = \left(\boldsymbol{\eta}_0^{(1)\top}, \ldots, \boldsymbol{\eta}_0^{(K)\top}\right)^\top$, where $\boldsymbol{\eta}_0^{(k)} = \left(\eta_{01}^{(k)}, \ldots, \eta_{0m}^{(k)}\right)^\top$.

We then vary only the orientation of the generating common-factor loading space relative to this fixed causal coefficient direction. Let $\boldsymbol{\Lambda}_\rho$ denote the generating loading matrix and define $P_{\boldsymbol{\Lambda}_\rho} = \boldsymbol{\Lambda}_\rho \left(\boldsymbol{\Lambda}_\rho^\top \boldsymbol{\Lambda}_\rho\right)^{-1} \boldsymbol{\Lambda}_\rho^\top$. The degree of alignment is quantified by

$$\rho_{\boldsymbol{F}} = \frac{\|P_{\boldsymbol{\Lambda}_\rho}\boldsymbol{H}_0\|_2^2}{\|\boldsymbol{H}_0\|_2^2}.$$

Thus, $\rho_{\boldsymbol{F}}$ measures the degree to which the fixed causal coefficient direction overlaps with the shared factor-loading space.

For each replicate, a loading matrix is first generated according to the original simulation mechanism. Its loading-space orientation is then adjusted toward the fixed direction $\boldsymbol{H}_0$ to attain the desired value of $\rho_{\boldsymbol{F}}$, while preserving the original loading magnitude and cross-factor Gram structure. The resulting score matrix continues to follow the same factor model,

$$\boldsymbol{A} = \boldsymbol{F}\boldsymbol{\Lambda}_\rho^\top + \boldsymbol{U},$$

with the generation of $\boldsymbol{F}$ and $\boldsymbol{U}$ otherwise unchanged. Therefore, this sensitivity experiment changes the geometric relationship between the fixed causal coefficient direction and the shared dependence structure, rather than changing the underlying causal coefficient functions or the remaining data-generating mechanism.

We consider three alignment settings, $\rho_{\boldsymbol{F}} = 0, 0.50, 0.75$, representing low, moderate, and high alignment, respectively. Each setting is repeated 100 times.

Because FA-MFMR is ultimately fitted using the FPCA representation recovered from the sparsely observed trajectories, the same true coefficient functions are additionally represented in the retained FPCA basis within each simulation replicate. Let $\widehat{\gamma}_j^{(k)}(t)$ denote the retained FPCA eigenfunctions. We calculate

$$\eta_{0j,\mathrm{FPCA}}^{(k)} = \int_0^T \beta^{(k)}(t)\widehat{\gamma}_j^{(k)}(t)dt$$

and stack these coefficients to form $\boldsymbol{H}_0^{\mathrm{FPCA}}$. This vector is used only evaluating the following diagnostic quantities. First, we measure the realized alignment between the true causal coefficient direction and the estimate factor-loading space as

$$\widehat{\rho}_{\boldsymbol{F}} = \frac{\|P_{\widehat{\boldsymbol{\Lambda}}}\boldsymbol{H}_0^{\mathrm{FPCA}}\|_2^2}{\|\boldsymbol{H}_0^{\mathrm{FPCA}}\|_2^2},$$

where $P_{\widehat{\boldsymbol{\Lambda}}} = \widehat{\boldsymbol{\Lambda}}\left(\widehat{\boldsymbol{\Lambda}}^\top\widehat{\boldsymbol{\Lambda}}\right)^{-1}\widehat{\boldsymbol{\Lambda}}^\top$. Second, to quantify the amount of residual variation remaining specifically along the true causal direction after factor adjustment, we define

$$R_{\boldsymbol{U}} = \frac{1}{n}\left\|\widetilde{\boldsymbol{U}}\boldsymbol{H}_0^{\mathrm{FPCA}}\right\|_2^2,$$

where $\widetilde{\boldsymbol{U}} = \left(I_n - P_{\widehat{\boldsymbol{F}}}\right)\widehat{\boldsymbol{U}}$. A smaller $R_{\boldsymbol{U}}$ therefore indicates that less of the residual score variation lies along the true causal coefficient direction.

Table S1 summarizes the results over 100 simulation replicates.

Table S1: Sensitivity of FA-MFMR to causal-coefficient alignment with the common-factor loading space. Values are reported as mean (SD) over 100 simulation replicates. $\rho_{\boldsymbol{F}}$ denotes the target generating alignment; $\hat{\rho}_{\boldsymbol{F}}$ denotes the realized alignment with the estimated factor-loading space after FPCA and IV projection; $R_{\boldsymbol{U}}$ denotes the residual directional signal after factor adjustment. The nonzero-effect MSE is the sum of the MSEs for the four nonzero coefficient functions evaluated at $t = 10, 20, 30$, and 40.

Alignment setting	Target $\rho_{\boldsymbol{F}}$	$\widehat{\rho}_{\boldsymbol{F}}$	$R_{\boldsymbol{U}}$	Nonzero-effect MSE
Low	0.00	0.011 (0.014)	73.13	0.174 (0.123)
Moderate	0.50	0.525 (0.162)	61.00	0.513 (0.407)
High	0.75	0.695 (0.137)	44.39	0.725 (0.329)

Overall, stronger alignment between the causal coefficient direction and the common-factor space is associated with less informative post-factor variation and reduced finite-sample precision for the exposure-specific coefficient estimates. This pattern is consistent with the post-factor identification argument in Section S1: factor residualization does not redefine the structural co-

efficient vector $\boldsymbol{H}$, but accurate recovery of its individual coordinates requires sufficient residual variation after adjustment for the shared factor structure.

S2.6: Pleiotropy and influence sensitivity analyses in the PPMI cohort

To assess the robustness of the PPMI results to potentially invalid or influential instruments, we conduct a series of complementary sensitivity analyses. These analyses include covariate-adjusted SNP–outcome association screening, instrument-level heterogeneity diagnostics, leave-one-SNP and leave-one-biomarker analyses, and an exploratory scalar multivariable MR sensitivity analysis. Because factor augmentation addresses dependence among the longitudinal exposure trajectories rather than violations of the exclusion restriction, these analyses separately assess the potential influence of horizontal pleiotropy and individual instruments on the PPMI findings.

1. Direct SNP–outcome associations and instrument-level heterogeneity

We first examine marginal associations between each of the 231 retained SNPs and NP3TOT in the final analytic cohort. For SNP j, we fit

$$Y_i = \alpha_{0j} + \Gamma_j Z_{ij} + \mathbf{C}_i^\top \boldsymbol{\delta}_j + \varepsilon_{ij},$$

where $\mathbf{C}_i$ contains sex, chronological age at the selected NP3TOT assessment, and the first 10 genetic ancestry principal components. We obtain the estimated SNP-outcome association $\widehat{\Gamma}_j$, its standard error, and the corresponding P-value. We apply the Benjamini-Hochberg procedure to account for multiple testing. Because a marginal SNP-outcome association can arise through the modeled biomarker pathways, these associations serve as instrument-level diagnostics rather than direct evidence of horizontal pleiotropy.

We additionally evaluate multivariable instrument heterogeneity using the biomarker measurements at visit V08. For instrument-level screening, we calculate the first-order residual heterogeneity score

$$q_j = w_j \left(\widehat{\Gamma}_j - \widehat{\gamma}_j^\top \widehat{\boldsymbol{\theta}}\right)^2,$$

where $\widehat{\gamma}_j$ denotes the vector of SNP-biomarker associations and w_j is the inverse variance of the SNP-outcome association. Larger q_j values identify instruments contributing more strongly to residual heterogeneity. We use the covariance-adjusted modified Cochran's Q statistic as the

global multivariable heterogeneity diagnostic.

Among the 231 retained instruments, 10 SNPs show nominal covariate-adjusted associations with NP3TOT at $P < 0.05$, whereas none remains significant after Benjamini-Hochberg correction (Figure S17A). The instrument-level residual heterogeneity scores identify several SNPs with relatively larger contributions, but the global heterogeneity statistic is $Q = 163.71, P = 0.997$, providing no evidence of excess multivariable instrument heterogeneity in this diagnostic analysis (Figure S17B). These results do not establish the absence of horizontal pleiotropy and are interpreted only as instrument-level diagnostics.

2. Leave-one-SNP analysis

We next assess whether the estimated coefficient functions are disproportionately driven by individual genetic instruments. Each of the 231 SNPs is removed in turn, and the instrument dependent FA-MFMR estimation steps are repeated using the remaining instruments. For exposure k, we compare the resulting coefficient function $\widehat{\beta}^{(k)}_{(-j)}(t)$ with the full-data estimate $\widehat{\beta}^{(k)}(t)$. We summarize the collection of leave-one-SNP estimates by the pointwise range

$$\left[\min_j \widehat{\beta}^{(k)}_{(-j)}(t), \max_j \widehat{\beta}^{(k)}_{(-j)}(t)\right],$$

which represents an influence envelope rather than a confidence interval.

The principal functional patterns for albumin, basophil count, neutrophil count, and white blood cell count generally remain recognizable across the leave-one-SNP analyses (Figure S18). Albumin and basophil count show relatively limited variation under individual SNP removal, whereas neutrophil count and, in particular, white blood cell count show greater sensitivity to the most influential instruments. Nevertheless, the analyses do not indicate that the principal PPMI findings are universally driven by a single retained SNP.

3. Leave-one-biomarker analysis

We further evaluate sensitivity to the composition of the multivariable exposure set. Each biomarker is omitted in turn together with the SNPs assigned to that biomarker, and the complete FA-MFMR model is refitted using the remaining 12 biomarkers while retaining the same analytic cohort. For every retained biomarker g, we quantify the difference between the reduced-

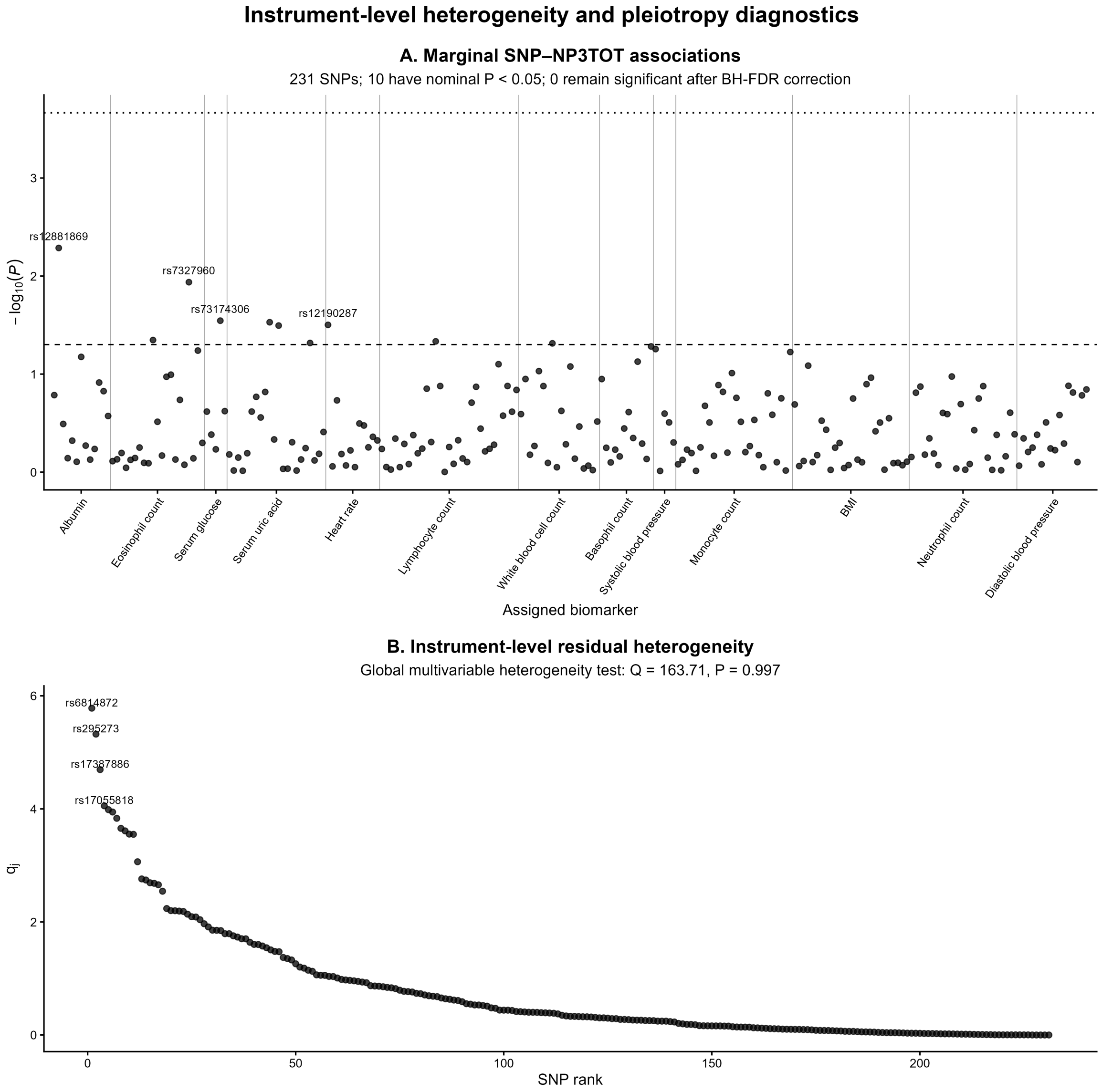

Figure S17: **Direct SNP–outcome associations and instrument-level heterogeneity diagnostics in the PPMI analysis. (A)** Covariate-adjusted associations between the 231 retained SNPs and NP3TOT. SNPs are grouped according to their assigned biomarkers. The dashed horizontal line denotes nominal $P = 0.05$, and the dotted line denotes the Bonferroni threshold $P = 0.05/231$. **(B)** SNP-specific first-order residual heterogeneity scores q_j, ranked from largest to smallest. The covariance-adjusted global multivariable heterogeneity diagnostic gives $Q = 163.71$ and $P = 0.997$.

model and full-model coefficient functions using

$$\text{RMSE}_{-k,g} = \left[\frac{1}{|\mathcal{T}|} \sum_{t \in \mathcal{T}} \left\{ \widehat{\beta}^{(g)}_{(-k)}(t) - \widehat{\beta}^{(g)}(t) \right\}^2 \right]^{1/2}.$$

The leave-one-biomarker analysis shows that sensitivity is concentrated in specific biomarker combinations rather than being uniform across the multivariable model (Figure S19). Omission of neutrophil count produces the most pronounced changes across several retained coefficient

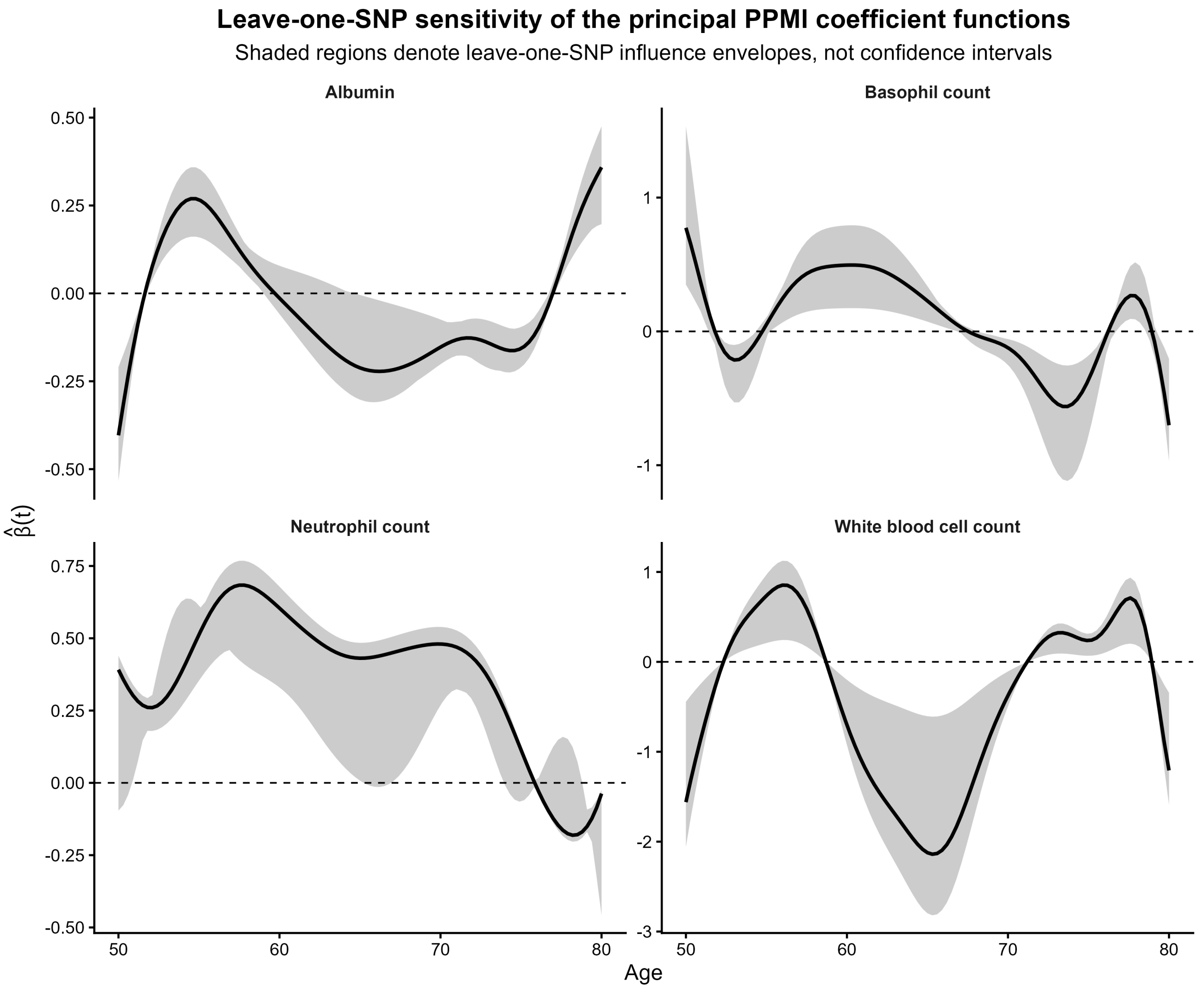

Figure S18:
textbfLeave-one-SNP sensitivity of the principal PPMI coefficient functions. The solid curves denote the coefficient functions estimated from the complete instrument set for albumin, basophil count, neutrophil count, and white blood cell count. The shaded regions represent the pointwise ranges of the coefficient functions obtained after removing each of the 231 SNPs in turn. The shaded regions are influence envelopes and do not represent confidence intervals.

functions. White blood cell count also shows comparatively greater sensitivity to omission of several correlated hematological biomarkers. In contrast, many other biomarker omissions produce substantially smaller changes. These results indicate that some exposure-specific coefficient functions depend on the composition of the jointly modeled biomarker set, as expected for conditional effects in a multivariable framework.

4. Complementary pleiotropy-robust multivariable MR analysis

As a complementary sensitivity analysis, we construct a conventional scalar multivariable MR analysis using the 13 biomarker measurements at visit V08. This analysis includes 580 individuals with complete V08 biomarker measurements and genotype data. We first evaluate

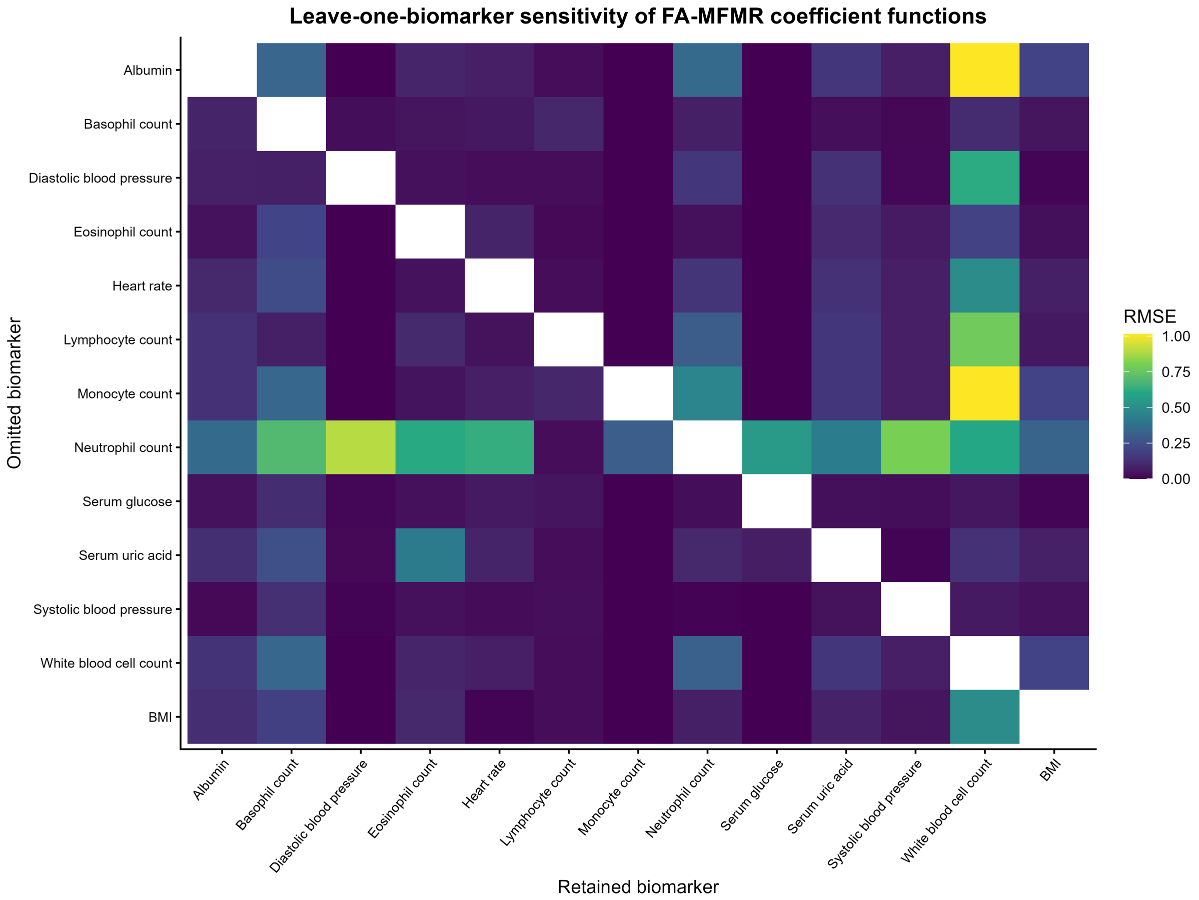

Figure S19: **Leave-one-biomarker sensitivity of the FA-MFMR coefficient functions.** Each row corresponds to the biomarker omitted from the multivariable model together with its assigned instruments, and each column corresponds to a retained biomarker. Cell values represent the RMSE between the reduced-model and full-model coefficient functions. Larger values indicate greater sensitivity of the retained coefficient function to omission of the corresponding biomarker.

conditional instrument strength and then apply Q-heterogeneity minimization to obtain multivariable estimates that are more robust to heterogeneity and pleiotropic instruments.

The conditional F-statistics range from approximately 0.92 to 1.28 across the 13 biomarkers, indicating severe conditional weak-instrument strength in the V08 scalar representation (Figure S20A). Accordingly, the scalar MVMR analysis is considered inconclusive and is not used to validate or corroborate the longitudinal FA-MFMR findings.

Although the Q-heterogeneity-minimization estimates for neutrophil count and white blood cell count have directions similar to those of the corresponding FA-MFMR summaries (Figure S20B), this directional agreement is not interpreted as supporting evidence because of the severe conditional weak-instrument problem and the different scalar estimand. Other scalar estimates also do not uniformly reproduce the longitudinal patterns. We therefore retain this scalar MVMR

analysis only as an exploratory sensitivity analysis.

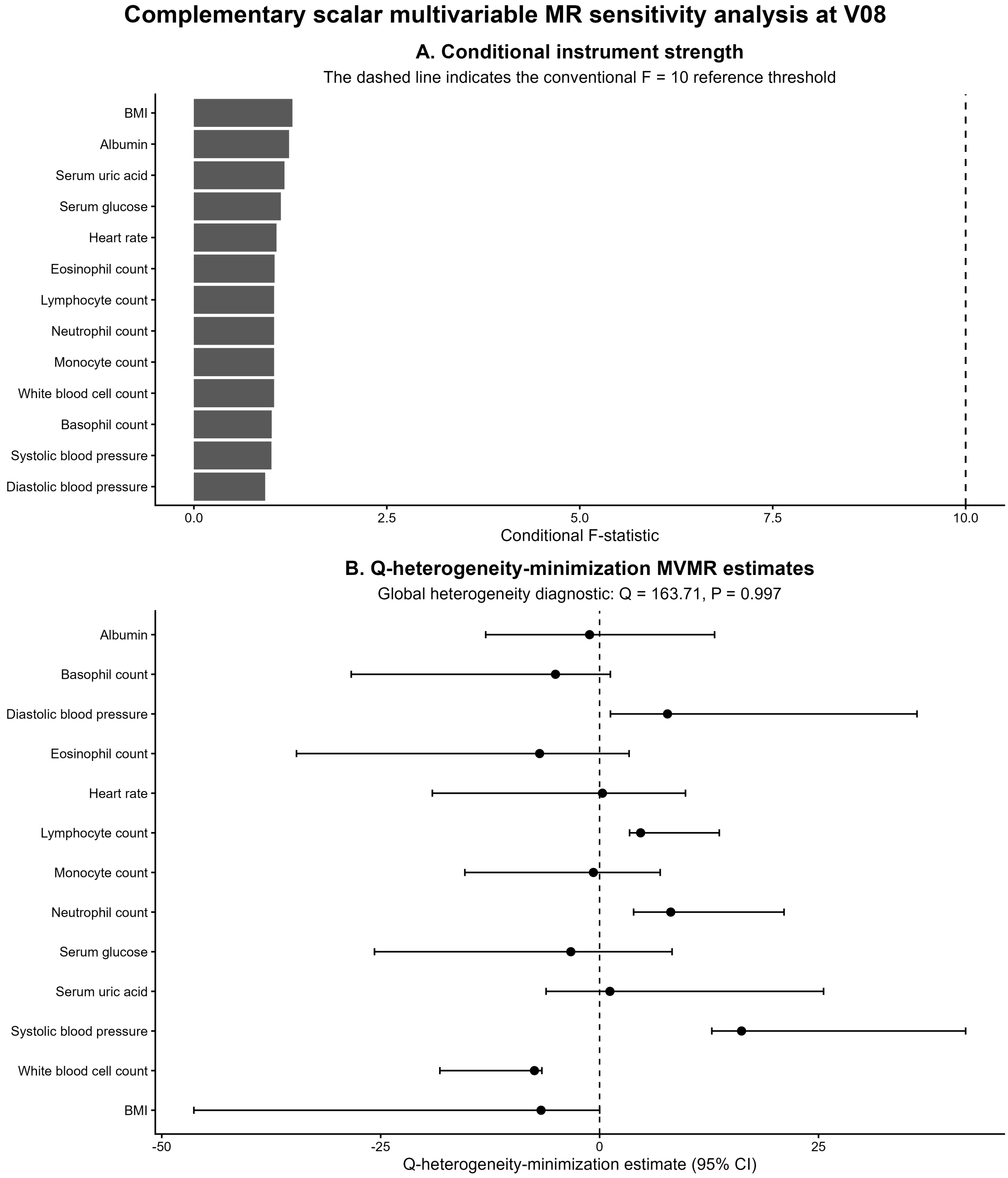

Figure S20: **Complementary scalar multivariable MR sensitivity analysis at visit V08.** **(A)** Conditional F-statistics for the 13 biomarkers. The dashed line denotes the conventional $F = 10$ reference threshold. **(B)** Q-heterogeneity-minimization multivariable MR estimates with 95% confidence intervals. The vertical dashed line denotes zero.

S2.7: Longitudinal age coverage and cohort sensitivity analyses

The primary PPMI analysis uses chronological age as the functional time index and relates the longitudinal biomarker trajectories to the subject-specific terminal NP3TOT outcome, as described in the main text. Here, we provide additional diagnostics for longitudinal age coverage and the distribution of subject-specific terminal outcome ages, clarify the interpretation of reconstructed trajectory segments beyond an individual' s outcome age, and evaluate the sensitivity of the estimated functional effects to an age-70 landmark design and restriction to participants with PD.

1. Within-participant age coverage, local cross-subject support, and terminal outcome ages

Because the common functional domain spans ages 50-80 years whereas individual participants are followed for substantially shorter periods, we quantify the amount of chronological-age coverage contributed directly by each participant. For participant i, we define the observed age span as

$$S_i = \max_j (t_{ij}) - \min_j (t_{ij}),$$

where t_{ij} denotes the chronological age at the j-th biomarker measurement. Relative coverage of the common 50–80-year domain is defined as

$$C_i = \frac{S_i}{30} \times 100\%.$$

In the final analytic cohort of 703 participants, the median within-participant chronological-age span is 5.1 years (IQR, 4.1-6.1 years), corresponding to a median direct coverage of 17.0% of the 30-year functional domain. Thus, the 50–80-year functional interval does not represent 30 years of within-participant follow-up. Instead, the common domain is supported by staggered longitudinal segments contributed by participants observed at different chronological ages.

We additionally quantify the empirical cross-subject support available at each chronological age. For each biomarker and age t, we count the number of unique participants with at least one observation within a ± 1.5-year neighborhood of t, corresponding to the mean-smoothing bandwidth used in the sparse functional analysis. Across the 50-80-year domain, the median age-specific support across the 13 biomarkers is 188 participants, with age-specific median support ranging from 57 to 261 participants. These diagnostics indicate that although direct within-

participant coverage is relatively short, each local portion of the common age domain is supported by measurements from multiple participants (Figure S21A-B).

We additionally examine the distribution of the subject-specific terminal outcome ages. For participant i, let T_i denote the chronological age at the selected terminal NP3TOT assessment, defined as the available NP3TOT assessment closest to age 80. In the final analytic cohort of 703 participants, T_i has a median of 71.8 years (IQR, 66.0–76.9 years) and ranges from 51.0 to 81.4 years (Figure S21C). Thus, whereas the theoretical formulation assumes a common terminal time, the PPMI application uses subject-specific terminal outcome ages determined by the available follow-up.

Only biomarker measurements observed before T_i are used as observed longitudinal exposure data for participant i. Sparse functional reconstruction nevertheless represents the latent biomarker trajectories over the common chronological-age domain of 50–80 years. Consequently, for $t > T_i$, the reconstructed value $\widehat{X}_i^{(k)}(t)$ represents a model-based continuation informed by the participant's available pre-outcome measurements and the estimated population mean and covariance structure. Such reconstructed values do not represent observed pre-outcome biomarker histories for that participant and are not assigned a subject-specific temporal causal interpretation.

2. Landmark and PD-only sensitivity analyses

We perform two additional sensitivity analyses to evaluate the stability of the fitted functional effects under stricter temporal and cohort restrictions.

For the landmark analysis, chronological age 70 years is used as the landmark. Only biomarker measurements observed before age 70 contribute to the exposure trajectories, whereas the selected terminal NP3TOT assessment is required to occur after age 70. This design ensures that the analyzed exposure age domain precedes the outcome assessment for every included participant and therefore provides a stricter temporal-ordering sensitivity analysis. Participants are additionally required to satisfy the same longitudinal completeness criteria used in the primary analysis, yielding 214 eligible participants.

The primary analysis contains 231 genetic instruments. Because the first-stage regressions in FA-MFMR jointly regress the estimated factor and idiosyncratic components on the instrument matrix, the original 231-instrument specification is not estimable in the landmark sample, for which $n = 214 < J = 231$. We therefore construct a reduced instrument set using only

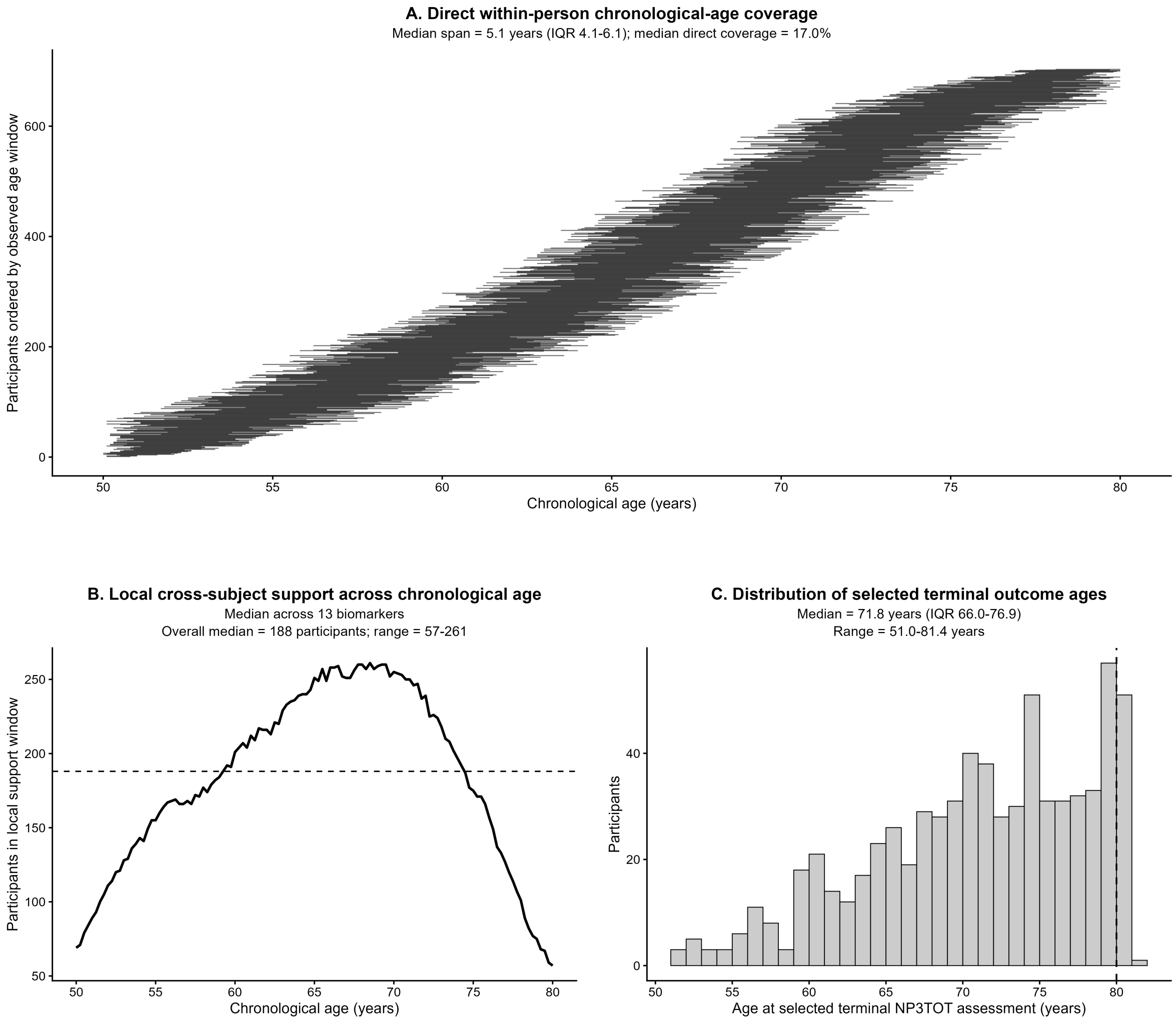

Figure S21: **Within-participant chronological-age coverage, local cross-subject support, and terminal outcome ages. (A)** Direct within-participant chronological-age coverage in the 703-participant analytic cohort. Each horizontal segment represents the interval between the earliest and latest repeated biomarker measurements for one participant, ordered by observed age window. The median within-participant age span is 5.1 years (IQR, 4.1–6.1 years), corresponding to a median direct coverage of 17.0% of the common 50–80-year age domain. **(B)** Local cross-subject support across chronological age. At each age, the curve shows the median across the 13 biomarkers of the number of unique participants contributing at least one observation within a ±1.5-year neighborhood. The overall median age-specific support is 188 participants, with an age-specific range of 57–261 participants. **(C)** Distribution of chronological ages at the selected terminal NP3TOT assessments used in the primary analysis. The median terminal outcome age is 71.8 years (IQR, 66.0–76.9 years), with a range of 51.0–81.4 years. The dashed vertical line marks the target age of 80 years.

SNP-biomarker information and without using NP3TOT or the estimated coefficient functions. Among the original instruments assigned to each biomarker, we evaluate the SNP-biomarker association at PPMI visit V08 and consider the pre-specified thresholds $0.025, 0.010, 0.005$. We select the most permissive threshold that retains at least one instrument for each of the 13 biomarkers while keeping the total number of instruments below 70% of the expected landmark

sample size. This procedure selects $p_{\text{V08}} < 0.025$, yielding 105 instruments while retaining representation of all 13 biomarkers. The same 105-instrument set is applied to the full cohort as a matched diagnostic comparator and to the age-70 landmark cohort.

For the PD-only sensitivity analysis, we restrict the cohort to participants enrolled in the PD or genetic-PD categories, yielding 413 participants. Because this sample remains larger than the original 231 -instrument set, all 231 instruments used in the primary analysis are retained.

We quantify differences between the sensitivity and primary fits using the biomarker-specific functional root mean squared discrepancy. For biomarker g,

$$\text{RMSE}_g = \left[\frac{1}{|\mathcal{T}_g|}\int_{\mathcal{T}_g}\left\{\widehat{\beta}_g^{\text{sens}}(t) - \widehat{\beta}_g^{\text{pri}}(t)\right\}^2 dt\right]^{1/2},$$

where $\mathcal{T}_g$ denotes the common age domain of the two fitted coefficient functions. The landmark analysis is compared with the primary analysis over ages 50-70 years, whereas the PD-only analysis is compared over ages 50-80 years. We further summarize the overall discrepancy across all 13 biomarkers by

$$\text{RMSE}_{\text{total}} = \left[\frac{\sum_{g=1}^{13}\int_{\mathcal{T}_g}\left\{\widehat{\beta}_g^{\text{sens}}(t) - \widehat{\beta}_g^{\text{pri}}(t)\right\}^2 dt}{\sum_{g=1}^{13}|\mathcal{T}_g|}\right]^{1/2}.$$

A value of zero indicates identical fitted coefficient functions, whereas larger values indicate greater discrepancy. These quantities describe differences between fitted functional-effect systems.

The total functional RMSE relative to the primary FA-MFMR analysis is 0.428 for the age-70 landmark analysis and 0.331 for the PD-only analysis. The discrepancies are heterogeneous across biomarkers rather than uniformly distributed across the 13 longitudinal exposures. In the landmark analysis, the largest biomarker-specific RMSE occurs for white blood cell count (1.35), followed by neutrophil count (0.538) and basophil count (0.344). The corresponding values in the PD-only analysis are 1.01, 0.437, and 0.330 , respectively. In both sensitivity analyses, 10 of the 13 biomarkers have functional RMSE values below 0.25 (Figure S22; Table S2).

Because the landmark analysis requires a reduced instrument set, we additionally quantify the discrepancy associated with this reduction. Applying the 105-instrument set to the full 703-participant cohort yields a total functional RMSE of 0.308 relative to the original 231-instrument primary analysis. The subsequent restriction from the full 105-instrument cohort to

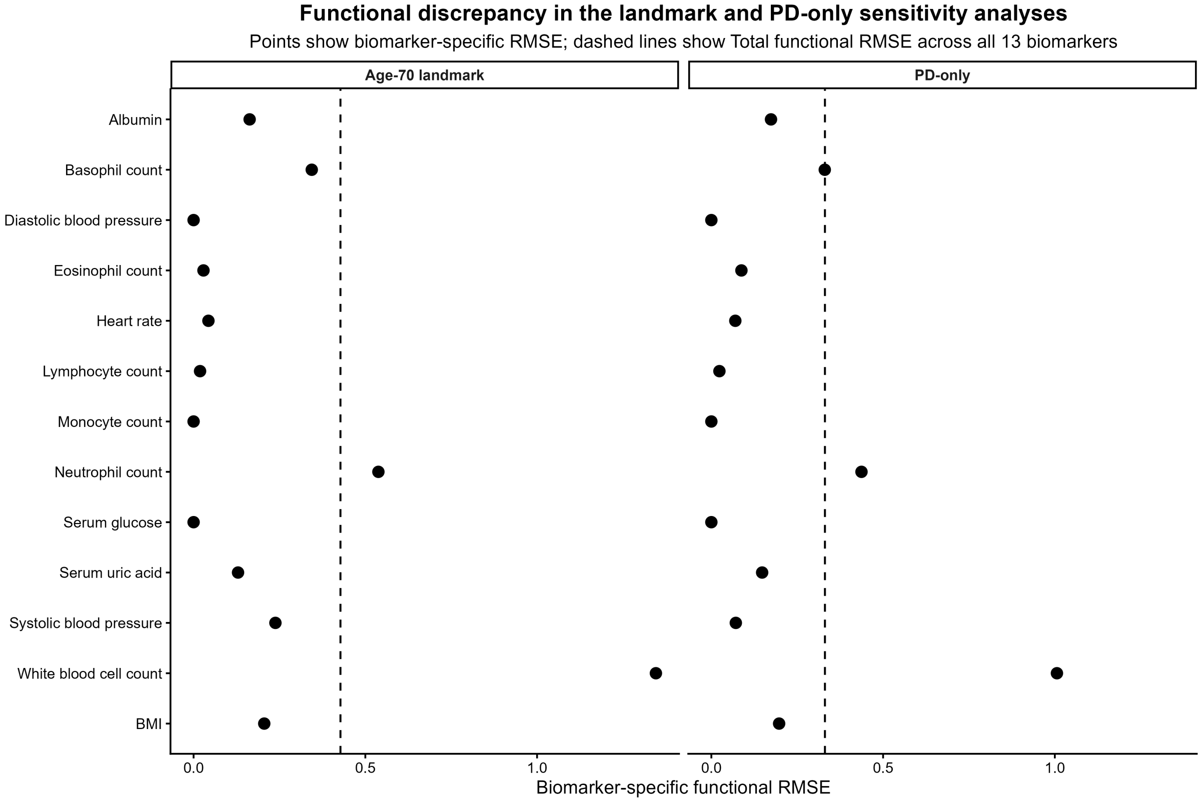

Figure S22: Biomarker-specific functional RMSE values quantify the discrepancy between the sensitivity-analysis and primary FA-MFMR coefficient functions over their common chronological-age domains. The left panel shows the age-70 landmark analysis and the right panel shows the PD-only analysis. Points represent biomarker-specific functional RMSE values. Dashed vertical lines indicate the total functional RMSE across all 13 biomarkers, equal to 0.428 for the landmark analysis and 0.331 for the PD-only analysis. Smaller values indicate closer agreement with the primary fitted coefficient function.

the age-70 landmark sample yields a total functional RMSE of 0.230 relative to the matched 105-instrument full-cohort analysis. Thus, the overall landmark discrepancy reflects both the necessary reduction of the instrument set and the additional temporal and sample restriction imposed by the landmark design.

Overall, the sensitivity-analysis discrepancies are concentrated primarily in white blood cell count and, to a lesser extent, neutrophil and basophil counts, whereas most of the remaining biomarkers show substantially smaller functional discrepancies.

Table S2: Biomarker-specific functional RMSE in the landmark and PD-only sensitivity analyses

Biomarker	Age-70 landmark	PD-only
Albumin	0.163	0.174
Basophil count	0.344	0.330
Diastolic blood pressure	0.000	0.000
Eosinophil count	0.029	0.088
Heart rate	0.043	0.070
Lymphocyte count	0.019	0.024
Monocyte count	0.000	0.000
Neutrophil count	0.538	0.437
Serum glucose	0.000	0.000
Serum uric acid	0.129	0.148
Systolic blood pressure	0.238	0.071
White blood cell count	1.350	1.010
BMI	0.206	0.197
**Total functional RMSE**	**0.428**	**0.331**

S2.8: Additional simulation settings beyond the baseline design

To further examine the performance of FA-MFMR beyond the baseline data-generating mechanism, we consider additional simulation settings involving departures in the factor structure, causal-effect sparsity, instrument mechanism, first-stage specification, and longitudinal observation process. We also include a PPMI-calibrated setting that more closely reflects the dimensionality and sparse longitudinal sampling of the real-data analysis. Unless otherwise stated, all components of the data-generating mechanism and evaluation procedure remain the same as in the main simulation study.

For the synthetic stress-test settings reported below, we consider $K = 10$ longitudinal exposures with sample sizes $n = 1000$ and $n = 2000, J = 100$ genetic instruments, and $r = 3$ latent factors whenever a factor structure is present. Each setting is repeated 100 times. The same six methods and the same Total Error criterion as in the main simulation study are used throughout.

1. Departures from the exposure and factor structure

We first consider three modifications of the exposure and factor structure.

- **No factor structure.**

The common-factor component is removed by setting $r = 0$, so that the score matrix is generated entirely from the idiosyncratic component, $A = U$. All remaining components of the data-generating mechanism are unchanged. FA-MFMR continues to estimate the factor

dimension using its usual data-driven rule, whereas oracle-FA-MFMR is supplied with the true value $r = 0$. This setting evaluates whether factor augmentation causes substantial deterioration when the assumed low-dimensional common structure is absent.

- **Dense exposure effects.**

The baseline simulations contain four nonzero exposure-specific coefficient functions. To reduce the degree of sparsity, we instead specify eight of the ten coefficient functions as nonzero when $K = 10$. When $K = 5$, all five coefficient functions are nonzero. The nonzero functions cycle through the same linear, quadratic, sinusoidal, and Gaussian-shaped temporal patterns used in the baseline design. This setting evaluates whether the relative performance of FA-MFMR depends strongly on a sparse causal-effect architecture.

- **Causal signal concentrated in the common-factor space.**

We construct a deliberately challenging setting in which the score-level causal coefficient direction is contained in the generating factor-loading space. Let $\boldsymbol{H}_0$ denote the score-level coefficient vector corresponding to the true functional effects and let $P_{\boldsymbol{\Lambda}}$ denote projection onto the generating loading space. The loading matrix is constructed so that its first loading direction is aligned with $\boldsymbol{H}_0$, giving

$$\rho_{\boldsymbol{F}} = \frac{\|P_{\boldsymbol{\Lambda}}\boldsymbol{H}_0\|_2^2}{\|\boldsymbol{H}_0\|_2^2} \approx 1.$$

The other loading directions are constructed orthogonally to this direction. Thus, most of the causal coefficient direction lies in the common-factor space, providing an extreme stress test for post-factor identification. This setting complements the graded alignment analysis in Section S2.5, which examines the relationship between common-factor alignment and the amount of residual information available after factor adjustment.

Figure S23 shows that FA-MFMR and oracle-FA-MFMR retain substantially lower Total Error than the non-factor-augmented competitors when the common-factor structure is absent. A similar ordering persists under dense exposure effects, indicating that the advantage of FA-MFMR is not restricted to the sparse four-active-exposure design used in the baseline simulations. Increasing the sample size from $n = 1000$ to $n = 2000$ generally reduces the estimation error while preserving the relative ordering of the methods.

The pattern changes when the causal coefficient direction is concentrated in the common-factor space. In this setting, pMFMR attains slightly lower Total Error than the factor-

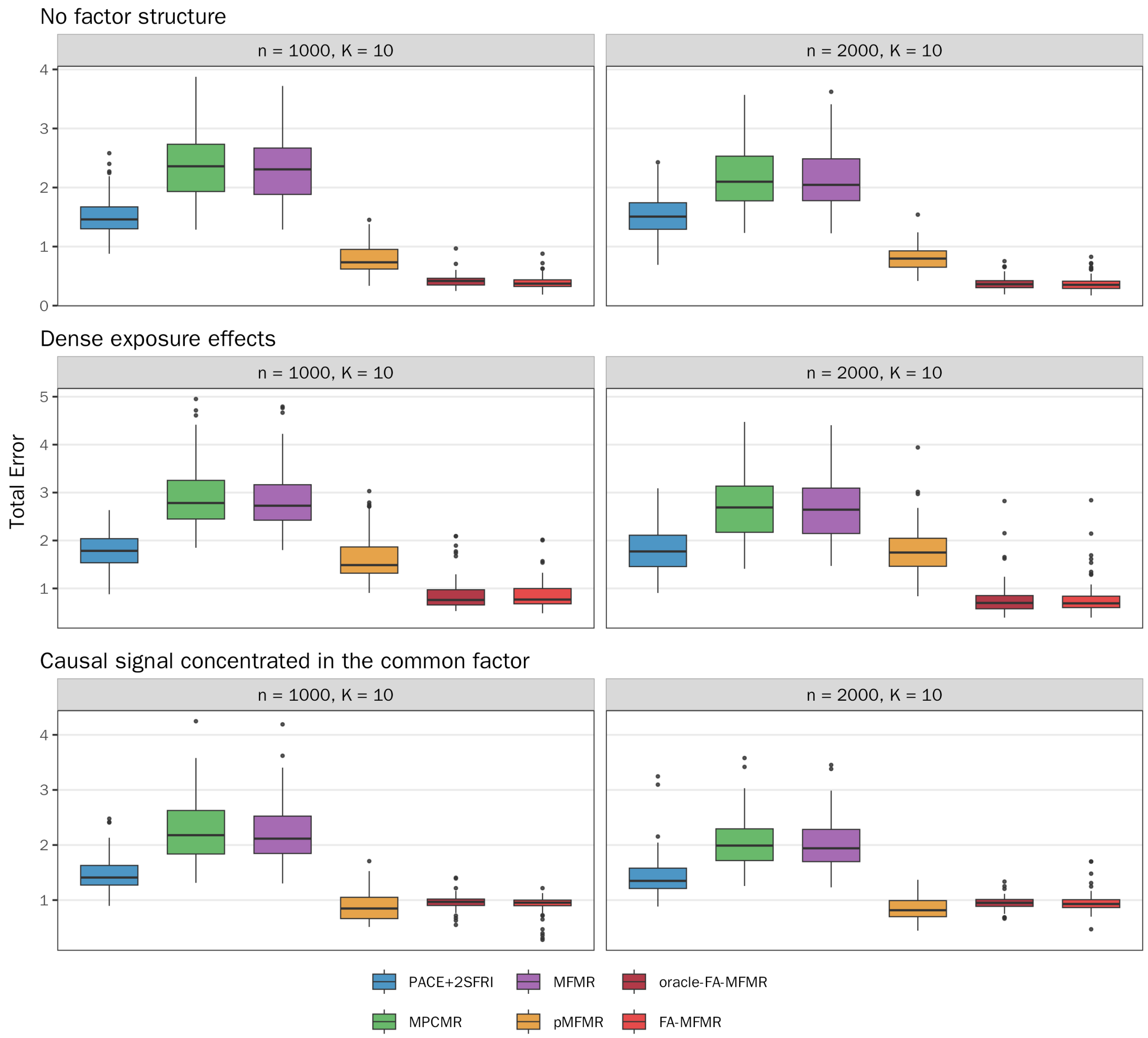

Figure S23: **Additional simulation settings involving the exposure and factor structure.** Total Error for PACE+2SFRI, MPCMR, MFMR, pMFMR, oracle-FA-MFMR, and FA-MFMR under the absence of a common-factor structure, dense exposure effects, and a causal coefficient direction concentrated in the common-factor space. Results are shown for $n = 1000$ and $n = 2000$ with $K = 10$. Each box summarizes 100 simulation replicates.

augmented estimators, while FA-MFMR and oracle-FA-MFMR remain close to one another. This behavior is consistent with the identification discussion in Section S2.5: when most of the causal direction lies in the factor space, factor residualization leaves less idiosyncratic variation for identifying exposure-specific effects. Thus, this setting illustrates an expected limitation rather than a failure of factor-number estimation.

2. Departures from the instrument and first-stage assumptions

We next consider four settings that modify the instrument or first-stage structure.

- **Weak instruments.**

The genetic-effect matrices in both first-stage equations are scaled by 0.01,

$$\mathbf{\Pi}_{\boldsymbol{F}}^{\text{weak}} = 0.01\mathbf{\Pi}_{\boldsymbol{F}}, \quad \mathbf{\Pi}_{\boldsymbol{U}}^{\text{weak}} = 0.01\mathbf{\Pi}_{\boldsymbol{U}},$$

while the first-stage noise distributions remain unchanged. This substantially reduces the genetic contribution to variation in the functional scores.

- **Horizontal pleiotropy and invalid instruments.**

We randomly designate 20% of the genetic instruments as invalid and add a direct SNP-to-outcome component. Let $\mathcal{I}_{\text{inv}}$ denote the set of invalid instruments and define

$$Q_i = \sum_{j \in \mathcal{I}_{\text{inv}}} \alpha_j Z_{ij}, \quad \alpha_j \sim N(0,1).$$

The standardized direct-effect component is scaled to have standard deviation equal to 50% of the standard deviation of the simulated causal component and is added directly to the outcome. Thus, the setting represents balanced horizontal pleiotropy and deliberately violates the exclusion restriction.

- **LD-correlated instruments.**

Instead of generating the SNPs independently, we introduce an AR(1)-type LD structure through a Gaussian-copula construction,

$$\text{Cor}\left(L_j, L_{j'}\right) = 0.3^{|j-j'|},$$

where L_j denotes the latent Gaussian variable for SNP j. Two correlated Bernoulli haplotypes are generated using SNP-specific allele frequencies and are summed to obtain genotype dosages in $\{0, 1, 2\}$, after which the genotype matrix is standardized as in the baseline design.

- **Nonlinear first stage.**

To evaluate sensitivity to misspecification of the linear SNP–exposure relationship, we augment both first-stage equations with a standardized quadratic component,

$$\boldsymbol{F} = \boldsymbol{Z}\mathbf{\Pi}_{\boldsymbol{F}} + 0.75\boldsymbol{Z}^{(2)}\mathbf{\Pi}_{\boldsymbol{F},2} + \boldsymbol{E}_{\boldsymbol{F}},$$

and

$$\boldsymbol{U} = \boldsymbol{Z}\boldsymbol{\Pi}_{\boldsymbol{U}} + 0.75\boldsymbol{Z}^{(2)}\boldsymbol{\Pi}_{\boldsymbol{U},2} + \boldsymbol{E}_{\boldsymbol{U}},$$

where $\boldsymbol{Z}^{(2)}$ denotes the column-standardized elementwise square of $\boldsymbol{Z}$. The fitted methods continue to use the linear instrument matrix $\boldsymbol{Z}$, so that the quadratic component represents deliberate first-stage misspecification.

As shown in Figure S24, the relative performance of the six methods is broadly preserved under weak instruments, LD-correlated SNPs, and nonlinear first-stage relationships. In all three settings, FA-MFMR and oracle-FA-MFMR yield the smallest Total Error, followed by pMFMR, whereas PACE+2SFRI, MFMR, and MPCMR exhibit noticeably larger errors. Increasing the sample size from $n = 1000$ to $n = 2000$ generally reduces the estimation error for these three departures, while leaving the relative ordering of the methods largely unchanged. The similarity between FA-MFMR and oracle-FA-MFMR also suggests that data-driven factor-number selection contributes little additional loss in these settings.

The horizontal-pleiotropy setting produces a qualitatively different pattern. When 20% of the SNPs have direct effects on the outcome, Total Error increases substantially for all six methods, and the separation between FA-MFMR, oracle-FA-MFMR, and pMFMR largely disappears. PACE+2SFRI shows the smallest median error in this setting, although all methods perform considerably worse than under the other first-stage perturbations. Moreover, increasing the sample size does not alleviate the deterioration caused by pleiotropy; if anything, the error distributions remain comparably large or shift upward at $n = 2000$. This pattern is consistent with a persistent bias induced by violation of the exclusion restriction rather than a finite-sample variance problem. Because factor augmentation is designed to account for dependence among the longitudinal exposures, rather than direct SNP-to-outcome pathways, it does not protect against substantial horizontal pleiotropy. Thus, this experiment identifies an important boundary of FA-MFMR: the method remains comparatively stable under weak instruments, moderate LD, and nonlinear first-stage misspecification, but its advantage is attenuated when instrument validity itself is violated.

3. Informative visit patterns

The baseline simulations generate observation times independently of the underlying exposure trajectories. We additionally consider an informative observation mechanism in which both

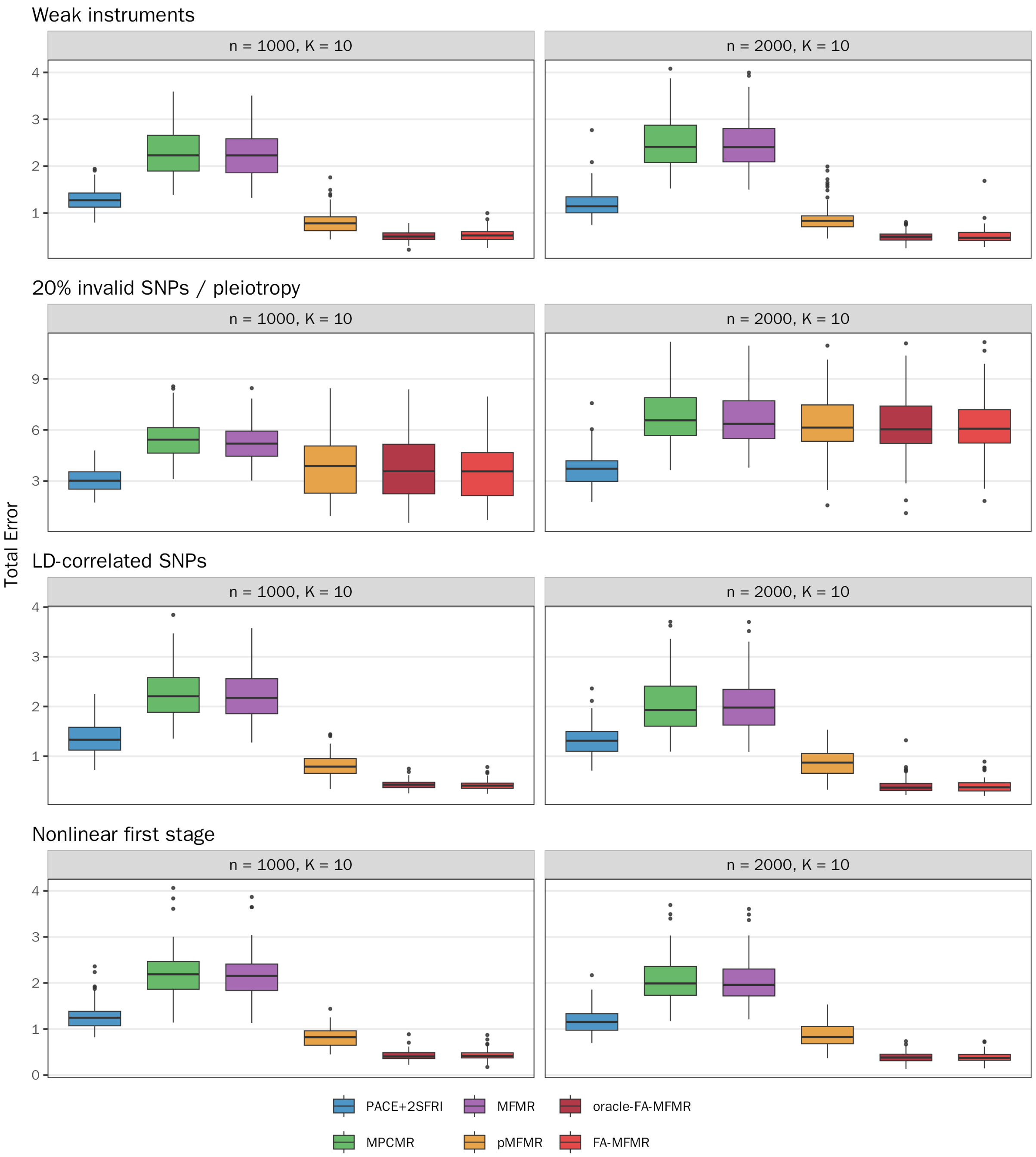

Figure S24: **Additional simulation settings involving the instrument and first-stage mechanisms.** Total Error under weak instruments, horizontal pleiotropy with 20% invalid SNPs, LD-correlated genetic instruments, and a nonlinear SNP-exposure first stage. Results are shown for $n = 1000$ and $n = 2000$ with $K = 10$ and $r = 3$. Each box summarizes 100 simulation replicates.

the number and timing of longitudinal measurements depend on the first exposure trajectory.

Let S_i denote the standardized subject-level average of the first exposure trajectory. The number of repeated measurements depends on

$$p_i = \text{logit}^{-1}(0.8S_i),$$

subject to the same 5-15 visit range used in the baseline simulation. Conditional on the number of measurements, the probability of observing subject i at time t is weighted according to

$$w_i(t) \propto w_0(t) \exp\left\{0.8\widetilde{X}_i^{(1)}(t)\right\},$$

where $w_0(t)$ denotes the baseline observation-time weight and $\widetilde{X}_i^{(1)}(t)$ is the standardized first exposure trajectory. Consequently, both visit frequency and observation timing are associated with the underlying exposure process.

Figure S25 shows that the factor-augmented estimators retain the lowest Total Error under this informative observation mechanism. FA-MFMR and oracle-FA-MFMR produce closely similar error distributions, followed by pMFMR, whereas PACE+2SFRI, MFMR, and MPCMR show substantially larger errors. The same qualitative ordering is observed at $n = 1000$ and $n = 2000$.

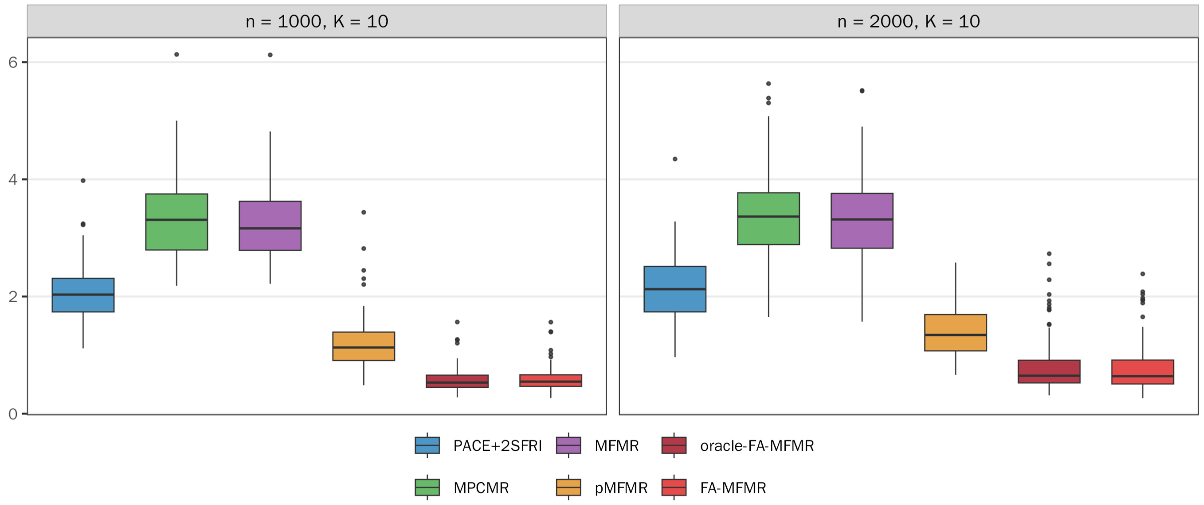

Figure S25: **Additional simulation under informative visit patterns.** Total Error when the number and timing of longitudinal measurements depend on the underlying first exposure trajectory. Results are shown for $n = 1000$ and $n = 2000$ with $K = 10$ and $r = 3$. Each box summarizes 100 simulation replicates.

4. PPMI-calibrated simulation

We further construct a simulation setting that more closely reflects the scale and longitudinal sparsity of the PPMI application. This setting uses

$$n = 703, \quad K = 13, \quad J = 231, \quad r = 3,$$

with the functional domain defined over chronological ages 50-80 years. Each participant has 5, 6, or 7 repeated measurements with probabilities $0.25, 0.50$, and 0.25, respectively, producing a sparse visit distribution centered at six measurements per participant.

The 231 genetic instruments are allocated across the 13 biomarkers according to the biomarker-specific instrument counts used in the PPMI analysis. For each biomarker, only its corresponding SNP block contributes to the exposure-specific idiosyncratic score block. The remaining components of the data-generating mechanism follow the baseline simulation design. Total Error in this setting is evaluated at ages 55, 60, 70, and 75.

Figure S26 shows that FA-MFMR and oracle-FA-MFMR achieve the lowest Total Error in the PPMI-calibrated design, with very similar median and dispersion. pMFMR provides the next-best performance, whereas PACE+2SFRI, MFMR, and MPCMR have larger estimation errors. Thus, the favorable performance of FA-MFMR persists in a design with the sample size, exposure dimension, sparse visits, and biomarker-specific instrument allocation chosen to resemble the real-data application.

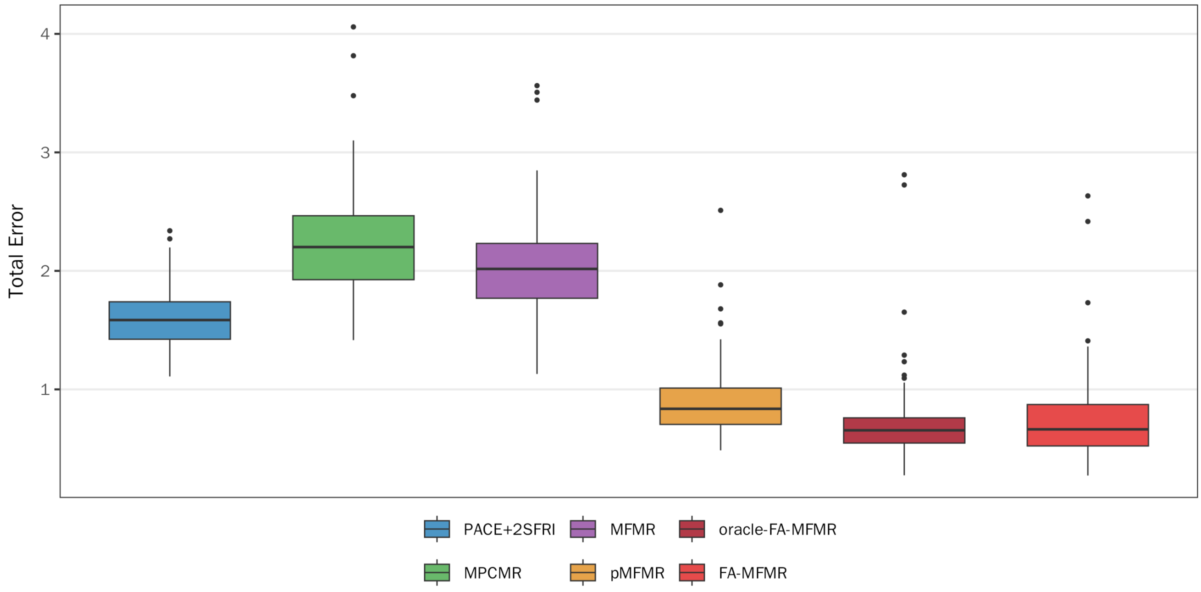

Figure S26: **PPMI-calibrated simulation.** Total Error under an application-calibrated design with $n = 703$, $K = 13$ longitudinal exposures, $J = 231$ genetic instruments, and $r = 3$ latent factors, together with sparse repeated measurements over ages 50–80 years and biomarker-specific instrument counts corresponding to the PPMI analysis. Each box summarizes 100 simulation replicates.

5. Sensitivity to factor-number misspecification

Finally, we examine the effect of moderate misspecification of the factor dimension used by FA-MFMR. Data are generated with $r_0 = 3$ and the same datasets are analyzed using four variants:

$$\text{oracle-FA-MFMR } (r = 2), \quad \text{oracle-FA-MFMR } (r = 3), \quad \text{FA-MFMR } (r = 4), \quad \text{FA-MFMR } (\hat{r})$$

where $\hat{r}$ is selected using the same data-driven criterion as in the main analysis. Thus, $r = 2$ represents moderate under-specification, $r = 4$ represents moderate over-specification, and oracle-FA-MFMR uses the true factor dimension.

Figure S27 shows substantial overlap among the four error distributions. The median Total Errors are highly similar for $r = 2$, the oracle value $r = 3, r = 4$, and the data-driven $\hat{r}$, at both $n = 1000$ and $n = 2000$. The distributions shift slightly downward with the larger sample size, but the relative similarity among the four specifications remains. These results indicate that, in this setting, FA-MFMR is relatively insensitive to moderate under- or over-specification of the factor number.

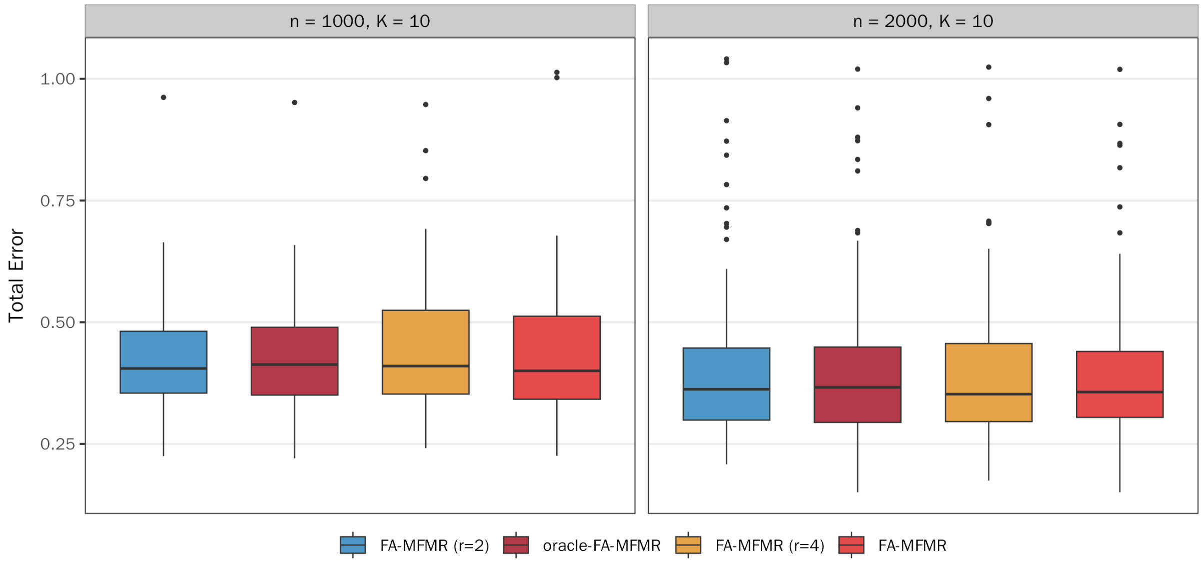

Figure S27: **Sensitivity to factor-number misspecification.** Total Error when data are generated with $r_0 = 3$ and FA-MFMR is fitted using $r = 2$, the oracle value $r = 3, r = 4$, or the data-driven estimate $\hat{r}$. Results are shown for $n = 1000$ and $n = 2000$ with $K = 10$. Each box summarizes 100 simulation replicates.

Taken together, these additional simulations show that the relative advantage of FA-MFMR persists under several departures from the baseline design, including the absence of a common-

factor structure, dense causal effects, weak instruments, LD-correlated variants, nonlinear first-stage relationships, informative observation patterns, and the PPMI-calibrated design. The simulations also identify two important limitations. Horizontal pleiotropy substantially degrades all MR-based estimators because factor augmentation does not address violations of the exclusion restriction, while concentrating the causal coefficient direction in the common-factor space reduces the benefit of factor residualization because less identifying variation remains in the idiosyncratic component. FA-MFMR is also relatively insensitive to moderate under- or over-specification of the factor dimension.

S2.9: Multiplicity-aware bootstrap uncertainty for PPMI coefficient functions and curve summaries

We further assess curve-level uncertainty using the $B = 500$ participant-level bootstrap resamples described in the main text. For biomarker g in bootstrap replicate b, the coefficient function is reconstructed using the replicate-specific FPCA basis as

$$\widehat{\beta}_g^{*(b)}(t) = \sum_{j=1}^{m_g^{*(b)}} \widehat{\eta}_{gj}^{*(b)} \widehat{\phi}_{gj}^{*(b)}(t),$$

The reconstructed curves are evaluated on a common grid over ages 50–80. Because the FPCA truncation, factor-number selection, and SCAD tuning and selection steps are repeated within the bootstrap refits, variability associated with these data-adaptive steps enters the resampling distribution.

1. Joint simultaneous confidence bands

The confidence bands in Figure 9 are pointwise. To account jointly for variation across biomarkers and age, we additionally construct studentized simultaneous confidence bands over all 13 biomarkers and the complete age domain.

For biomarker g, the bootstrap standard error at age t is

$$\widehat{s}_g(t) = \mathrm{sd}_b \left\{ \widehat{\beta}_g^{*(b)}(t) \right\}.$$

For bootstrap replicate b, we define the joint studentized maximum statistic as

$$M_b = \max_{g=1,\ldots,13} \sup_{t\in[50,80]} \left| \frac{\widehat{\beta}_g^{*(b)}(t) - \widehat{\beta}_g(t)}{\widehat{s}_g(t)} \right|.$$

Let $c_{0.95}$ denote the empirical 95th percentile of the bootstrap distribution of M_b. The joint 95% simultaneous confidence band for biomarker g is $\widehat{\beta}_g(t) \pm c_{0.95}\widehat{s}_g(t)$. Because the maximum statistic is evaluated jointly over all biomarkers and ages, the resulting bands account for multiplicity across both dimensions.

In the PPMI analysis, the joint critical value is $c_{0.95} = 8.945$. The simultaneous confidence bands include zero throughout ages 50-80 for all 13 biomarkers (Figure S28). Thus, although several localized departures are visible in the pointwise analysis, no positive or negative age interval remains separated from zero under the joint simultaneous analysis.

2. Global curve tests

We further assess each biomarker using the global null hypothesis

$$H_{0g} : \beta_g(t) = 0, \qquad \text{for all } t \in [50, 80].$$

The observed global statistic is

$$T_g = \sup_{t\in[50,80]} \left| \frac{\widehat{\beta}_g(t)}{\widehat{s}_g(t)} \right|.$$

For each bootstrap replicate and biomarker, the corresponding centered statistic is

$$T_g^{*(b)} = \sup_{t\in[50,80]} \left| \frac{\widehat{\beta}_g^{*(b)}(t) - \widehat{\beta}_g(t)}{\widehat{s}_g(t)} \right|.$$

To account for testing multiple biomarker-specific curves, we use the joint max-T bootstrap distribution. The multiplicity-adjusted p-value for biomarker g is obtained by comparing T_g with the bootstrap distribution of M_b. None of the 13 biomarkers has a joint max- T-adjusted p-value below 0.05 (Figure S29). The smallest adjusted p-value is observed for white blood cell count ($p_{\max} = 0.855$). These results are consistent with the joint simultaneous confidence bands.

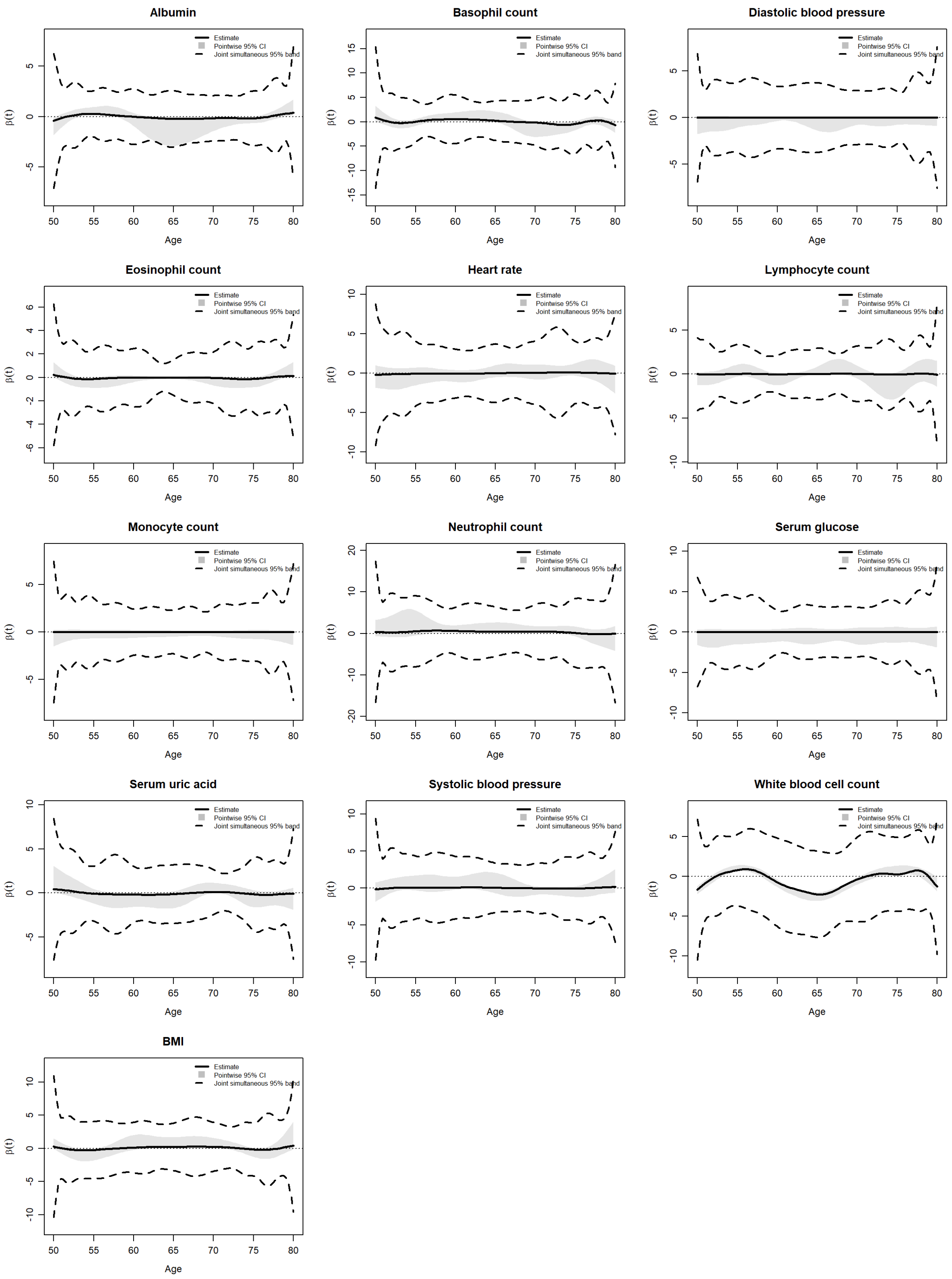

Figure S28: Pointwise and joint simultaneous confidence bands for the 13 PPMI biomarkers.

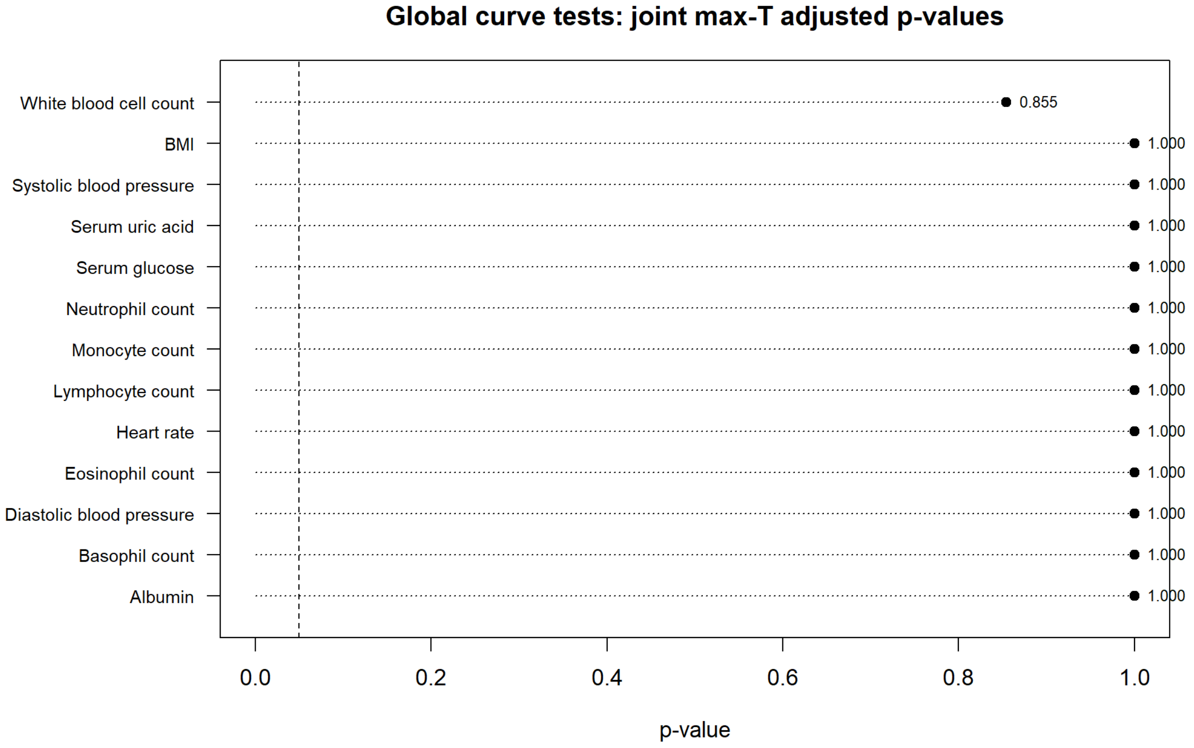

Figure S29: Multiplicity-adjusted global curve tests for the 13 PPMI biomarkers. Joint max-T adjusted bootstrap p-values are shown for the global hypotheses $H_{0g} : \beta_g(t) = 0$ for all $t \in [50, 80]$. The vertical reference line denotes $p = 0.05$.

3. Uncertainty for curve summaries

We also quantify uncertainty for the scalar summaries used to characterize the estimated coefficient functions in Figure 10. For biomarker g, the integrated effect over ages 50-80 is

$$I_g = \int_{50}^{80} \widehat{\beta}_g(t)dt.$$

The maximum and minimum effects are defined as

$$M_g^+ = \max_{t\in[50,80]} \widehat{\beta}_g(t), \quad M_g^- = \min_{t\in[50,80]} \widehat{\beta}_g(t),$$

and their corresponding ages are

$$t_g^+ = \arg\max_{t\in[50,80]} \widehat{\beta}_g(t), \quad t_g^- = \arg\min_{t\in[50,80]} \widehat{\beta}_g(t).$$

The proportion of the analyzed age domain with a positive estimated effect is defined as

$$P_g^+ = \frac{1}{30}\int_{50}^{80} \mathbf{1}\left\{\widehat{\beta}_g(t) > 0\right\} dt.$$

Each quantity is recalculated from the reconstructed coefficient function in every bootstrap replicate. The corresponding 95% confidence intervals are obtained from the 2.5th and 97.5th percentiles of the bootstrap distributions.

Figure S30 presents the point estimates and bootstrap 95% confidence intervals for the integrated effect, maximum effect, minimum effect, and proportion of positive ages. The integrated-effect intervals overlap zero for all biomarkers, while the remaining panels quantify the uncertainty associated with the extrema and directional persistence of the estimated coefficient functions.

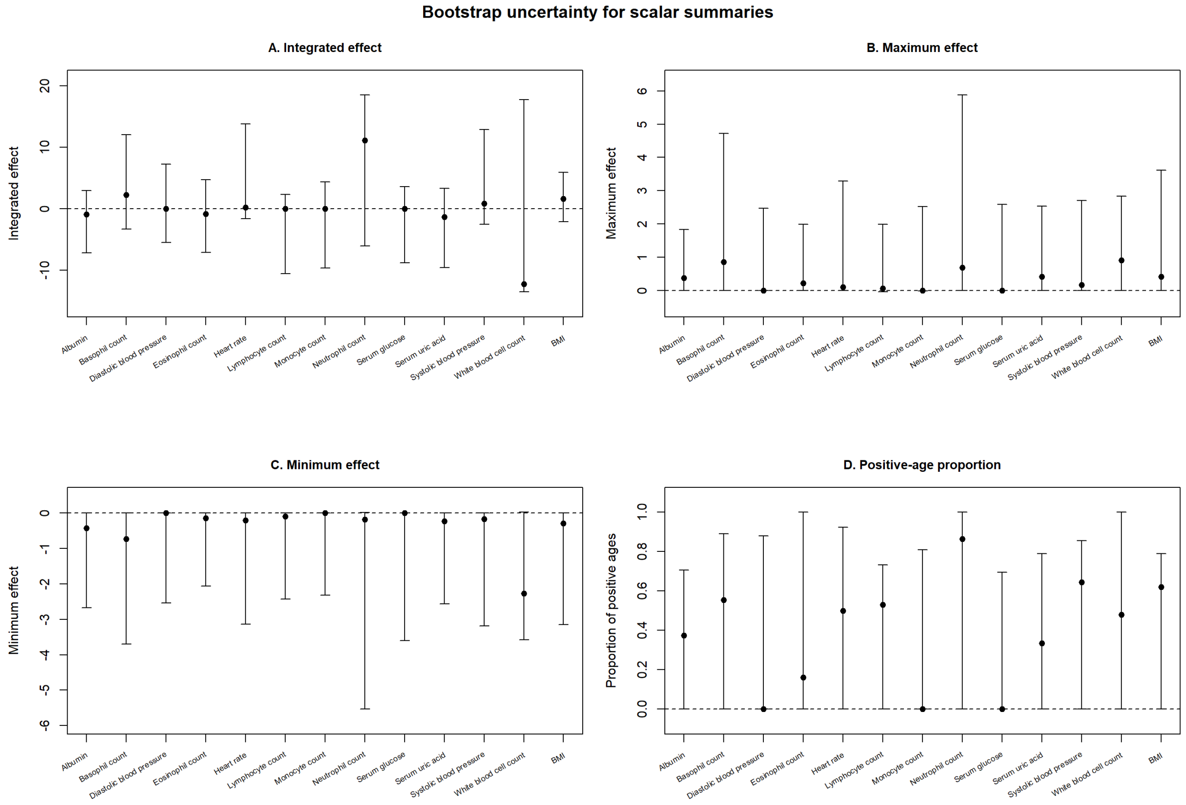

Figure S30: **Bootstrap uncertainty for scalar summaries of the PPMI coefficient functions.** Point estimates and 95% bootstrap confidence intervals are shown for **(A)** the integrated effect over ages 50–80, **(B)** the maximum effect, **(C)** the minimum effect, and **(D)** the proportion of ages with a positive estimated effect. All summaries are recalculated from the replicate-specific bootstrap coefficient functions.

Figure S31 presents the corresponding bootstrap uncertainty for the ages at which the

maximum and minimum effects occur. Together, Figures S30 and S31 complement the point estimates in Figure 10 by providing uncertainty measures for the principal curve-level summaries.

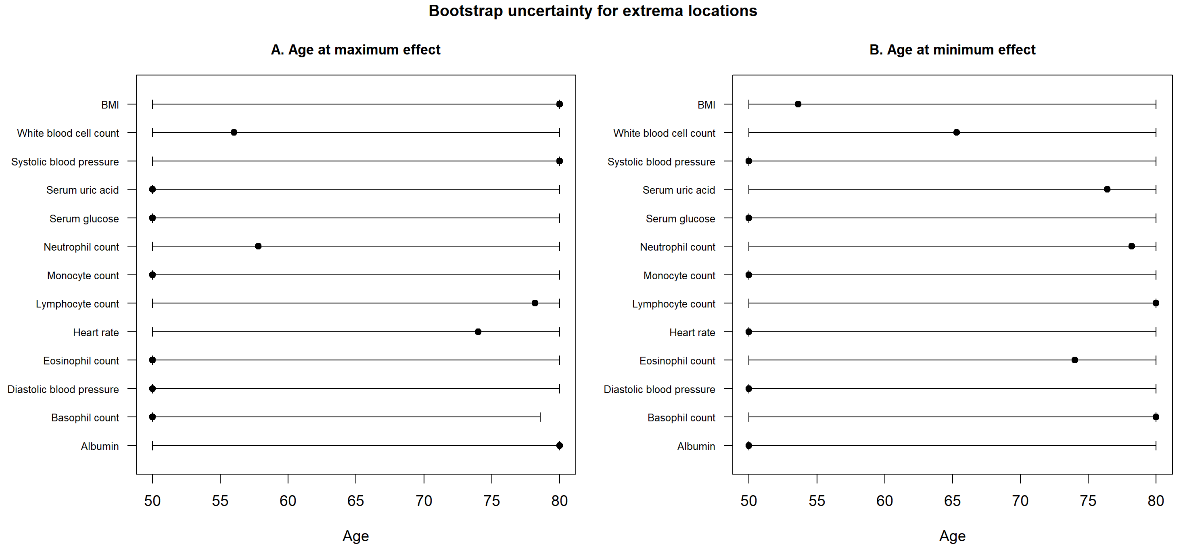

Figure S31: **Bootstrap uncertainty for the ages of the extrema.** Point estimates and 95% bootstrap confidence intervals are shown for **(A)** the age at maximum effect and **(B)** the age at minimum effect for each biomarker over the analyzed age range of 50–80 years.

S2.10: Sensitivity to instrument-selection criteria

To assess the sensitivity of FA-MFMR to instrument selection, we conduct complementary analyses in both the simulation study and the PPMI application. The simulation analysis varies the number of retained instruments according to their association strength with the estimated functional-score system, whereas the PPMI analysis varies the internal V08 SNP–biomarker association threshold used in the primary instrument-selection procedure. These analyses characterize the trade-off between retaining a larger instrument set and imposing more stringent first-stage relevance criteria.

1. Simulation sensitivity to instrument-selection stringency

We first evaluate instrument-selection sensitivity under the baseline simulation setting with $K = 10$ longitudinal exposures, $r = 3$ latent factors, $J = 100$ candidate SNPs, and sample sizes $n \in \{1000, 2000\}$. Each setting is repeated over 100 simulation replicates.

Within each replicate, the longitudinal data and PACE/FPCA representation are generated and estimated once and are held fixed across the instrument-selection settings. Let $\widehat{\boldsymbol{A}}$ denote the retained estimated FPCA-score matrix before IV projection. For candidate SNP j, we define

its empirical score-space association strength as

$$S_j = \left\| \widehat{\mathrm{Corr}} \left(Z_{\cdot j}, \widehat{\boldsymbol{A}} \right) \right\|_2 ,$$

where the columns of $\widehat{\boldsymbol{A}}$ and the SNP vector are standardized before calculating the correlations. Larger S_j indicates stronger overall association of SNP j with the retained functional-score system.

We compare three nested instrument sets: all 100 candidate SNPs, the 90 SNPs with the largest values of S_j, and the 80 SNPs with the largest values of S_j. For each instrument set, the IV projection and all subsequent instrument-dependent estimation steps are repeated. PACE+2SFRI, MPCMR, MFMR, pMFMR, oracle-FA-MFMR, and FA-MFMR are each refitted using the selected instruments. Thus, differences among the three settings reflect changes in the available instrument information.

Figure S32 shows that estimation error generally increases as the instrument set becomes more restrictive. The deterioration is most pronounced for MPCMR and MFMR, while PACE+2SFRI also exhibits increasing Total Error as the number of retained SNPs decreases. pMFMR shows a smaller increase but retains substantially larger error than the two factor-augmented estimators. In contrast, FA-MFMR and oracle-FA-MFMR consistently achieve the smallest Total Error across all three instrument sets and show comparatively limited deterioration as the number of retained instruments decreases. The same qualitative pattern is observed for both $n = 1000$ and $n = 2000$.

These results illustrate that selecting individually stronger instruments does not necessarily improve estimation of the complete multivariable functional system. More stringent selection increases the average strength of the retained instruments but simultaneously reduces the genetic information available to support the multiple retained FPCA-score directions. Instrument selection therefore involves a trade-off between individual instrument strength and joint coverage of the functional-score space. FA-MFMR retains its relative advantage over the competing methods across the instrument-selection settings considered here.

2. PPMI sensitivity to the internal instrument-selection threshold

The primary PPMI analysis uses an internal V08 SNP-biomarker association threshold of $p < 0.05$, resulting in 231 retained SNPs across the 13 biomarkers and a final analytic cohort of

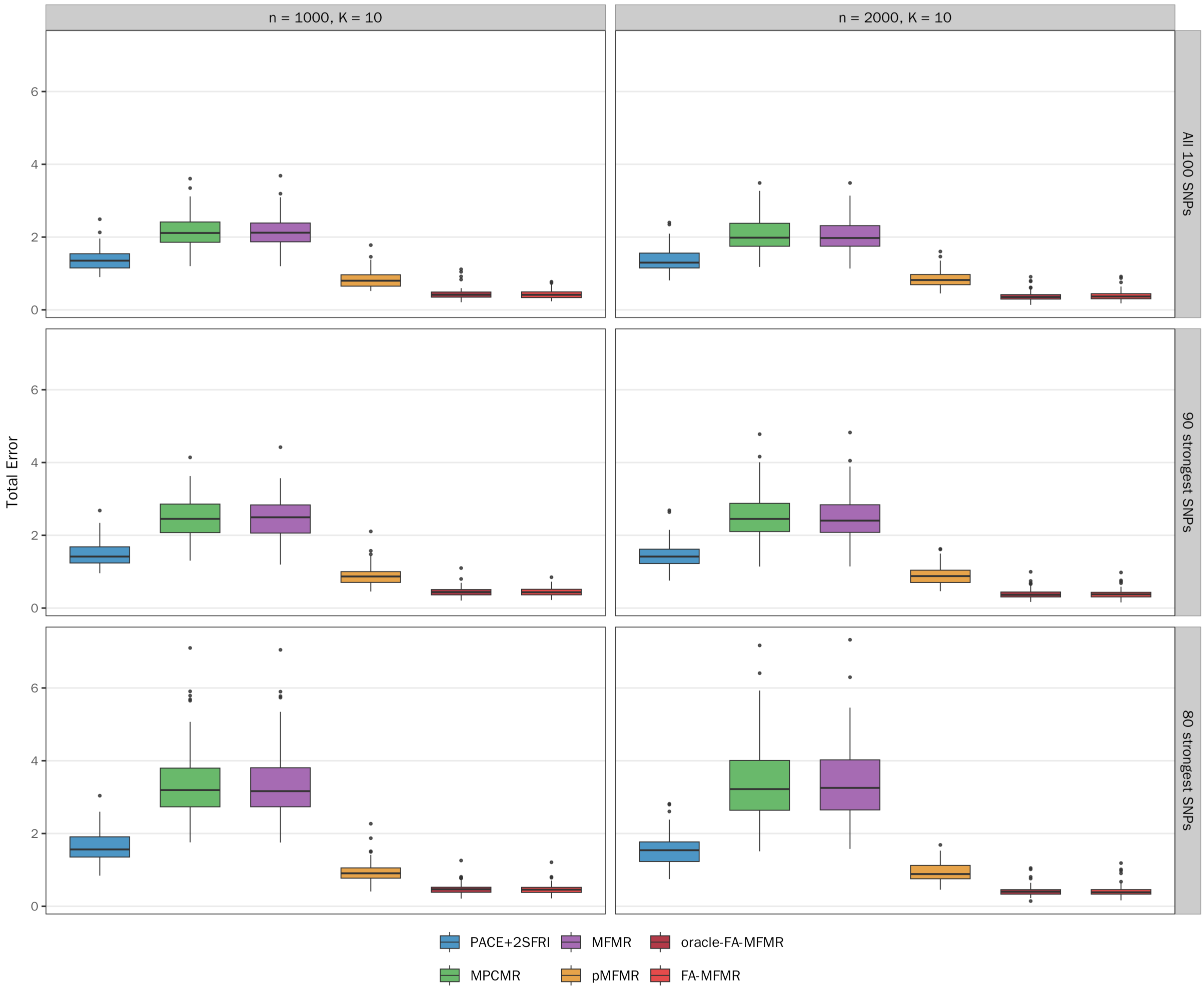

Figure S32: **Sensitivity to instrument-selection stringency in the simulation study.** Total Error distributions are shown for PACE+2SFRI, MPCMR, MFMR, pMFMR, oracle-FA-MFMR, and FA-MFMR using all 100 candidate SNPs, the 90 strongest SNPs, or the 80 strongest SNPs according to the empirical score-space association measure S_j. Results are shown for $n = 1000$ and $n = 2000$, with $K = 10$ longitudinal exposures and $r = 3$ latent factors. Within each replicate, the same simulated dataset and retained FPCA representation are used across the three instrument-selection settings. Each box summarizes 100 simulation replicates.

703 participants. To examine sensitivity to this choice, we repeat the FA-MFMR analysis using progressively more stringent thresholds $p < 0.04, 0.03, 0.025, 0.02, 0.015, 0.01, 0.0075, 0.005$. The stricter sets are nested subsets of the original 231 instruments. For each SNP, the association with its assigned biomarker at visit V08 is re-evaluated within the primary analytic cohort. Importantly, NP3TOT is not used in constructing any of the alternative instrument sets. The analytic cohort, longitudinal biomarker observations, terminal NP3TOT definition, exposure scaling, FPCA variance-explained criterion, and model-fitting procedure are otherwise kept unchanged. The full instrument-dependent FA-MFMR procedure is then refitted under each feasible threshold.

For descriptive assessment of first-stage relevance, for SNP j we calculate the marginal V08 first stage statistic $F_j = \left(\frac{\widehat{\alpha}_j}{\text{se}(\widehat{\alpha}_j)}\right)^2$, where $\widehat{\alpha}_j$ is the estimated association between SNP j and its assigned biomarker at V08. These marginal statistics are used to describe how the strength distribution of the retained instruments changes with the selection threshold. We regard an alternative threshold as feasible for fitting the complete 13-biomarker functional model only when each biomarker retains at least one instrument and the total number of retained instruments is sufficient for the retained FPCA-score representation.

To quantify changes in the estimated coefficient functions, we compare each stricter-threshold estimate with the primary $p < 0.05$ estimate. For biomarker g and threshold c, the biomarker-specific functional RMSE is

$$\text{RMSE}_g(c) = \left[\frac{1}{30}\int_{50}^{80}\left\{\widehat{\beta}_g^{(c)}(t) - \widehat{\beta}_g^{(0.05)}(t)\right\}^2 dt\right]^{1/2}.$$

This quantity measures the functional discrepancy from the primary fitted coefficient function.

As the internal validation threshold becomes more stringent, the retained instrument set decreases substantially, from 231 SNPs under the primary $p < 0.05$ threshold to 153, 126, 105, 89, 70, and 51 SNPs at $p < 0.04, p < 0.03, p < 0.025, p < 0.02, p < 0.015$, and $p < 0.01$, respectively (Table S3 and Figure S34). At the same time, the median marginal V08 first-stage F-statistic increases from 4.85 under the primary threshold to 8.11 at $p < 0.01$, demonstrating the expected trade-off between instrument-set size and marginal first-stage strength. At $p < 0.0075$ and $p < 0.005$, only 36 and 28 SNPs remain, respectively, and only 12 of the 13 biomarkers retain instrument representation. We therefore do not fit the complete FA-MFMR model under these two thresholds.

Across the six feasible stricter thresholds, the median biomarker-specific functional RMSE ranges from 0.089 to 0.137, indicating that the typical biomarker-level discrepancy from the primary analysis remains relatively modest. However, sensitivity is heterogeneous across biomarkers (Figure S33). Monocyte count, serum glucose, diastolic blood pressure, and lymphocyte count remain particularly close to their primary $p < 0.05$ coefficient functions. In contrast, larger discrepancies occur for a subset of biomarkers, most notably white blood cell count and systolic blood pressure, with basophil and neutrophil counts also showing greater sensitivity than most of the remaining biomarkers.

The discrepancies do not increase monotonically as the threshold becomes more stringent.

Table S3: Sensitivity of the PPMI analysis to the internal V08 SNP-biomarker association threshold. The number of retained SNPs, number of biomarkers retaining instrument representation, median marginal V08 first-stage F-statistic, and functional discrepancy from the primary $p < 0.05$ analysis are reported for each threshold. Functional RMSE is calculated relative to the coefficient functions estimated in the primary analysis.

V08 threshold	Retained SNPs	Biomarkers represented	Median marginal F	Median functional RMSE
$p < 0.05$	231	13/13	4.85	0.000
$p < 0.04$	153	13/13	5.72	0.103
$p < 0.03$	126	13/13	6.30	0.108
$p < 0.025$	105	13/13	6.55	0.090
$p < 0.02$	89	13/13	6.93	0.137
$p < 0.015$	70	13/13	7.28	0.089
$p < 0.01$	51	13/13	8.11	0.094
$p < 0.0075$	36	12/13	9.13	-
$p < 0.005$	28	12/13	9.35	-

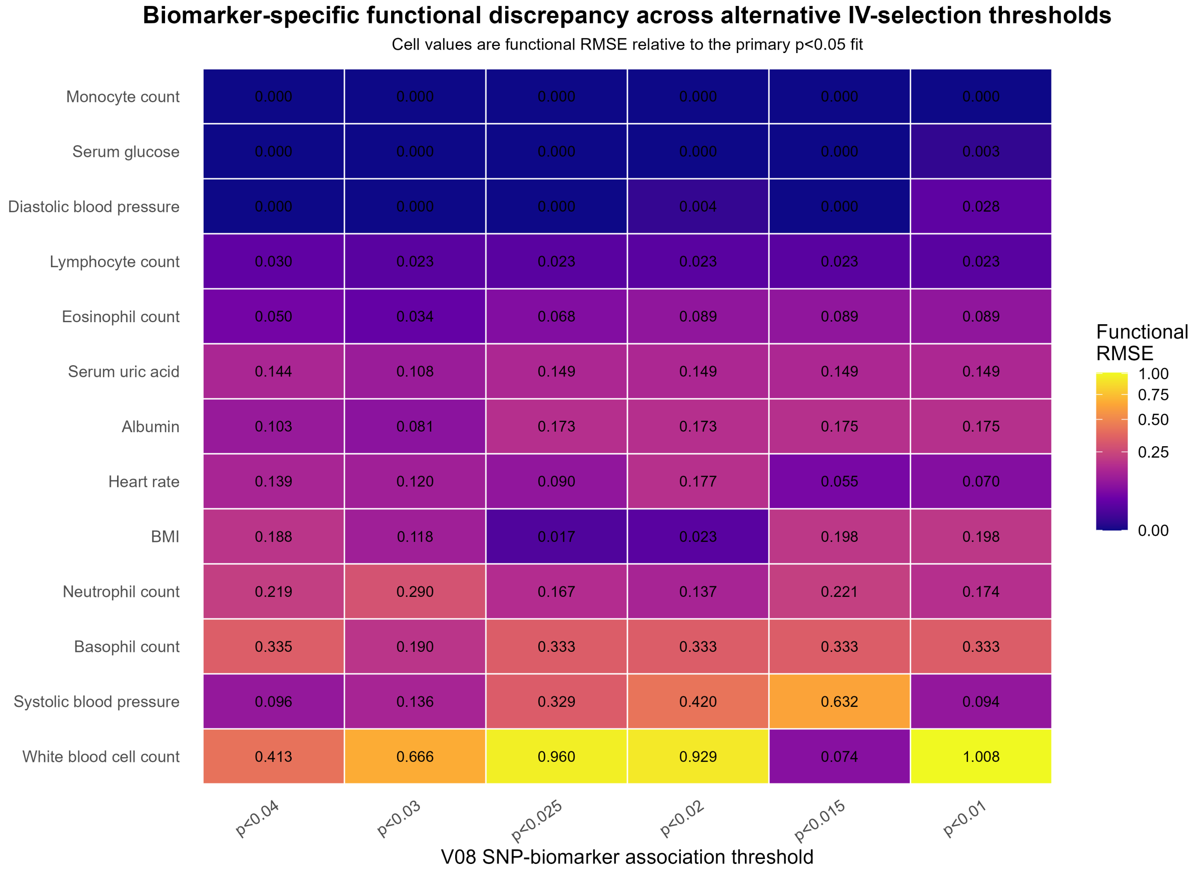

Figure S33: Biomarker-specific sensitivity of the PPMI coefficient functions to alternative internal instrument-selection thresholds. Each cell reports the functional RMSE between the coefficient function estimated under the indicated V08 SNP-biomarker association threshold and the corresponding coefficient function from the primary $p < 0.05$ analysis. Smaller values indicate closer agreement with the primary estimate. Only thresholds for which the complete 13-biomarker FA-MFMR model can be fitted are shown.

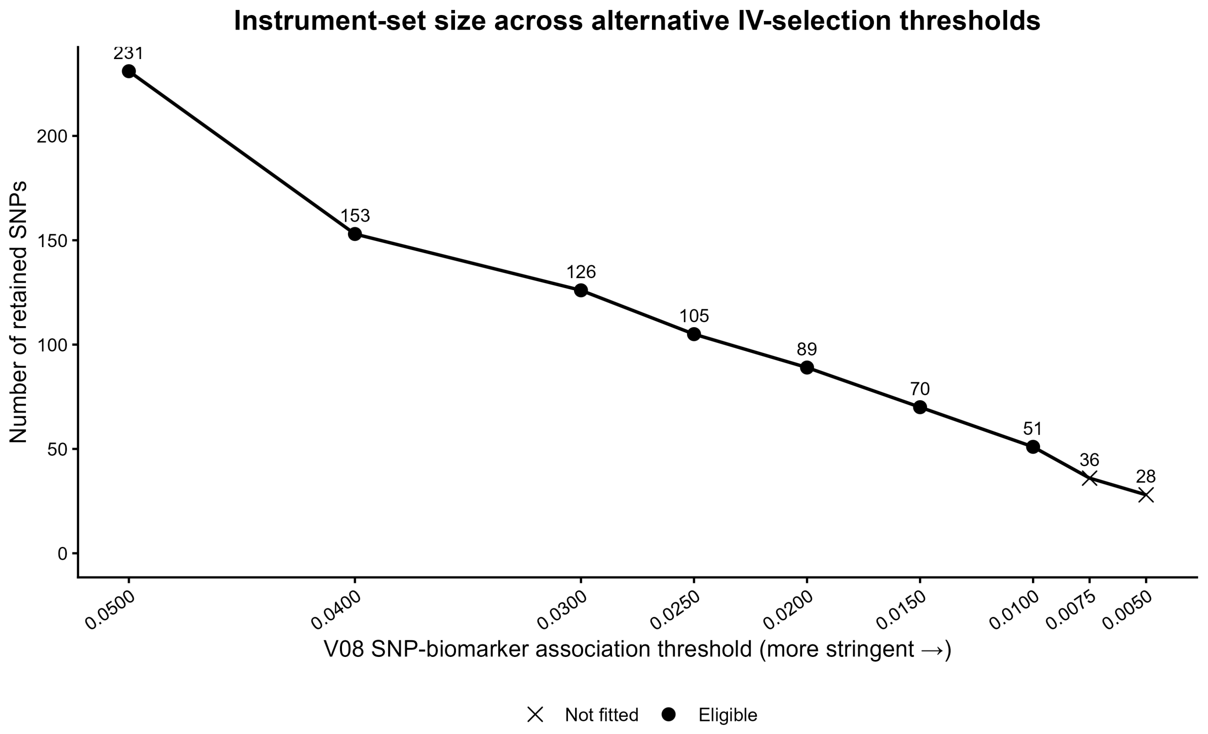

Figure S34: Instrument-set size under progressively more stringent V08 SNP–biomarker association thresholds in the PPMI analysis. Circles denote thresholds for which all 13 biomarkers retain instrument representation and the complete FA-MFMR model is fitted. Crosses denote thresholds for which at least one biomarker retains no instrument; these settings are not fitted.

For example, the white blood cell count coefficient function shows substantially different discrepancies across the alternative instrument sets, rather than a simple monotone deterioration with decreasing instrument number. This pattern indicates that sensitivity depends not only on the number or marginal strength of retained instruments but also on the composition of the instrument set and the functional-score directions supported by those instruments.

Overall, these analyses show that more stringent internal validation increases the marginal strength of the retained SNPs at the cost of substantially reduced instrument coverage. The estimated coefficient functions for several biomarkers remain close to the primary analysis across the feasible alternative thresholds, whereas a smaller subset of biomarkers is more sensitive to instrument selection. Together with the simulation results, these findings emphasize the trade-off between instrument strength and joint information for the multivariable functional-score system rather than supporting a uniformly optimal instrument-selection threshold.

S3: Implementation details of competing methods

All methods are applied to the same simulated datasets and genetic instruments within each replicate. Longitudinal exposures are represented using PACE/FPCA, with the number of retained components selected by a fraction-of-variance-explained threshold of 0.95. The resulting score-level estimates are mapped back through the corresponding FPCA eigenfunctions to obtain the estimated coefficient functions.

PACE+2SFRI. We adapt the functional two-stage residual-inclusion procedure of Cao et al. (Cao et al., 2016) to multiple longitudinal exposures. For each exposure, the PACE-reconstructed trajectory is centered and regressed on the genetic instruments in the first stage. The resulting functional residual process is represented by FPCA. In the second stage, the retained exposure scores and residual scores from all exposures are jointly included in the outcome regression, and the coefficients corresponding to the exposure scores are used to reconstruct the time-varying effect functions.

MPCMR. We extend the continuous-time MR procedure of Tian et al. (Tian et al., 2024) to multiple exposures by concatenating the retained FPCA scores across exposures. The combined score matrix, outcome, and common genetic instrument matrix are supplied to the original one-sample GMM estimation routine. The resulting coefficients are then partitioned by exposure and mapped back to the functional domain.

MFMR. The retained FPCA scores from all exposures are concatenated and treated jointly as endogenous regressors. Their coefficients are estimated by two-stage least squares using the genetic variants as instruments, implemented with ivreg in the R package AER, and are subsequently used to reconstruct the exposure-specific coefficient functions.

pMFMR. The concatenated FPCA score matrix is first projected onto the genetic-instrument space. Scores belonging to the same longitudinal exposure are treated as one group, and group SCAD is then applied using the R package grpreg, with the tuning parameter selected by 10-fold cross-validation. No latent-factor decomposition is performed, so pMFMR isolates the contribution of sparsity regularization without factor augmentation.

oracle-FA-MFMR. Oracle-FA-MFMR follows the same IV projection, factor adjustment, sparse estimation, and coefficient-function reconstruction as FA-MFMR, except that the number of latent factors is fixed at the true value used in the simulation rather than estimated from the data. It is therefore included only as a simulation benchmark for evaluating the effect of

data-driven factor-number selection.

References

Cao, Y., Rajan, S. S., and Wei, P. (2016). Mendelian randomization analysis of a time-varying exposure for binary disease outcomes using functional data analysis methods. *Genetic Epidemiology*, 40(8):744–755.

Kong, D., Xue, K., Yao, F., and Zhang, H. H. (2016). Partially functional linear regression in high dimensions. *Biometrika*, 103(1):147–159.

Ma, H., Shen, Z., Feng, X., and Liu, X. (2025). Sparsity learning via structured functional factor augmentation. *arXiv preprint arXiv:2501.02244*.

Tian, H., Patel, A., and Burgess, S. (2024). Estimating time-varying exposure effects through continuous-time modelling in Mendelian randomization. *Statistics in Medicine*, 43(27):5166–5181.
